# [3+2] Cycloaddition of alkyl aldehydes and alkynes enabled by photoinduced hydrogen atom transfer

**DOI:** 10.1038/s41467-022-32467-x

**Published:** 2022-08-12

**Authors:** Siya Le, Ji Li, Jian Feng, Zuxiao Zhang, Yihui Bai, Zheliang Yuan, Gangguo Zhu

**Affiliations:** Key Laboratory of the Ministry of Education for Advanced Catalysis Materials, Department of Chemistry, Zhejiang Normal University, 688 Yingbin Road, Jinhua, 321004 P. R. China

**Keywords:** Synthetic chemistry methodology, Photocatalysis

## Abstract

[3+2] Cycloaddition is a step- and atom-economic method for the synthesis of five-membered rings. Despite the great success of 1,3-dipolar cycloadditions, the radical [3+2] annulation of alkynes remains a formidable challenge. Herein, a photoinduced decatungstate-catalyzed [3+2] cycloaddition of various internal alkynes using abundant aliphatic aldehydes as a three-carbon synthon is developed, producing elaborate cyclopentanones in 100% atom economy with excellent site-, regio-, and diastereoselectivity under mild conditions. The catalytic cycle consists of hydrogen atom abstraction from aldehydes, radical addition, 1,5-hydrogen atom transfer, anti-Baldwin 5-endo-trig cyclization, and back hydrogen abstraction. The power of this method is showcased by the late-stage elaboration of medicinally relevant molecules and total or formal synthesis of (±)-β-cuparenone, (±)-laurokamurene B, and (±)-cuparene.

## Abstract (web summary)

In contrast to the prevalence of 1,3-dipolar cycloadditions, radical [3+2] annulations of alkynes are underexplored. Here, the authors describe [3+2] cycloadditions of various internal alkynes with readily accessible aliphatic aldehydes via photoinduced decatungstate catalysis.

## Introduction

**Fig. 1:**
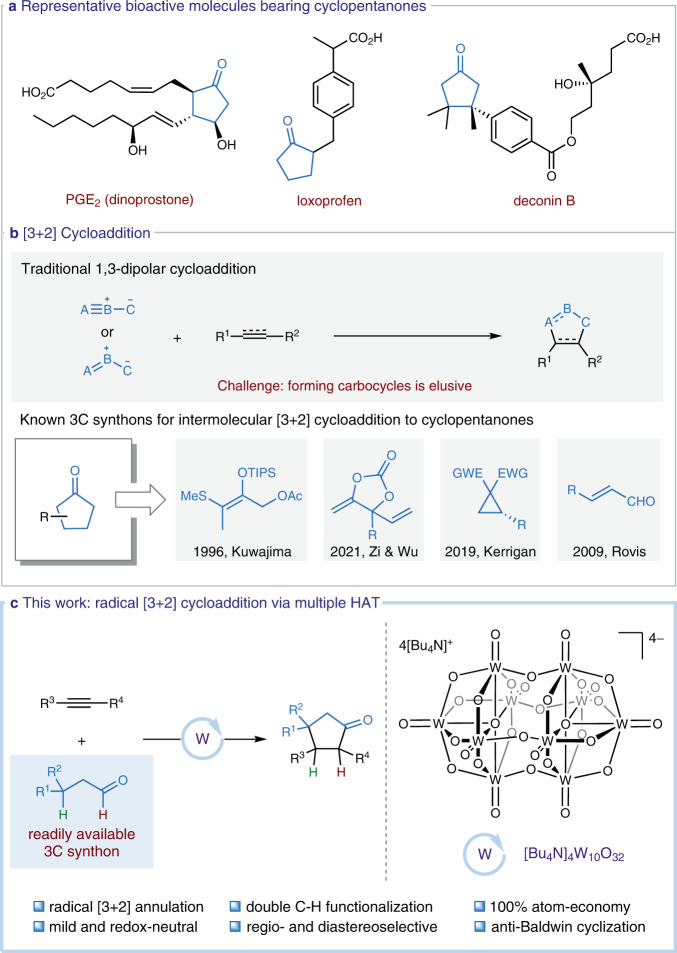
Background and summary of this work. 3C three carbon. TIPS triisopropylsilyl. EWG electron-withdrawing group. W tetra-*n*-butylammonium decatungstate. **a** Representative bioactive molecules bearing cyclopentanones. **b** Traditional [3+2] cycloaddition reactions. **c** This work, a radical [3+2] cycloaddition enabled by multiple HAT.

Cyclopentanones are important structural scaffolds embedded in a number of natural products, pharmaceuticals, and bioactive compounds, such as PGE_2_, loxoprofen, and deconin B (Fig. 1a)^1–3^. Meanwhile, they can serve as versatile intermediates in synthetic chemistry. Consequently, enormous efforts have been devoted to the construction of these motifs, including the ring expansion^4–6^, carbene insertion^7,8^, and intramolecular hydroacylation^9,10^. However, the aforementioned methods rely on the use of prefunctionalized substrates that require multistep synthesis, thus calling for the development of more straightforward and robust protocols.

The [3+2] cycloaddition represents a powerful means for assembling five-membered hetero- and carbocycles from readily attained starting materials (Fig. 1b). In this respect, the preparation of cyclopentanones has been rarely accomplished^11–19^, mainly due to the lack of suitable three-carbon (3C) synthons. By harnessing the in situ generated oxyallyl cations as a 1,3-dipole, Kuwajima reported a rapid assembly of cyclopentanones via the [3+2] cycloaddition of electron-rich and -neutral alkenes^11^. Using palladium-oxyallyl species as a 3C source, annulations of the more challenging nitroalkenes^12^, 1,3-dienes^13^, and acrylonitriles^14^ were successfully developed by the groups of Zi and Wu. Kerrigan and co-workers discovered a dual Lewis acid-catalyzed [3+2] carbocyclization of ketenes with donor-acceptor cyclopropanes^15^. Alternatively, Rovis disclosed that enals could serve as a 3C building block for the access to cyclopentanones via the *N*-heterocyclic carbene-catalyzed cascade cyclizations^16,17^. Despite these significant advances, it is still highly desirable to develop novel [3+2] annulation for the atom-economic and controllable synthesis of polysubstituted cyclopentanones, particularly using readily available 3C synthons. In light of recent developments on decatungstate-catalyzed C-H functionalization of aldehydes^20–33^ and our interest^34^ on 5-endo-trig radical cyclization of all-carbon systems, we envisioned that abundant aliphatic aldehydes could be exploited as an ideal 3C component for the [3+2] carbocyclization of alkynes via the hydrogen atom transfer (HAT)^35–40^ -induced double C-H functionalization (Fig. 1c).

In this work, we report a photoinduced tetra-*n*-butylammonium decatungstate (TBADT) catalyzed radical [3+2] cycloaddition of alkyl aldehydes and a wide range of alkynes, which provides a streamline and robust access to heavily functionalized cyclopentanones from readily attained feedstocks. In contrast to the traditional ionic protocols^11–18^, this single-electron-mediated process exhibits many advantages, such as mild and redox-neutral conditions, 100% atom economy, excellent site-, regio-, and diastereoselectivity, very broad substrate scope, and high efficiency, rendering it an attractive strategy for chemical synthesis and drug discovery.

## Results

### Reaction optimization

Optimization studies began with the reaction of commercially available alkyl aldehyde **1a** and alkyne **2a**. Upon treating the reaction mixture with 2 mol% of TBADT under irradiation of 40W, 390 nm purple LEDs in a 10:1 mixture of MeCN and H_2_O at 25 °C for 10 h, cyclopentanone **3** was isolated in 88% yield as a single regio- and diastereoisomer (Table 1, entry 1). Switching the HAT photocatalyst from TBADT to NaDT resulted in a slightly decreased yield (entry 2). Other reaction mediums, such as MeCN, DMSO, acetone, and DMF, proved to be less effective (entries 3–6). The controlled experiments revealed that both TBADT and light are essential for this transformation (entries 7 and 8).

**Table 1:** Optimization of reaction conditions


Entry	Deviations from above^a^	Yield (%)^b^
1	none	88
2	NaDT instead of TBADT	83
3	MeCN instead of MeCN/H_2_O	75
4	DMSO instead of MeCN/H_2_O	trace
5	acetone instead of MeCN/H_2_O	65
6	DMF instead of MeCN/H_2_O	43
7	without TBADT	0
8	without LED irradiation	0

TBADT tetra-*n*-butylammonium decatungstate, NaDT sodium decatungstate, MeCN acetonitrile, DMSO dimethylsulfoxide, DMF *N*,*N*-dimethylformamide.

^a^Reaction conditions: **1a** (0.4 mmol), 2**a** (0.2 mmol), TBADT (2 mol%), MeCN/H_2_O (10:1), purple LEDs, 390 nm, 25 °C, 10 h.

^b^Isolated yield.

### Examination of substrate scope

**Fig. 2:**
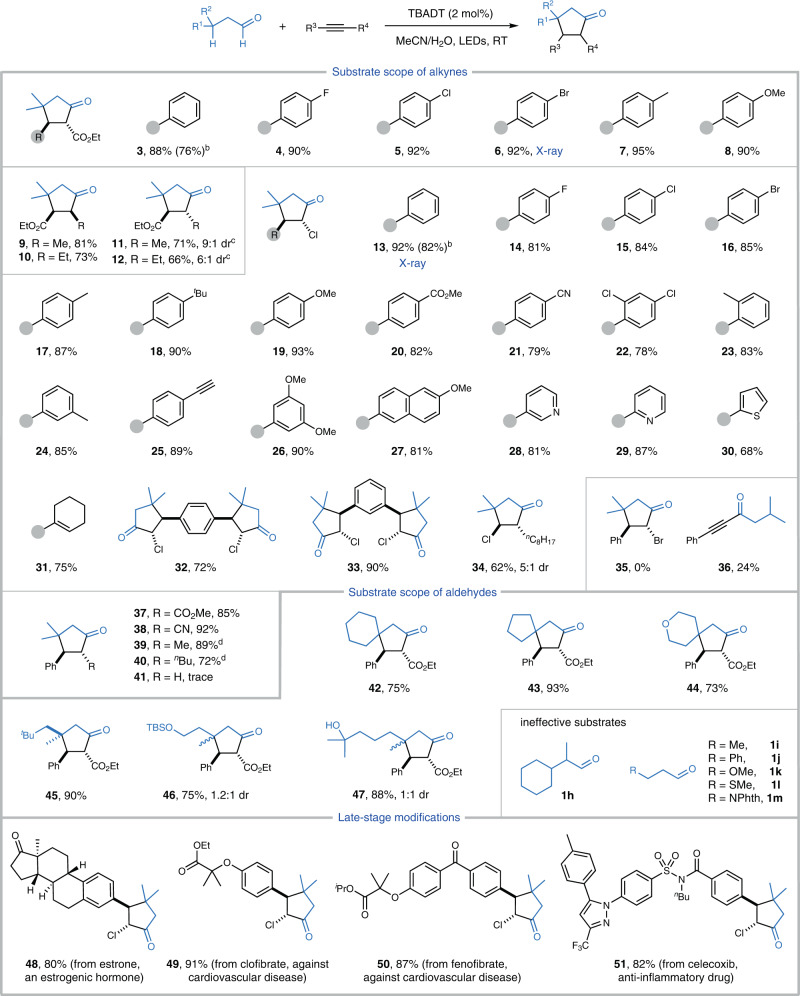
Scope of the reaction. Reaction conditions: see Table 1, entry 1; Isolated yields are given. Unless otherwise noted, the desired products were obtained with ≥10:1 dr selectivity. ^b^Yield of 1.0 mmol scale. ^c^(i) see Table 1, entry 1; (ii) NaOH (0.2 mmol), EtOH, RT, 5 h. ^d^(i) see Table 1, entry 1; (ii) K_2_CO_3_ (0.4 mmol), DMSO, 80 °C, 5 h. Dr diastereoisomer ratio. TBS *tert*-butyldimethylsilyl. RT room temperature.

Having established the optimized reaction conditions, we set about investigating the scope of this [3+2] annulation with various functionalized alkynes (Fig. 2). Arylalkynyl esters were first evaluated. Pleasingly, they worked well for this reaction and produced a set of decorated cyclopentanones, including those possessing fluorine, chlorine, or bromine atom on the benzene ring, in high yields with excellent regio- and diastereoselectivity (**4**-**8**). The *trans*-diastereoselectivity was confirmed by the single X-ray diffraction analysis of **6**. In contrast, the reaction of alkylalkynyl esters formed the kinetically favorable *cis*-2,3-disubstituted cyclopentanones **9** and **10** in high yields, whose structures were assigned by NMR measurements (see the Supporting Information for details). Polar effect, instead of the radical delocalization to the neighboring aryl group that dominates in the transformation of arylalkynes, may account for the reversed regioselectivity^41^. Of note, *trans*-2,3-disubstituted cyclopentanones **11** and **12** could be selectively obtained in satisfactory yields via a subsequent NaOH-promoted epimerization, further enhancing the utility of this method.

Given the versatile reactivity of carbon-halide bonds, we then tested the reactivity of alkynyl chlorides. To our delight, they served as efficient substrates to form various *trans*-2,3-disubstituted cyclopentanones in promising yields with high regio- and diastereoselectivity. A large number of functional groups, such as fluorine (**14**), chlorine (**15** and **22**), bromine (**16**), methoxyl (**19**, **26**, and **27**), ester (**20**), nitrile (**21**), and terminal alkyne (**25**) were well tolerated, leaving ample room for further functionalizations. The single X-ray diffraction crystallography identified the structure of **13**. This process was well amenable to chloroalkynes bearing heterocycles, such as pyridine and thiophene, which delivered cyclopentanones **28**-**30** in 68–87% yields, without erosion of the site-, regio-, and diastereoselectivity. In addition to arylalkynyl chlorides, we also evaluated the reaction of alkenyl counterpartners, a class of challenging substrates due to the potential radical addition to C-C double bonds. As an example, cyclohexenyl ethynylchloride produced the alkenyl cyclopentanone **31** in 75% yield, highlighting the high chemoselectivity of this reaction. Remarkably, the double [3+2] cycloaddition was quite successful, as demonstrated by the regio- and diastereoselective construction of **32** and **33**, in which eight new chemical bonds and two carbocycles were concurrently assembled. As demonstrated by the production of **34**, alkylalkynyl chlorides also served as the competent coupling partner, albeit with a decreased diastereoselectivity (dr = 5:1). Meanwhile, we examined the reaction of phenylethynyl bromide, however, it failed to provide the annulation product due to the formation of alkynyl ketone **36** via a radical addition/β-elimination sequence. On the other hand, alkynyl nitrile proved to be a viable substrate, exclusively forming the *trans*-2,3-disubstituted cyclopentanone **38** in nearly quantitative yield. The carbocyclization of 1-phenyl-1-propyne, a weakly activated alkyne, took place efficiently to furnish **39** in 89% yield, however, a low diastereoselectivity (dr = 1.9:1) was observed. After some trials, the dr value could be improved to >20:1 upon combining with a subsequent K_2_CO_3_-promoted epimerization, implying that the *trans*-diastereoselectivity is thermodynamically controlled. In contrast, terminal alkynes were sluggish for this annulation (**41**).

The applicability of this reaction with respect to aldehydes was briefly examined. A set of aliphatic aldehydes with tertiary C-H bonds reacted uneventfully to afford the synthetically important but challenging spirocyclopentanones **42**-**44** in 73–93% yields with high diastereoselectivity. Cyclopentanone **45**, bearing three continuous stereocenters, was selectively constructed under the reaction conditions. Substitution of the aldehyde with OTBS and OH groups had no detrimental effect on this cycloaddition, delivering elaborate cyclopentanones **46** and **47** in satisfactory yields. In contrast, 2-cyclohexylpropanal (**1h**) and aldehydes with a set of secondary C-H bonds (**1i**-**1m**) were almost unreactive under the identical conditions.

Then, we performed the late-stage modifications of drug-related compounds via this protocol. In particular, estrone, an estrogenic hormone, was converted into cyclopentanone **48** in 80% yield; clofibrate and fenofibrate, two cardiovascular drugs, were transformed to **49** and **50** in 91% and 87% yield, respectively; celecoxib, an anti-inflammatory agent, provided cyclopentanone **51** in high yield.

### Synthetic applications

**Fig. 3:**
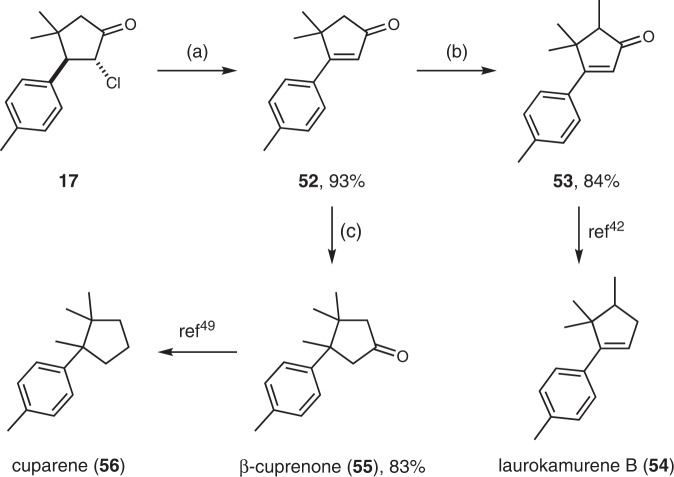
Synthetic applications. Reaction conditions: **a** **17**, K_2_CO_3_, DMF, 80 °C; **b** **52**, LiHMDS, MeI, THF, −78 °C to RT; **c** **52**, Ni(acac)_2_, AlMe_3_, THF, RT. LiHMDS lithium bis(trimethylsilyl)amide.

To illustrate the synthetic utility of this radical [3+2] process, 2-chlorocyclopentanone **17** was treated with K_2_CO_3_ in DMF at 80 °C (Fig. 3). As a result, cyclopentenone **52** was generated via HCl elimination in excellent yield. A subsequent methylation using LiHMDS and MeI delivered **53** in 84% yield. Since its transformation to the antifungal and cytotoxic natural product laurokamurene B (**54**)^41^ has been documented before, the formal synthesis of (±)-**54**^42–45^ was thus achieved. Additionally, Ni-catalyzed conjugate addition of AlMe_3_ to **52** provided 83% yield of β-cuprenone (**55**)^46–50^, a cyclopentanoid sesquiterpene used for the treatment of fungal infection, coughs, cancer, and hemorrhages. The total synthesis developed here requires only two steps from **17** and produces **55** in 77% overall yield, which compares favorably with the previous reports on the synthesis of (±)-**55**^47,48^. Another small-molecule natural product, cuparene (**56**), can also be accessible via the Wolff-Kishner reduction of **55**^49^.

### Mechanistic investigations

**Fig. 4:**
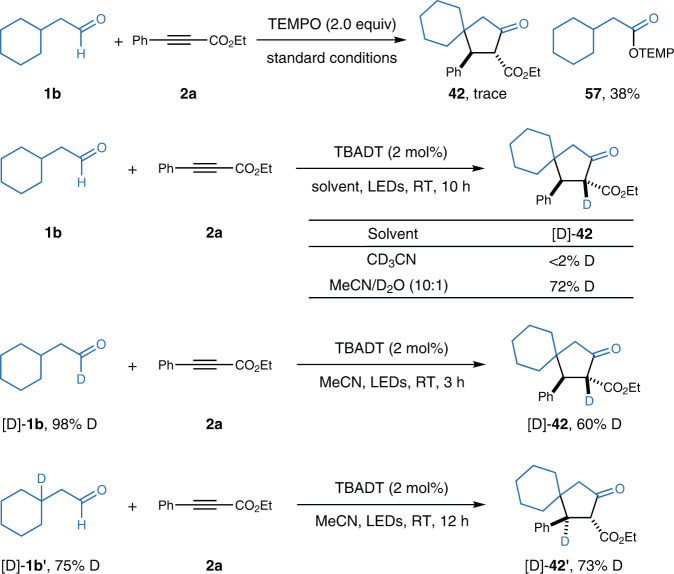
Mechanistic studies. TEMPO 2,2,6,6-tetramethylpiperidinooxy.

To confirm the in-situ formation of acyl radicals, the model reaction was conducted in the presence of 2.0 equiv of TEMPO (Fig. 4). As a result, the TEMPO-adduct **57**, instead of cyclopentanone **42**, was isolated in 38% yield. Some deuterium-labeling experiments were then conducted. Almost no deuterium incorporation was observed with CD_3_CN as the solvent, while [D]-**42** was formed with 72% deuterium incorporation by running the reaction in a mixture of MeCN and D_2_O. In contrast, the [3+2] annulation between [D]-**1b** (98% D) and **2a** in MeCN produced [D]-**42** with 60% deuterium incorporation, supporting that the hydrogen atom at the C2 position of cyclopentanones comes from aldehydes. Subjection of [D]-**1b’** (75% D) to the identical conditions resulted in the generation of [D]-**42'** (73% D), which is consistent with an intramolecular 1,5-HAT.

**Fig. 5:**
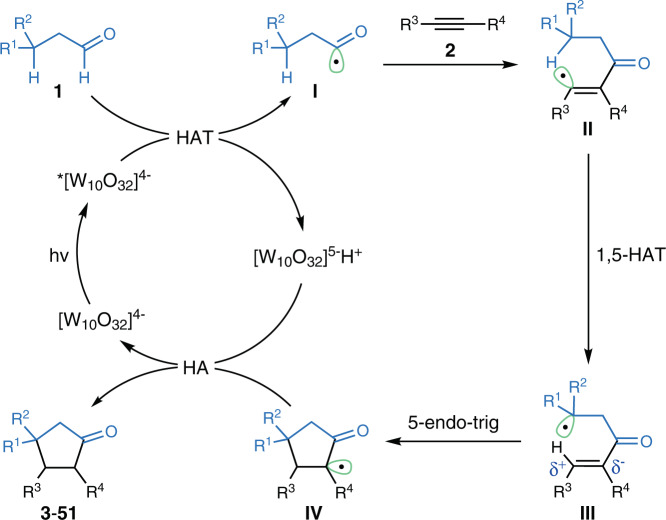
Proposed mechanism. HA hydrogen abstraction.

Based on the above results and previous reports^20–33^, a plausible mechanism for this TBADT-catalyzed [3+2] cycloaddition is proposed in Fig. 5. Initially, the excited-state catalyst *[W_10_O_32_]^4−^, obtained by photoexcitation of the decatungstate anion at 390 nm, abstracts a hydrogen atom from aldehydes to form acyl radicals **I** and [W_10_O_32_]^5−^H^+^. Subsequent radical addition to alkynes followed by 1,5-HAT of vinyl radicals^51–68^ generates nucleophilic alkyl radicals **III**. Then, a polarity-matched addition at the β-carbon atom leads to radicals **IV**. Of course, spin delocalization to the adjacent carbonyl group may also facilitate the uncommon 5-endo-trig cyclization. Back hydrogen abstraction from the reduced form of the tungstate anion [W_10_O^32^]^5-^H^+^ produces **3**–**51**, accompanied by restoring the TBADT catalyst.

## Discussion

We have developed a radical [3+2] annulation of alkynes using readily accessible alkyl aldehydes as a 3C building block and TBADT as a photocatalyst. The reaction occurs under mild, redox-neutral conditions, producing a diverse array of polysubstituted cyclopentanones in 100% atom economy with high efficiency and excellent site-, regio-, and diastereoselectivity. Preliminary studies indicate a mechanism involving an intermolecular HAT, radical addition, 1,5-HAT, unusual 5-endo-trig cyclization, and back hydrogen atom abstraction. The synthetic utility of this protocol is well illustrated by the late-stage derivatizations of drug-relevant compounds, total synthesis of (±)-β-cuprenone, and formal syntheses of (±)-laurokamurene B and (±)-cuparene, thus providing an appealing method for the fast construction of biologically active molecules and natural products.

## Methods

### Procedure for the TBADT-catalyzed [3+2] cycloaddition of alkyl aldehydes and alkynes

To a mixture of TBADT (10.6 mg, 0.004 mmol) in 2 mL of MeCN/H_2_O (v:v = 10:1) was added **1a** (34.4 mg, 0.4 mmol) and **2a** (34.8 mg, 0.2 mmol) under nitrogen atmosphere. After 10 h of irradiation with purple LEDs (100% intensity, Kessil PR160, 40 W, 390 nm, light irradiance at a distance of 3 cm: 76.41 mW/cm^2^, and the light irradiance at the reaction site: 4.62 mW/cm^2^), the reaction mixture was quenched with water, extracted with EtOAc, washed with brine, dried over anhydrous Na_2_SO_4_, and concentrated. The resulting residue was purified via column chromatography on silica gel to afford the desired product.

## Supplementary information

Supplementary Information

## Data Availability

Detailed experimental procedures and characterization of new compounds can be found in the Supplementary Information. Crystallographic data have been deposited at the Cambridge Crystallographic Data Centre as CCDC 2164582 (**6**) and CCDC 2164583 (**13**). These data can be obtained free of charge from The Cambridge Crystallographic Data Centre via www.ccdc.cam.ac.uk/data_request/cif. Crystal data are also provided in Supplementary Information. Further relevant data are available from the corresponding author upon request.
