# Supplementary material for: [3+2] Cycloaddition of alkyl aldehydes and alkynes enabled by photoinduced hydrogen atom transfer
Source: Nat Commun. 2022 Aug 12;13:4734. doi: 10.1038/s41467-022-32467-x (PMC9374768; doi:10.1038/s41467-022-32467-x)
Supplement: Supplementary file 1 — Supplementary Information [file 41467_2022_32467_MOESM1_ESM.pdf]

Supplementary Information to

[3+2] Cycloaddition of alkyl aldehydes and alkynes enabled by photoinduced hydrogen atom transfer

Siya Le, Ji Li, Jian Feng, Zuxiao Zhang, Yihui Bai, Zheliang Yuan & Gangguo Zhu*

Key Laboratory of the Ministry of Education for Advanced Catalysis Materials, Department of Chemistry, Zhejiang Normal University, 688 Yingbin Road, Jinhua 321004, P. R. China

*E-Mail: gangguo@zjnu.cn

Table of contents

1. Supplementary methods……3

1.1 General information……3

1.2 General procedure for the TBADT-catalyzed [3+2] cycloaddition of alkyl aldehydes with alkynes and characterization data of products……3

1.3 Experimental procedure for the transformation of **17** to **52**……23

1.4 Experimental procedure for the transformation of **52** to **53**……23

1.5 Experimental procedure for the transformation of **52** to **55**……24

1.6 Mechanistic experiments……24

1.7 Crystallographic data……31

2. Supplementary figures……34

3. Supplementary references……90

1. Supplementary methods

1.1 General information

Unless otherwise noted, materials obtained from commercial suppliers were used directly without further purification. Chloroalkynes were prepared according to the method reported in the literature.[1] Melting points reported here were measured by a melting point instrument and were uncorrected. ^{1}H, ^{13}C, and ^{19}F NMR spectra were measured on a 600 MHz or 400 MHz NMR spectrometer. Chemical shifts are given in parts per million on the delta (δ) scale, and the coupling constants are given in hertz. ^{1}H NMR chemical shifts were determined relative to the internal standard tetramethylsilane (TMS) at 0.00 ppm, ^{13}C NMR shifts were determined relative to the residual solvent peaks of $CDCl_3$ at δ 77.00 ppm, and ^{19}F NMR chemical shifts were determined relative to the outside standard $CFCl_3$ at δ 0.00 ppm. High-resolution mass spectrometry (HRMS) analysis was carried out using a TOF MS instrument with an ESI source. Flash column chromatography was carried out on the silica gel (200-300 mesh). The photo reaction set-up (SCI-PCRS-3-220) was purchased from Anhui Kemi machinery technology Co., Ltd.

1.2 General procedure for the TBADT-catalyzed [3+2] cycloaddition of alkyl aldehydes with alkynes and characterization data of products

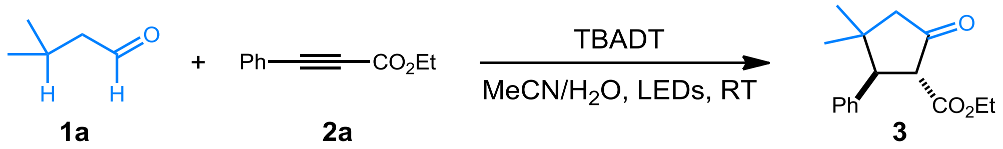

To a mixture of TBADT (10.6 mg, 0.004 mmol) in 2 mL of $MeCN/H_2O$ (v:v = 10:1) was added **1a** (34.4 mg, 0.4 mmol) and **2a** (34.8 mg, 0.2 mmol) under nitrogen atmosphere. After 10 h of irradiation with purple LEDs (100% intensity, Kessil PR160, 40 W, 390 nm, light irradiance at a distance of 3 cm: 76.41 mW/cm^2, and the light irradiance at the reaction site: 4.62 mW/cm^2), the reaction mixture was quenched with water, extracted with EtOAc, washed with brine, dried over anhydrous Na_2SO_4, and concentrated; Column chromatography on silica gel (petroleum ethers/EtOAc = 40:1) gave 46 mg (88% yield) of **3** as a colorless oil; The keto tautomer/enol tautomer > 20:1; Dr of the keto tautomer > 20:1; **^{1}H NMR (400 MHz, $CDCl_3$)** data of the keto tautomer δ 7.36-7.22 (m, 5H), 4.16-4.03 (m, 2H), 3.81 (d, J = 12.9 Hz, 1H), 3.64 (d, J = 13.0 Hz, 1H), 2.47 (d, J = 18.2 Hz, 1H), 2.41 (d, J = 18.3 Hz, 1H), 1.20-1.15 (m, 6H), 0.80 (s, 3H); **^{13}C NMR**

(101 MHz, $CDCl_3$) data of the keto tautomer δ 210.0, 168.7, 136.0, 128.6, 128.3, 127.4, 61.5, 58.5, 56.0, 54.6, 38.5, 27.3, 22.9, 14.1; **HRMS (ESI)** *m*/*z*: $[M + Na]^+$ Calcd for $C_{16}H_{20}O_3+Na^+$: 283.1305; Found 283.1295.

Scale-up synthesis of 3.

To a mixture of TBADT (53.5 mg, 0.02 mmol) in 10 mL of $MeCN/H_2O$ (v:v = 10:1) was added **1a** (258 mg, 3.0 mmol) and **2a** (174 mg, 1.0 mmol) under nitrogen atmosphere. After 15 h of irradiation with purple LEDs (100% intensity, Kessil PR160, 40 W, 390 nm, light irradiance at a distance of 3 cm: 76.41 mW/cm^2, and the light irradiance at the reaction site: 4.62 mW/cm^2), the reaction mixture was quenched with water, extracted with EtOAc, washed with brine, dried over anhydrous Na_2SO_4, and concentrated. Column chromatography on silica gel (petroleum ethers/EtOAc = 40:1) gave 197 mg (76% yield) of **3** as a colorless oil.

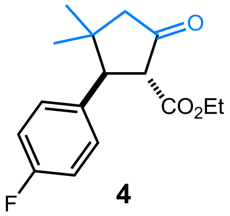

4

Compound 4: 10 h, 50 mg, 90% yield, colorless oil; The keto tautomer/enol tautomer = 10:1; Dr of the keto tautomer > 20:1; Flash column chromatography conditions: petroleum ethers/EtOAc = 40:1; **^{1}H NMR (400 MHz, $CDCl_3$)** data of the keto tautomer δ 7.19 (dd, *J* = 8.6, 5.4 Hz, 2H), 7.06-7.02 (m, 2H), 4.18-4.06 (m, 2H), 3.74 (d, *J* = 13.0 Hz, 1H), 3.61 (d, *J* = 13.0 Hz, 1H), 2.46 (d, *J* = 18.7 Hz, 1H), 2.41 (d, *J* = 18.6 Hz, 1H), 1.19 (t, *J* = 7.1 Hz, 3H), 1.17 (s, 3H), 0.80 (s, 3H); **^{13}C NMR (101 MHz, $CDCl_3$)** data of the keto tautomer δ 209.4, 168.6, 162.1 (d, *J* = 245.9 Hz), 131.8 (d, *J* = 3.4 Hz), 130.0 (d, *J* = 8.0 Hz), 115.2 (d, *J* = 21.2 Hz), 61.6, 58.7, 55.3, 54.4, 38.4, 27.2, 22.8, 14.1; **^{19}F NMR (565 MHz, $CDCl_3$)** δ -115.4; **HRMS (ESI)** *m*/*z*: $[M + H]^+$ Calcd for $C_{16}H_{19}FO_3+H^+$: 279.1391; Found 279.1388.

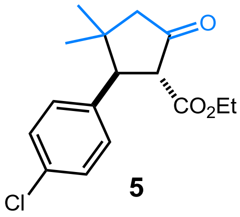

5

Compound 5: 10 h, 54 mg, 92% yield, colorless oil; The keto tautomer/enol tautomer = 10:1; Dr of the keto tautomer > 20:1; Flash column chromatography conditions: petroleum ethers/EtOAc = 40:1; **^{1}H NMR (400 MHz, $CDCl_3$)** data of the keto tautomer δ 7.34 (d, J = 8.4 Hz, 2H), 7.18 (d, J = 8.4 Hz, 2H), 4.20-4.04 (m, 2H), 3.76 (d, J = 13.0 Hz, 1H), 3.62 (d, J = 13.0 Hz, 1H), 2.46 (d, J = 18.7 Hz, 1H), 2.42 (d, J = 18.7 Hz, 1H), 1.24-1.17 (m, 6H), 0.81 (s, 3H); **^{13}C NMR (101 MHz, $CDCl_3$)** data of the keto tautomer δ 209.3, 168.5, 134.6, 133.2, 129.8, 128.5, 61.6, 58.5, 55.4, 54.4, 38.4, 27.2, 22.8, 14.1; **HRMS (ESI)** m/z: $[M + Na]^+$ Calcd for $C_{16}H_{19}ClO_3$+Na^+: 317.0915; Found 317.0904.

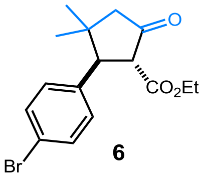

6

Compound 6: 10 h, 62 mg, 92% yield, white solid, mp 108-109 °C; The keto tautomer/enol tautomer = 10:1; Dr of the keto tautomer > 20:1; Flash column chromatography conditions: petroleum ethers/EtOAc = 40:1; **^{1}H NMR (400 MHz, $CDCl_3$)** data of the keto tautomer δ 7.48 (d, J = 8.3 Hz, 2H), 7.11 (d, J = 8.4 Hz, 2H), 4.18-4.03 (m, 2H), 3.74 (d, J = 13.0 Hz, 1H), 3.59 (d, J = 13.0 Hz, 1H), 2.46 (d, J = 18.8 Hz, 1H), 2.42 (d, J = 19.2 Hz, 1H), 1.19 (t, J = 7.2 Hz, 3H), 1.17 (s, 3H), 0.80 (s, 3H); **^{13}C NMR (101 MHz, $CDCl_3$)** data of the keto tautomer δ 209.2, 168.4, 135.2, 131.5, 130.2, 121.3, 61.6, 58.4, 55.5, 54.4, 38.4, 27.2, 22.8, 14.1; **HRMS (ESI)** m/z: $[M + H]^+$ Calcd for $C_{16}H_{19}BrO_3$+H^+: 339.0590; Found 339.0593. The single X-ray data were deposited with the Cambridge Crystallographic Data Centre as CCDC **2164582**.

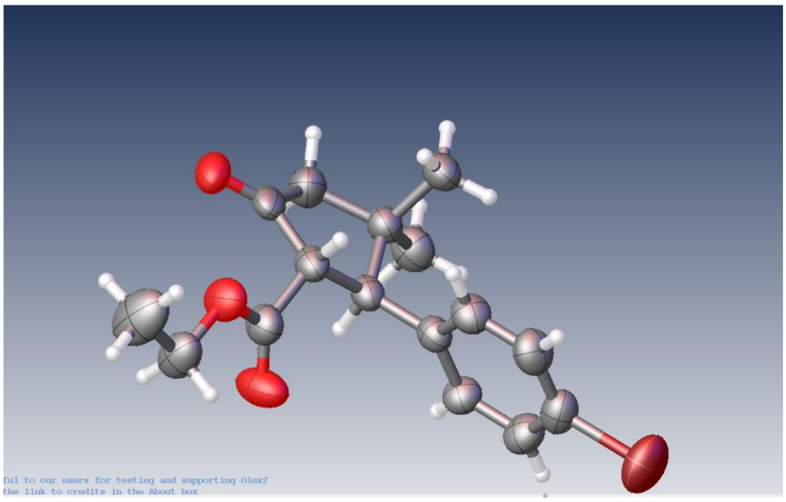

Supplementary Fig. 1. Crystal structure of **6**.

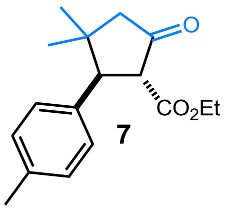

7

Compound 7: 10 h, 52 mg, 95% yield, colorless oil; The keto tautomer/enol tautomer > 20:1; Dr of the keto tautomer > 20:1; Flash column chromatography conditions: petroleum ethers/EtOAc = 40:1; **^{1}H NMR (400 MHz, $CDCl_3$)** data of the keto tautomer δ 7.16-7.10 (m, 4H), 4.16-4.04 (m, 2H), 3.78 (d, J = 13.0 Hz, 1H), 3.60 (d, J = 13.0 Hz, 1H), 2.45 (d, J = 18.2 Hz, 1H), 2.40 (d, J = 18.2 Hz, 1H), 2.34 (s, 3H), 1.18 (t, J = 7.1 Hz, 3H), 1.17 (s, 3H), 0.79 (s, 3H); **^{13}C NMR (101 MHz, $CDCl_3$)** data of the keto tautomer δ 210.1, 168.8, 136.9, 133.0, 129.0, 128.4, 61.5, 58.6, 55.5, 54.6, 38.4, 27.3, 22.9, 21.1, 14.1; **HRMS (ESI)** *m*/*z*: $[M + H]^+$ Calcd for $C_{17}H_{22}O_3+H^+$: 275.1642; Found 275.1635.

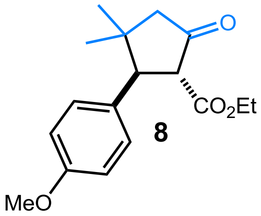

8

Compound 8: 16 h, 52 mg, 90% yield, colorless oil; The keto tautomer/enol tautomer > 20:1; Dr of the keto tautomer > 20:1; Flash column chromatography conditions: petroleum ethers/EtOAc = 30:1; **^{1}H NMR (400 MHz, $CDCl_3$)** data of the keto tautomer δ 7.14 (d, J = 8.6 Hz, 2H), 6.88 (d, J = 8.6 Hz, 2H), 4.16-4.05 (m, 2H), 3.81 (s, 3H), 3.75 (d, J = 13.0 Hz, 1H), 3.58 (d, J = 13.0 Hz, 1H), 2.45 (d, J = 18.3 Hz, 1H), 2.40 (d, J = 18.3 Hz, 1H), 1.19 (t, J = 7.2 Hz, 3H), 1.16 (s, 3H), 0.79 (s, 3H); **^{13}C NMR (101 MHz, $CDCl_3$)** data of the keto tautomer δ 210.1, 168.8, 158.8, 129.5, 128.0, 113.6, 61.5, 58.8, 55.3, 55.2, 54.5, 38.4, 27.2, 22.9, 14.1; **HRMS (ESI)** *m*/*z*: $[M + H]^+$ Calcd for $C_{17}H_{22}O_4+H^+$: 291.1591; Found 291.1589.

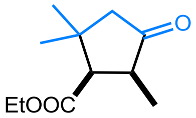

9

Compound 9: The title compound was prepared according to the general procedure with the addition of $Na_2WO_4 \cdot 2H_2O$ (5.3 mg, 8 mol%) to give 32 mg (81% yield) as a colorless oil after 15 h, dr > 10:1; Flash column chromatography conditions: petroleum ethers/EtOAc = 40:1; **^{1}H NMR**

(600 MHz, $CDCl_3$) δ 4.16 (q, J = 7.1 Hz, 2H), 2.84 (d, J = 7.7 Hz, 1H), 2.69 (dq, J = 3.5, 7.0 Hz, 1H), 2.47 (d, J = 18.6 Hz, 1H), 2.11 (d, J = 18.7 Hz, 1H), 1.27 (t, J = 7.1 Hz, 3H), 1.21 (s, 3H), 1.18 (s, 3H), 1.09 (d, J = 7.0 Hz, 3H); **^{13}C NMR (151 MHz, $CDCl_3$)** δ 217.9, 172.9, 60.4, 57.7, 49.5, 45.8, 36.6, 30.0, 25.8, 14.3, 10.2; **HRMS (ESI)** *m/z*: $[M + H]^+$ Calcd for $C_{11}H_{18}O_3$+H^+: 199.1329; Found 199.1320. The regio- and diastereoselectivity was determined by the $^3J_{H2\text{-}H3}$ coupling constant, HMBC, and NOE measurements.

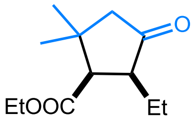

10

Compound 10: The title compound was prepared according to the procedure described for **9** to give 31 mg (73% yield) as a colorless oil after 15 h, dr = 10:1; Flash column chromatography conditions: petroleum ethers/EtOAc = 40:1; **^{1}H NMR (600 MHz, $CDCl_3$)** δ 4.19-4.11 (m, 2H), 2.90 (d, J = 7.6 Hz, 1H), 2.52-2.45 (m, 2H), 2.10 (d, J = 18.6 Hz, 1H), 1.95-1.88 (m, 1H), 1.65-1.64 (m, 1H), 1.26 (t, J = 7.1 Hz, 3H), 1.20 (s, 3H), 1.17 (s, 3H), 0.96 (t, J = 7.6 Hz, 3H); **^{13}C NMR (151 MHz, $CDCl_3$)** δ 217.2, 172.9, 60.3, 55.9, 53.3, 49.8, 36.6, 29.9, 25.8, 19.3, 14.2, 12.6; **HRMS (ESI)** *m/z*: $[M + H]^+$ Calcd for $C_{12}H_{20}O_3$+H^+: 213.1485; Found 213.1483.

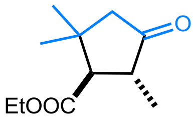

11

Compound 11: To a mixture of TBADT (10.6 mg, 0.004 mmol) in 2 mL of $MeCN/H_2O$ (v:v = 10:1) was added **1a** (34.4 mg, 0.4 mmol) and **2g** (22.5 mg, 0.2 mmol) under nitrogen atmosphere. After 15 h of irradiation with purple LEDs (100% intensity, Kessil PR160, 40 W, 390 nm, light irradiance at a distance of 3 cm: 76.41 mW/cm^2, and the light irradiance at the reaction site: 4.62 mW/cm^2), the reaction mixture was quenched with water, extracted with EtOAc, washed with brine, dried over anhydrous Na_2SO_4, and concentrated to give the crude product **9**. To the crude product **9** in 2 mL of EtOH was added NaOH (8.1 mg, 0.2 mmol). After stirring at 25 °C for 5 h, the reaction mixture was quenched with 1 M of aqueous HCl, extracted with EtOAc, washed with brine, dried over anhydrous Na_2SO_4, and concentrated. Column chromatography on silica gel (petroleum

ethers/EtOAc = 40:1) gave 28 mg (71% yield from **2g**) of **11** as a colorless oil, dr = 9:1; **^{1}H NMR (600 MHz, $CDCl_3$)** data of the *trans*-isomer δ 4.43-3.95 (m, 2H), 3.15-2.72 (m, 1H), 2.48 (d, J = 11.6 Hz, 1H), 2.29 (dd, J = 18.4, 1.8 Hz, 1H), 2.17 (d, J = 18.4 Hz, 1H), 1.33 (s, 3H), 1.30 (t, J = 7.1 Hz, 3H), 1.09 (d, J = 7.0 Hz, 3H), 0.98 (s, 3H); **^{13}C NMR (151 MHz, $CDCl_3$)** data of the *trans*-isomer δ 217.8, 171.9, 60.7, 59.3, 53.7, 45.5, 37.2, 28.9, 24.0, 14.4, 13.9; **HRMS (ESI)** *m*/*z*: $[M + H]^+$ Calcd for $C_{11}H_{18}O_3$+H^+: 199.1329; Found 199.1330.

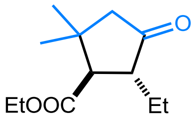

12

Compound 12: The title compound was prepared according to the procedure described for **11** to give 28 mg (66% yield from **2h**) as a colorless oil, dr = 6:1; Flash column chromatography conditions: petroleum ethers/EtOAc = 50:1; **^{1}H NMR (600 MHz, $CDCl_3$)** data of the *trans*-isomer δ 4.27-4.10 (m, 2H), 2.79-2.75 (m, 1H), 2.60 (d, J = 11.2 Hz, 1H), 2.26 (dd, J = 17.9, 1.8 Hz, 1H), 2.13 (d, J = 17.9 Hz, 1H), 1.80-1.62 (m, 1H), 1.55-1.40 (m, 1H), 1.31 (s, 3H), 1.29 (t, J = 7.2 Hz, 3H), 0.95 (s, 3H), 0.87 (t, J = 7.5 Hz, 3H); **^{13}C NMR (151 MHz, $CDCl_3$)** data of the *trans*-isomer δ 217.7, 172.3, 60.7, 56.6, 54.6, 51.4, 37.3, 28.8, 24.1, 22.8, 14.4, 11.1; **HRMS (ESI)** *m*/*z*: $[M + H]^+$ Calcd for $C_{12}H_{20}O_3$+H^+: 213.1485; Found 213.1486.

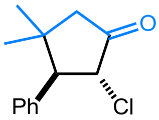

13

Compound *13:* 10 h, 41 mg, 92% yield, white solid, mp 121-122 °C, dr > 20:1; Flash column chromatography conditions: petroleum ethers/EtOAc = 50:1; **^{1}H NMR (400 MHz, $CDCl_3$)** δ 7.42-7.32 (m, 3H), 7.25-7.20 (m, 2H), 4.72 (d, J = 12.7 Hz, 1H), 3.23 (d, J = 12.8 Hz, 1H), 2.54 (dd, J = 18.9, 1.2 Hz, 1H) , 2.44 (d, J = 18.9 Hz, 1H), 1.19 (s, 3H), 0.86 (s, 3H); **^{13}C NMR (101 MHz, $CDCl_3$)** δ 209.2, 134.6, 128.6, 128.5, 127.7, 61.7, 61.6, 52.1, 37.8, 28.3, 23.2; **HRMS (ESI)** *m*/*z*: $[M + Na]^+$ Calcd for $C_{13}H_{15}ClO$+Na^+: 245.0704; Found 245.0693. The single X-ray data were deposited with the Cambridge Crystallographic Data Centre as CCDC **2164583**.

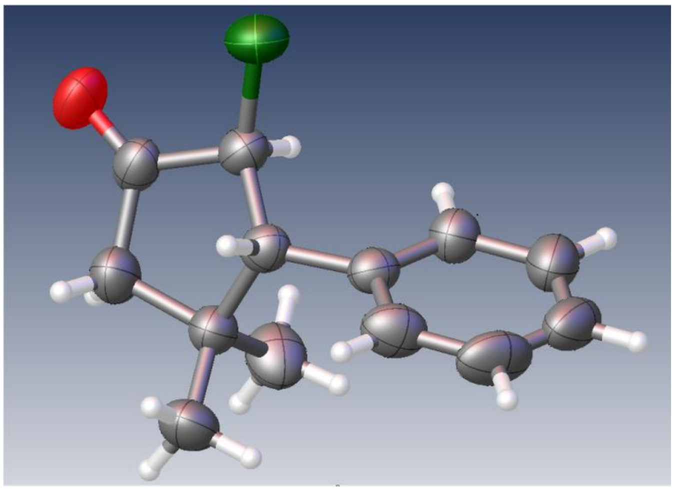

Supplementary Fig. 2. Crystal structure of **13**.

Scale-up synthesis of 13.

To a mixture of TBADT (53.5 mg, 0.02 mmol) in 10 mL of MeCN/H_2O (v:v = 10:1) was added **1a** (258 mg, 3.0 mmol) and **2i** (137 mg, 1.0 mmol) under nitrogen atmosphere. After 15 h of irradiation with purple LEDs (100% intensity, Kessil PR160, 40 W, 390 nm, light irradiance at a distance of 3 cm: 76.41 mW/cm^2, and the light irradiance at the reaction site: 4.62 mW/cm^2), the reaction mixture was quenched with water, extracted with EtOAc, washed with brine, dried over anhydrous Na_2SO_4, and concentrated. Column chromatography on silica gel (petroleum ethers/EtOAc = 50:1) gave 182 mg (82% yield) of **13** as a colorless oil.

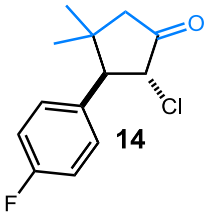

14

Compound 14: 10 h, 39 mg, 81% yield, white solid, mp 123-124 ^{o}C, dr > 20:1; Flash column chromatography conditions: petroleum ethers/EtOAc = 50:1; **^{1}H NMR (400 MHz, $CDCl_3$)** δ 7.23-7.17 (m, 2H), 7.12-7.08 (m, 2H), 4.65 (d, J = 12.8 Hz, 1H), 3.21 (d, J = 12.8 Hz, 1H), 2.54 (dd, J = 19.0, 0.9 Hz, 1H) , 2.44 (d, J = 18.9 Hz, 1H), 1.18 (s, 3H), 0.86 (s, 3H); **^{13}C NMR (101 MHz, $CDCl_3$)** δ 208.7, 162.3 (d, J = 246.4 Hz), 130.4 (d, J = 3.4 Hz), 130.0 (d, J = 8.0 Hz), 115.5 (d, J = 21.2 Hz), 61.8, 60.9, 51.9, 37.7, 28.2, 23.1; **^{19}F NMR (565 MHz, $CDCl_3$)** δ -114.7; **HRMS (ESI)** *m/z*: $[M + H]^+$ Calcd for $C_{13}H_{14}ClFO+H^+$: 241.0790; Found 241.0803.

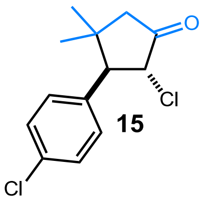

15

Compound 15: 10 h, 43 mg, 84% yield, white solid, mp 130-131 °C, dr > 20:1; Flash column chromatography conditions: petroleum ethers/EtOAc = 50:1; **^{1}H NMR (400 MHz, $CDCl_3$)** δ 7.38 (d, J = 8.4 Hz, 2H), 7.17 (d, J = 8.4 Hz, 2H), 4.65 (d, J = 12.7 Hz, 1H), 3.20 (d, J = 12.8 Hz, 1H), 2.54 (dd, J = 18.9, 0.9 Hz, 1H), 2.44 (d, J = 18.9 Hz, 1H), 1.18 (s, 3H), 0.86 (s, 3H); **^{13}C NMR (101 MHz, $CDCl_3$)** δ 208.5, 133.6, 133.2, 129.8, 128.7, 61.6, 61.1, 51.9, 37.7, 28.2, 23.1; **HRMS (ESI)** *m*/*z*: [*M* + H]$^+$ Calcd for $C_{13}H_{14}Cl_2O+H^+$: 257.0494; Found 257.0479.

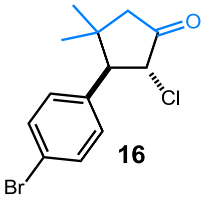

16

Compound 16: 10 h, 51 mg, 85% yield, white solid, mp 144-145 °C, dr > 20:1; Flash column chromatography conditions: petroleum ethers/EtOAc = 50:1; **^{1}H NMR (400 MHz, $CDCl_3$)** δ 7.53 (d, J = 8.0 Hz, 2H), 7.11 (d, J = 8.1 Hz, 2H), 4.65 (d, J = 12.8 Hz, 1H), 3.19 (d, J = 12.8 Hz, 1H), 2.54 (d, J = 18.9 Hz, 1H), 2.44 (d, J = 19.0 Hz, 1H), 1.17 (s, 3H), 0.86 (s, 3H); **^{13}C NMR (101 MHz, $CDCl_3$)** δ 208.4, 133.7, 131.6, 130.2, 121.7, 61.5, 61.1, 51.9, 37.6, 28.2, 23.1; **HRMS (ESI)** *m*/*z*: [*M* + Na]$^+$ Calcd for $C_{13}H_{14}BrClO+Na^+$: 322.9809; Found 322.9796.

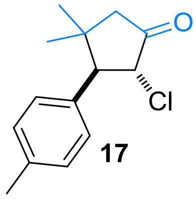

17

Compound 17: 15 h, 41 mg, 87% yield, white solid, mp 127-128 °C, dr > 20:1; Flash column chromatography conditions: petroleum ethers/EtOAc = 50:1; **^{1}H NMR (400 MHz, $CDCl_3$)** δ 7.21 (d, J = 7.9 Hz, 2H), 7.11 (d, J = 8.0 Hz, 2H), 4.69 (d, J = 12.8 Hz, 1H), 3.19 (d, J = 12.8 Hz, 1H), 2.52 (d, J = 19.0 Hz, 1H), 2.43 (d, J = 18.9 Hz, 1H), 2.37 (s, 3H), 1.17 (s, 3H), 0.86 (s, 3H); **^{13}C NMR (151 MHz, $CDCl_3$)** δ 209.3, 137.4, 131.5, 129.2, 128.4, 61.8, 61.2, 52.0, 37.6, 28.3, 23.2, 21.1; **HRMS (ESI)** *m*/*z*: [*M* + H]$^+$ Calcd for $C_{14}H_{17}ClO+H^+$: 237.1041; Found 237.1032.

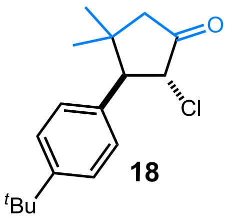

18

Compound 18: 15 h, 50 mg, 90% yield, white solid, mp 153-154 °C, dr > 20:1; Flash column chromatography conditions: petroleum ethers/EtOAc = 50:1; **^{1}H NMR (400 MHz, $CDCl_3$)** δ 7.39 (d, J = 8.2 Hz, 2H), 7.15 (d, J = 8.2 Hz, 2H), 4.71 (d, J = 12.7 Hz, 1H), 3.20 (d, J = 12.7 Hz, 1H), 2.52 (d, J = 18.9 Hz, 1H), 2.43 (d, J = 18.9 Hz, 1H), 1.33 (s, 9H), 1.19 (s, 3H), 0.86 (s, 3H); **^{13}C NMR (101 MHz, $CDCl_3$)** δ 209.4, 150.4, 131.4, 128.1, 125.3, 61.8, 61.1, 52.0, 37.7, 34.5, 31.3, 28.3, 23.2; **HRMS (ESI)** *m/z*: $[M + Na]^+$ Calcd for $C_{17}H_{23}ClO+Na^+$: 301.1330; Found 301.1317.

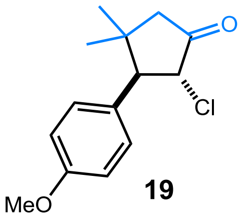

19

Compound 19: 15 h, 47 mg, 93% yield, white solid, mp 154-155 °C, dr > 20:1; Flash column chromatography conditions: petroleum ethers/EtOAc = 40:1; **^{1}H NMR (400 MHz, $CDCl_3$)** δ 7.15 (d, J = 8.6 Hz, 2H), 6.93 (d, J = 8.7 Hz, 2H), 4.66 (d, J = 12.8 Hz, 1H), 3.83 (s, 3H), 3.17 (d, J = 12.8 Hz, 1H), 2.52 (d, J = 18.9 Hz, 1H), 2.42 (d, J = 18.9 Hz, 1H), 1.17 (s, 3H), 0.86 (s, 3H); **^{13}C NMR (101 MHz, $CDCl_3$)** δ 209.3, 159.1, 129.6, 126.6, 113.9, 62.0, 60.9, 55.3, 52.0, 37.7, 28.3, 23.2; **HRMS (ESI)** *m/z*: $[M + H]^+$ Calcd for $C_{14}H_{17}ClO_2+H^+$: 253.0990; Found 253.0990.

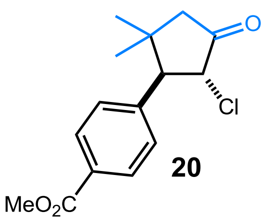

20

Compound 20: 10 h, 46 mg, 82% yield, white solid, mp 133-134 °C, dr > 20:1; Flash column chromatography conditions: petroleum ethers/EtOAc = 30:1; **^{1}H NMR (400 MHz, $CDCl_3$)** δ 8.08 (d, J = 8.1 Hz, 2H), 7.33 (d, J = 8.1 Hz, 2H), 4.75 (d, J = 12.7 Hz, 1H), 3.94 (s, 3H), 3.30 (d, J = 12.7 Hz, 1H), 2.56 (d, J = 18.9 Hz, 1H), 2.47 (d, J = 19.0 Hz, 1H), 1.20 (s, 3H), 0.87 (s, 3H); **^{13}C NMR**

(101 MHz, $CDCl_3$) δ 208.3, 166.7, 140.0, 129.7, 129.6, 128.6, 61.5, 61.3, 52.2, 51.9, 37.9, 28.2, 23.2; **HRMS (ESI)** *m*/*z*: [*M* + H]$^+$ Calcd for $C_{15}H_{17}ClO_3$+H$^+$: 281.0939; Found 281.0933.

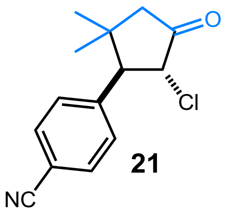

21

Compound 21: 10 h, 39 mg, 79% yield, yellow solid, mp 176-177 °C, dr > 20:1; Flash column chromatography conditions: petroleum ethers/EtOAc = 40:1; **^{1}H NMR (400 MHz, $CDCl_3$)** δ 7.72 (d, *J* = 8.1 Hz, 2H), 7.38 (d, *J* = 8.1 Hz, 2H), 4.72 (d, *J* = 12.8 Hz, 1H), 3.31 (d, *J* = 12.8 Hz, 1H), 2.58 (d, *J* = 19.0 Hz, 1H), 2.48 (d, *J* = 19.0 Hz, 1H), 1.21 (s, 3H), 0.88 (s, 3H); **^{13}C NMR (101 MHz, $CDCl_3$)** δ 207.7, 140.4, 132.3, 129.3, 118.5, 111.7, 61.5, 61.1, 51.8, 37.9, 28.2, 23.1; **HRMS (ESI)** *m*/*z*: [*M* + H]$^+$ Calcd for $C_{14}H_{14}ClNO$+H$^+$: 248.0837; Found 248.0818.

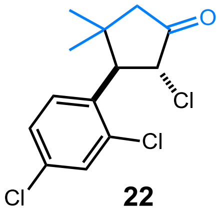

22

Compound 22: 19 h, 45 mg, 78% yield, white solid, mp 135-136 °C, dr > 20:1; Flash column chromatography conditions: petroleum ethers/EtOAc = 50:1; **^{1}H NMR (600 MHz, $CDCl_3$)** δ 7.51 (d, *J* = 2.2 Hz, 1H), 7.33-7.32 (m, 1H), 7.20 (d, *J* = 8.5 Hz, 1H), 4.57 (d, *J* = 12.4 Hz, 1H), 3.98 (d, *J* = 12.7 Hz, 1H), 2.57 (d, *J* = 19.0 Hz, 1H), 2.53 (d, *J* = 19.0 Hz, 1H), 1.21 (s, 3H), 0.97 (s, 3H); **^{13}C NMR (151 MHz, $CDCl_3$)** δ 208.1, 136.7, 133.9, 131.6, 130.1, 129.1, 127.1, 62.4, 55.7, 52.2, 38.9, 28.4, 23.6; **HRMS (ESI)** *m*/*z*: [*M* + H]$^+$ Calcd for $C_{13}H_{13}Cl_3O$+H$^+$: 291.0105; Found 291.0113.

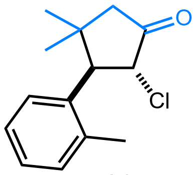

23

Compound 23: 14 h, 39 mg, 83% yield, white solid, mp 105-106 °C, dr > 20:1; Flash column chromatography conditions: petroleum ethers/EtOAc = 50:1; **^{1}H NMR (400 MHz, $CDCl_3$)** δ

7.27-7.19 (m, 4H), 4.63 (d, J = 12.5 Hz, 1H), 3.64 (d, J = 12.5 Hz, 1H), 2.57 (d, J = 18.8 Hz, 1H), 2.49 (d, J = 18.8 Hz, 1H), 2.42 (s, 3H), 1.16 (s, 3H), 1.01 (s, 3H); **^{13}C NMR (101 MHz, CDCl$_3$)** δ 209.4, 138.1, 133.4, 131.1, 127.3, 126.7, 125.8, 63.5, 55.9, 52.6, 38.9, 28.6, 23.9, 20.6; **HRMS (ESI)** m/z: $[M + Na]^+$ Calcd for $C_{14}H_{17}ClO+Na^+$: 259.0860; Found 259.0857.

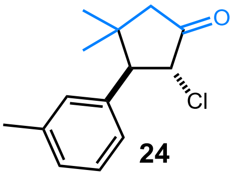

24

Compound 24: 14 h, 40 mg, 85% yield, white solid, mp 118-119 °C, dr > 20:1; Flash column chromatography conditions: petroleum ethers/EtOAc = 50:1; **^{1}H NMR (400 MHz, CDCl$_3$)** δ 7.30-7.26 (m, 1H), 7.15 (d, J = 7.5 Hz, 1H), 7.06-7.02 (m, 2H), 4.71 (d, J = 12.7 Hz, 1H), 3.19 (d, J = 12.8 Hz, 1H), 2.53 (d, J = 18.9 Hz, 1H), 2.43 (d, J = 18.9 Hz, 1H), 2.38 (s, 3H), 1.18 (s, 3H), 0.86 (s, 3H); **^{13}C NMR (101 MHz, CDCl$_3$)** δ 209.3, 138.0, 134.5, 129.4, 128.5, 128.3, 125.5, 61.8, 61.5, 52.0, 37.7, 28.3, 23.2, 21.6; **HRMS (ESI)** m/z: $[M + H]^+$ Calcd for $C_{14}H_{17}ClO+H^+$: 237.1041; Found 237.1032.

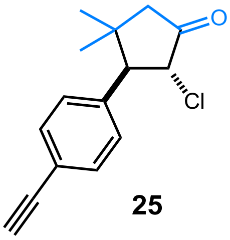

25

Compound 25: 12 h, 44 mg, 89% yield, white solid, mp 116-117 °C, dr = 10:1; Flash column chromatography conditions: petroleum ethers/EtOAc = 40:1; **^{1}H NMR (600 MHz, CDCl$_3$)** δ 7.54 (d, J = 8.2 Hz, 2H), 7.20 (d, J = 8.2 Hz, 2H), 4.69 (d, J = 12.7 Hz, 1H), 3.24 (d, J = 12.8 Hz, 1H), 3.12 (s, 1H), 2.54 (dd, J = 18.9, 1.2 Hz, 1H), 2.45 (d, J = 18.9 Hz, 1H), 1.19 (s, 3H), 0.86 (s, 3H); **^{13}C NMR (151 MHz, CDCl$_3$)** δ 208.5, 135.6, 132.2, 128.5, 121.6, 83.1, 77.8, 61.5, 61.4, 51.9, 37.9, 28.3, 23.2; **HRMS (ESI)** m/z: $[M + Na]^+$ Calcd for $C_{15}H_{15}ClO+Na^+$: 269.0704; Found 269.0710.

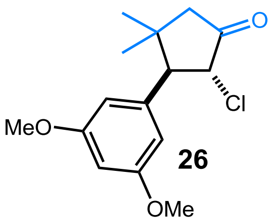

26

Compound 26: 17 h, 51 mg, 90% yield, white solid, mp 109-110 °C, dr > 20:1; Flash column chromatography conditions: petroleum ethers/EtOAc = 30:1; **^{1}H NMR (400 MHz, $CDCl_3$)** δ 6.44 (s, 1H), 6.37 (s, 2H), 4.65 (d, J = 12.7 Hz, 1H), 3.81 (s, 6H), 3.16 (d, J = 12.7 Hz, 1H), 2.52 (d, J = 18.9 Hz, 1H), 2.42 (d, J = 18.9 Hz, 1H), 1.21 (s, 3H), 0.89 (s, 3H); **^{13}C NMR (101 MHz, $CDCl_3$)** δ 209.0, 160.7, 137.0, 107.1, 98.8, 61.7, 61.7, 55.3, 52.0, 37.7, 28.5, 23.4; **HRMS (ESI)** *m*/*z*: $[M + H]^+$ Calcd for $C_{15}H_{19}ClO_3$+H^+: 283.1095; Found 283.1095.

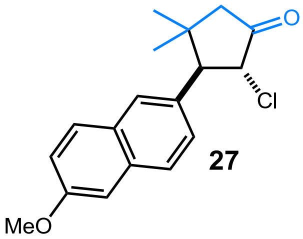

27

Compound 27: 17 h, 49 mg, 81% yield, white solid, mp 108-109 °C, dr > 20:1; Flash column chromatography conditions: petroleum ethers/EtOAc = 50:1; **^{1}H NMR (600 MHz, $CDCl_3$)** δ 7.77-7.74 (m, 2H), 7.61 (s, 1H), 7.32-7.30 (m, 1H), 7.20-7.15 (m, 2H), 4.83 (d, J = 12.7 Hz, 1H), 3.94 (s, 3H), 3.36 (d, J = 12.8 Hz, 1H), 2.57 (dd, J = 18.9, 1.2 Hz, 1H), 2.48 (d, J = 18.9 Hz, 1H), 1.23 (s, 3H), 0.90 (s, 3H); **^{13}C NMR (151 MHz, $CDCl_3$)** δ 209.2, 157.8, 134.0, 129.7, 129.3, 128.7, 127.5, 126.9, 126.7, 119.2, 105.5, 61.8, 61.6, 55.3, 52.0, 38.0, 28.4, 23.3; **HRMS (ESI)** *m*/*z*: $[M + Na]^+$ Calcd for $C_{18}H_{19}ClO_2$+Na^+: 325.0966; Found 325.0956.

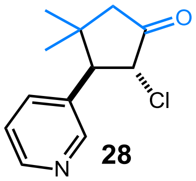

28

Compound 28: 10 h, 36 mg, 81% yield, yellow solid, mp 165-166 °C, dr > 20:1; Flash column chromatography conditions: petroleum ethers/EtOAc = 10:1; **^{1}H NMR (400 MHz, $CDCl_3$)** δ 8.61 (d, J = 4.6 Hz, 1H), 8.54 (s, 1H), 7.57 (d, J = 7.9 Hz, 1H), 7.37 (dd, J = 7.8, 4.8 Hz, 1H), 4.71 (d, J =

12.8 Hz, 1H), 3.25 (d, *J* = 12.8 Hz, 1H), 2.58 (d, *J* = 19.0 Hz, 1H), 2.48 (d, *J* = 19.0 Hz, 1H), 1.21 (s, 3H), 0.89 (s, 3H); **^{13}C NMR (101 MHz, CDCl$_3$)** δ 208.0, 150.3, 149.2, 135.4, 130.4, 123.4, 60.9, 59.3, 51.8, 37.7, 28.0, 23.1; **HRMS (ESI)** *m*/*z*: [*M* + H]$^+$ Calcd for $C_{12}H_{14}ClNO+H^+$: 224.0837; Found 224.0835.

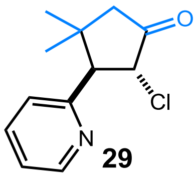
29

Compound 29: 15 h, 39 mg, 87% yield, yellow oil, dr > 20:1; Flash column chromatography conditions: petroleum ethers/EtOAc = 10:1; **^{1}H NMR (600 MHz, CDCl$_3$)** δ 8.64 (d, *J* = 4.7 Hz, 1H), 7.70-7.68 (m, 1H), 7.25-7.23 (m, 1H), 7.21 (d, *J* = 7.7 Hz, 1H), 5.35 (dd, *J* = 11.4, 1.4 Hz, 1H), 3.36 (d, *J* = 11.4 Hz, 1H), 2.54 (dd, *J* = 18.7, 1.5 Hz, 1H), 2.45 (d, *J* = 18.7 Hz, 1H), 1.26 (s, 3H), 0.80 (s, 3H); **^{13}C NMR (151 MHz, CDCl$_3$)** δ 209.8, 155.0, 149.4, 136.1, 124.7, 122.5, 62.5, 61.0, 52.2, 38.1, 28.6, 23.7; **HRMS (ESI)** *m*/*z*: [*M* + H]$^+$ Calcd for $C_{12}H_{14}ClNO+H^+$: 224.0837; Found 224.0835.

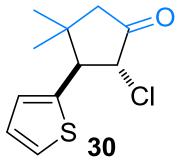
30

Compound 30: 10 h, 31 mg, 68% yield, yellow oil, dr > 20:1; Flash column chromatography conditions: petroleum ethers/EtOAc = 50:1; **^{1}H NMR (400 MHz, CDCl$_3$)** δ 7.38 (dd, *J* = 5.0, 2.9 Hz, 1H), 7.15-7.12 (m, 1H), 7.02-7.00 (m, 1H), 4.60 (dd, *J* = 12.5, 0.7 Hz, 1H), 3.32 (d, *J* = 12.5 Hz, 1H), 2.51 (dd, *J* = 18.9, 1.3 Hz, 1H) , 2.41 (d, *J* = 18.8 Hz, 1H), 1.23 (s, 3H), 0.87 (s, 3H); **^{13}C NMR (101 MHz, CDCl$_3$)** δ 208.9, 136.1, 127.2, 125.9, 122.7, 62.6, 57.3, 51.8, 37.6, 28.4, 23.4; **HRMS (ESI)** *m*/*z*: [*M* + Na]$^+$ Calcd for $C_{11}H_{13}ClOS+Na^+$: 251.0268; Found 251.0267.

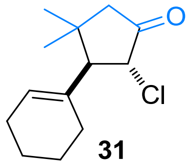
31

Compound 31: 18 h, 34 mg, 75% yield, colorless oil, dr > 20:1; Flash column chromatography

conditions: petroleum ethers/EtOAc = 50:1; **^{1}H NMR (600 MHz, CDCl$_3$)** δ 5.63-5.59 (m, 1H), 4.39 (dd, J = 12.8, 0.8 Hz, 1H), 2.54 (d, J = 12.8 Hz, 1H), 2.37 (dd, J = 18.9, 1.5 Hz, 1H), 2.28 (d, J = 18.9 Hz, 1H), 2.15-2.06 (m, 3H), 1.98-1.96 (m, 1H), 1.74-1.59 (m, 4H), 1.20 (s, 3H), 0.96 (s, 3H); **^{13}C NMR (151 MHz, CDCl$_3$)** δ 209.7, 131.2, 127.5, 62.3, 60.9, 51.9, 37.6, 29.2, 27.8, 25.3, 23.9, 22.8, 22.3; **HRMS (ESI)** m/z: $[M + H]^+$ Calcd for $C_{13}H_{19}ClO+H^+$: 227.1197; Found 227.1189.

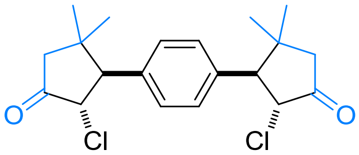

32

Compound 32: 40 h, 53 mg, 72% yield, white solid, mp 157-158 °C, dr > 20:1; Flash column chromatography conditions: petroleum ethers/EtOAc = 30:1; **^{1}H NMR (600 MHz, CDCl$_3$)** δ 7.26 (s, 4H), 4.72 (d, J = 12.6 Hz, 2H), 3.26 (d, J = 12.7 Hz, 2H), 2.55 (dd, J = 18.9, 1.1 Hz, 2H), 2.46 (d, J = 18.9 Hz, 2H), 1.22 (s, 6H), 0.87 (s, 6H); **^{13}C NMR (151 MHz, CDCl$_3$)** δ 208.9, 134.2, 128.5, 61.6, 61.2, 52.0, 51.9, 37.8, 28.4, 23.3; **HRMS (ESI)** m/z: $[M + Na]^+$ Calcd for $C_{20}H_{24}Cl_2O_2+Na^+$: 389.1046; Found 389.1016.

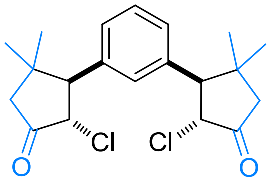

33

Compound 33: 14 h, 66 mg, 90% yield, white solid, mp 151-152 °C, dr = 10:1; Flash column chromatography conditions: petroleum ethers/EtOAc = 30:1; **^{1}H NMR (600 MHz, DMSO)** δ 7.45-7.33 (m, 2H), 7.31-7.24 (m, 2H), 5.52-5.48 (m, 2H), 3.37-3.32 (m, 6H), 1.11 (s, 3H), 1.09 (s, 3H), 0.79 (s, 3H), 0.77 (s, 3H); **^{13}C NMR (151 MHz, DMSO)** δ 209.8, 135.7, 135.7, 128.7, 128.3, 128.2, 128.2, 62.8, 62.7, 60.8, 60.7, 51.6, 51.6, 37.8, 37.7, 28.0, 28.0, 23.3, 23.2; **HRMS (ESI)** m/z: $[M + Na]^+$ Calcd for $C_{20}H_{24}Cl_2O_2+Na^+$: 389.1046; Found 389.1023.

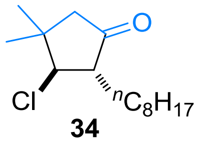

34

Compound 34: 24 h, 32 mg, 62% yield, colorless oil, dr = 5:1; Flash column chromatography conditions: petroleum ethers/EtOAc = 50:1; **^{1}H NMR (600 MHz, CDCl$_3$)** data of the *trans*-isomer δ 3.91 (d, J = 11.0 Hz, 1H), 2.29 (s, 2H), 1.97 (ddd, J = 11.0, 7.9, 4.6 Hz, 1H), 1.35-1.25 (m, 14H), 1.21 (s, 3H), 0.94 (s, 3H), 0.88 (t, J = 7.0 Hz, 3H); **^{13}C NMR (151 MHz, CDCl$_3$)** data of the *trans*-isomer δ 210.5, 64.1, 54.8, 52.6, 36.8, 31.9, 30.1, 29.4, 29.3, 29.0, 28.6, 28.5, 22.7, 22.6, 14.1; **HRMS (ESI)** *m/z*: [*M* + H]$^+$ Calcd for $C_{15}H_{27}ClO+H^+$: 259.1823; Found 259.1828.

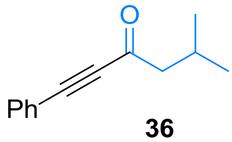

36

Compound 36*[2]*: 10 h, 9 mg, 24% yield, colorless oil; Flash column chromatography conditions: petroleum ethers/EtOAc = 20:1; **^{1}H NMR (400 MHz, CDCl$_3$)** δ 7.61-7.53 (m, 2H), 7.50-7.41 (m, 1H), 7.43-7.33 (m, 2H), 2.54 (d, J = 7.1 Hz, 2H), 2.41-2.20 (m, 1H), 1.01 (d, J = 6.7 Hz, 6H); **^{13}C NMR (101 MHz, CDCl$_3$)** δ 188.0, 133.1, 130.7, 128.6, 120.1, 90.5, 88.1, 54.5, 25.3, 22.5.

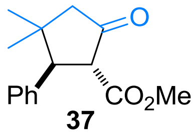

37

Compound 37: 10 h, 42 mg, 85% yield, white solid, mp 84-85 ^{o}C; The keto tautomer/enol tautomer > 20:1; Dr of the keto tautomer > 20:1; Flash column chromatography conditions: petroleum ethers/EtOAc = 30:1; **^{1}H NMR (400 MHz, CDCl$_3$)** data of the keto tautomer δ 7.36-7.26 (m, 3H), 7.22 (d, J = 7.2 Hz, 2H), 3.84 (d, J = 13.0 Hz, 1H), 3.66 (s, 3H), 3.65 (d, J = 12.6 Hz, 1H), 2.48 (d, J = 18.2 Hz, 1H), 2.42 (d, J = 18.2 Hz, 1H), 1.18 (s, 3H), 0.80 (s, 3H); **^{13}C NMR (101 MHz, CDCl$_3$)** data of the keto tautomer δ 209.9, 169.1, 136.0, 128.5, 128.3, 127.4, 58.4, 55.9, 54.5, 52.7, 38.5, 27.2, 22.9; **HRMS (ESI)** *m/z*: [*M* + Na]$^+$ Calcd for $C_{15}H_{18}O_3+Na^+$: 269.1148; Found 269.1148.

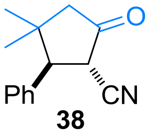

38

Compound 38: 10 h, 39 mg, 92% yield, white solid, mp 132-133 °C, dr > 20:1; Flash column chromatography conditions: petroleum ethers/EtOAc = 40:1; **^{1}H NMR (400 MHz, $CDCl_3$)** δ 7.40-7.31 (m, 3H), 7.18 (d, *J* = 7.2 Hz, 2H), 3.78 (d, *J* = 12.2 Hz, 1H), 3.04 (d, *J* = 12.2 Hz, 1H), 2.55 (d, *J* = 18.3 Hz, 1H), 2.31 (d, *J* = 18.3 Hz, 1H), 1.43 (s, 3H), 1.35 (s, 3H); **^{13}C NMR (101 MHz, $CDCl_3$)** δ 211.4, 135.0, 129.2, 128.2, 128.0, 118.3, 57.1, 52.8, 46.9, 37.0, 27.8, 24.1; **HRMS (ESI)** *m*/*z*: $[M + Na]^+$ Calcd for $C_{14}H_{15}NO+Na^+$: 236.1046; Found 236.1042.

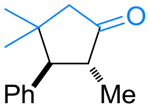

39

Compound 39: 18 h, 36 mg, 89% yield, white solid, mp 85-86 °C, dr = 1.9:1, which could be improved to > 20:1 upon combining with a subsequent epimerization using K_2CO_3 (2 equiv) in DMSO (2 mL) at 80 °C for 5 h; Flash column chromatography conditions: petroleum ethers/EtOAc = 100:1; **^{1}H NMR (400 MHz, $CDCl_3$)** δ 7.39-7.27 (m, 3H), 7.19 (d, *J* = 7.2 Hz, 2H), 2.83-2.71 (m, 2H), 2.38 (d, *J* = 18.5 Hz, 1H), 2.25 (d, *J* = 18.4 Hz, 1H), 1.13 (s, 3H), 1.03 (d, *J* = 6.1 Hz, 3H), 0.80 (s, 3H); **^{13}C NMR (101 MHz, $CDCl_3$)** δ 219.6, 137.4, 128.8, 128.2, 127.0, 60.9, 54.0, 46.5, 38.1, 28.0, 22.9, 13.1; **HRMS (ESI)** *m*/*z*: $[M + H]^+$ Calcd for $C_{14}H_{18}O+H^+$: 203.1430; Found 203.1421.

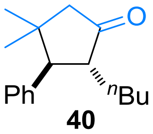

40

Compound 40: 25 h, 35 mg, 72% yield, colorless oil, dr = 2.5:1, which could be improved to > 20:1 upon combining with a subsequent epimerization using K_2CO_3 (2 equiv) in DMSO (2 mL) at 80 °C for 5 h; Flash column chromatography conditions: petroleum ethers/EtOAc = 100:1; **^{1}H NMR (400 MHz, $CDCl_3$)** δ 7.36-7.32 (m, 2H), 7.29-7.25 (m, 1H), 7.19 (d, *J* = 7.2 Hz, 2H), 2.89 (d, *J* = 12.6 Hz, 1H), 2.77-2.70 (m, 1H), 2.35 (d, *J* = 18.8 Hz, 1H), 2.23 (d, *J* = 18.0 Hz, 1H), 1.59-1.43 (m, 2H), 1.28-1.06 (m, 7H), 0.80-0.73 (m, 6H); **^{13}C NMR (151 MHz, $CDCl_3$)** δ 219.7, 137.9, 128.8, 128.0, 126.8, 58.4, 54.8, 51.0, 38.0, 28.9, 28.8, 27.8, 22.9, 22.8, 13.8; **HRMS (ESI)** *m*/*z*: $[M + H]^+$ Calcd

for $C_{17}H_{24}O+H^+$: 245.1900; Found 245.1895.

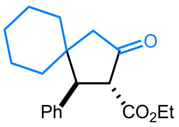

42

Compound 42: 13 h, 45 mg, 75% yield, colorless oil; The keto tautomer/enol tautomer = 10:1; Dr of the keto tautomer > 20:1; Flash column chromatography conditions: petroleum ethers/EtOAc = 30:1; **^{1}H NMR (400 MHz, CDCl$_3$)** data of the keto tautomer δ 7.37-7.26 (m, 3H), 7.22-7.16 (m, 2H), 4.16-4.06 (m, 2H), 3.83 (d, J = 13.2 Hz, 1H), 3.56 (d, J = 13.2 Hz, 1H), 2.82 (dd, J = 18.4, 0.8 Hz, 1H), 2.23 (dd, J = 18.4, 2.0 Hz, 1H), 1.69-1.50 (m, 5H), 1.36-1.27 (m, 3H), 1.16 (t, J = 7.1 Hz, 3H), 1.05-0.79 (m, 2H); **^{13}C NMR (101 MHz, CDCl$_3$)** data of the keto tautomer δ 210.0, 168.7, 135.7, 129.0, 128.0, 127.2, 61.3, 58.0, 56.9, 48.9, 42.1, 36.9, 29.8, 25.5, 23.8, 22.0, 14.0; **HRMS (ESI)** *m*/*z*: $[M + H]^+$ Calcd for $C_{19}H_{24}O_3+H^+$: 301.1798; Found 301.1784.

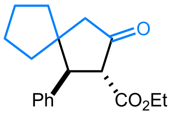

43

Compound 43: 15 h, 53 mg, 93% yield, colorless oil; The keto tautomer/enol tautomer = 10:1; Dr of the keto tautomer > 20:1; Flash column chromatography conditions: petroleum ethers/EtOAc = 30:1; **^{1}H NMR (400 MHz, CDCl$_3$)** data of the keto tautomer δ 7.36-7.33 (m, 2H), 7.30-7.26 (m, 1H), 7.24-7.22 (m, 2H), 4.15-4.05 (m, 2H), 3.86 (d, J = 13.0 Hz, 1H), 3.76 (d, J = 13.0 Hz, 1H), 2.57 (d, J = 18.2 Hz, 1H), 2.35 (d, J = 18.2 Hz, 1H), 1.78-1.72 (m, 1H), 1.58-1.40 (m, 5H), 1.34-1.26 (m, 2H), 1.18 (t, J = 7.1 Hz, 3H); **^{13}C NMR (101 MHz, CDCl$_3$)** data of the keto tautomer δ 209.9, 168.6, 136.2, 129.0, 128.2, 127.3, 61.4, 59.8, 53.2, 52.5, 49.9, 36.1, 31.4, 23.2, 23.1, 14.1; **HRMS (ESI)** *m*/*z*: $[M + Na]^+$ Calcd for $C_{18}H_{22}O_3+Na^+$: 309.1461; Found 309.1455.

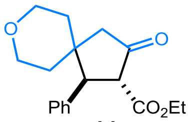

44

Compound 44: 13 h, 44 mg, 73% yield, colorless oil; The keto tautomer/enol tautomer = 3.3:1; Dr of the keto tautomer > 20:1; Flash column chromatography conditions: petroleum ethers/EtOAc =

30:1; **^{1}H NMR (600 MHz, $CDCl_3$)** data of the keto tautomer *δ* 7.37-7.34 (m, 2H), 7.32-7.29 (m, 1H), 7.20-7.19 (m, 2H), 4.16-4.06 (m, 2H), 3.87-3.81 (m, 3H), 3.60 (d, *J* = 13.2 Hz, 1H), 3.47-3.42 (m, 1H), 3.40-3.36 (m, 1H), 2.95 (d, *J* = 18.3 Hz, 1H), 2.32 (dd, *J* = 18.3, 1.9 Hz, 1H), 1.95-1.90 (m, 1H), 1.51-1.45 (m, 1H), 1.43-1.40 (m, 1H), 1.18 (t, *J* = 7.1 Hz, 3H), 1.14-1.11 (m, 1H); **^{13}C NMR (151 MHz, $CDCl_3$)** data of the keto tautomer *δ* 208.9, 168.4, 135.0, 129.1, 128.4, 127.7, 65.4, 63.9, 61.6, 57.8, 56.4, 48.2, 40.0, 36.2, 30.3, 14.1; **HRMS (ESI)** *m*/*z*: $[M + H]^+$ Calcd for $C_{18}H_{22}O_4+H^+$: 303.1591; Found 303.1581.

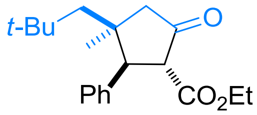

45

Compound 45: 10 h, 57 mg, 90% yield, colorless oil; The keto tautomer/enol tautomer > 20:1; Dr of the keto tautomer > 20:1; Flash column chromatography conditions: petroleum ethers/EtOAc = 40:1; **^{1}H NMR (400 MHz, $CDCl_3$)** data of the keto tautomer *δ* 7.37-7.23 (m, 5H), 4.15-4.01 (m, 2H), 3.71 (d, *J* = 12.9 Hz, 1H), 3.53 (d, *J* = 12.9 Hz, 1H) 2.63 (d, *J* = 17.9 Hz, 1H), 2.53 (d, *J* = 17.8 Hz, 1H), 1.62 (d, *J* = 14.3 Hz, 1H), 1.48 (d, *J* = 14.3 Hz, 1H), 1.16 (t, *J* = 7.1 Hz, 3H), 0.96 (s, 3H), 0.93 (s, 9H); **^{13}C NMR (101 MHz, $CDCl_3$)** data of the keto tautomer *δ* 210.7, 168.7, 136.1, 129.1, 128.1, 127.3, 61.5, 58.2, 57.3, 54.1, 54.0, 42.3, 32.0, 31.7, 21.3, 14.0; **HRMS (ESI)** *m*/*z*: $[M + H]^+$ Calcd for $C_{20}H_{28}O_3+H^+$: 317.2111; Found 317.2100; The regio- and diastereoselectivity was determined by 2D NOESY measurements.

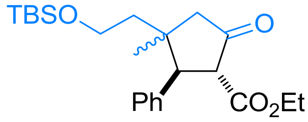

46

Compound 46: 10 h, 61 mg, 75% yield, colorless oil; The keto tautomer/enol tautomer > 20:1; Dr of the keto tautomer = 1.2:1; Flash column chromatography conditions: petroleum ethers/EtOAc = 100:1; **^{1}H NMR (600 MHz, $CDCl_3$)** data of the major keto tautomer *δ* 7.37-7.34 (m, 2H), 7.31-7.30 (m, 1H), 7.26-7.24 (m, 2H), 4.17-4.07 (m, 2H), 3.79 (d, *J* = 12.6 Hz, 1H), 3.75-3.64 (m, 2H), 3.59-3.54 (m, 1H), 2.63 (d, *J* = 18.3 Hz, 1H), 2.50 (dd, *J* = 18.3, 0.9 Hz, 1H), 1.78-1.68 (m, 1H), 1.20 (t, *J* = 7.1, 3H), 0.90 (s, 9H), 0.06 (s, 3H), 0.05 (s, 3H); **^{13}C NMR (151 MHz, $CDCl_3$)** data of

the major keto tautomer δ 210.2, 168.7, 136.2, 128.8, 128.2, 127.4, 61.4, 59.7, 57.8, 55.9, 53.0, 42.4, 40.5, 25.9, 18.2, 14.1, -5.4, -5.5; **HRMS (ESI)** *m*/*z*: [*M* + H]$^+$ Calcd for $C_{23}H_{36}O_4Si$+H$^+$: 405.2456; Found 405.2437.

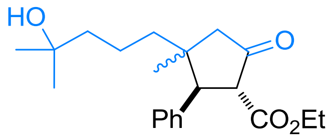

47

Compound 47: 11 h, 61 mg, 88% yield, colorless oil; The keto tautomer/enol tautomer = 7:1; Dr of the keto tautomer = 1:1; Flash column chromatography conditions: petroleum ethers/EtOAc = 10:1; **^{1}H NMR (600 MHz, CDCl$_3$)** data of the keto tautomer δ 7.35-7.32 (m, 2H), 7.30-7.28 (m, 1H), 7.24-7.20 (m, 2H), 4.12-4.08 (m, 2H), 3.86 (d, *J* = 13.3 Hz, 1H), 3.71 (d, *J* = 13.0 Hz, 1H), 2.46 (d, *J* = 18.0 Hz, 1H), 2.40 (d, *J* = 17.9 Hz, 1H), 1.49-1.28 (m, 6H), 1.25-0.83 (m, 13H); **^{13}C NMR (151 MHz, CDCl$_3$)** data of the keto tautomer δ 209.9, 168.7, 136.2, 128.7, 128.3, 127.3, 70.8, 58.4, 57.3, 55.5, 51.0, 44.4, 41.7, 40.9, 34.6, 32.4, 29.3, 18.9, 14.1; **HRMS (ESI)** *m*/*z*: [*M* - H]$^+$ Calcd for $C_{21}H_{30}O_4$-H$^+$: 345.2071; Found 345.2073.

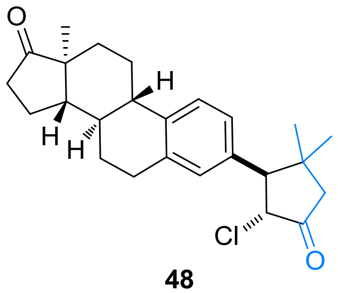

48

Compound 48: 13 h, 64 mg, 80% yield, white solid, mp 255-256 ^{o}C, dr > 20:1; Flash column chromatography conditions: petroleum ethers/EtOAc = 50:1; **^{1}H NMR (400 MHz, CDCl$_3$)** δ 7.30 (d, *J* = 8.1 Hz, 1H), 7.00 (d, *J* = 7.9 Hz, 1H), 6.93 (s, 1H), 4.69 (d, *J* = 12.7 Hz, 1H), 3.16 (d, *J* = 12.8 Hz, 1H), 2.98-2.90 (m, 2H), 2.55-2.40 (m, 4H), 2.36-2.27 (m, 1H), 2.21-1.99 (m, 4H), 1.65-1.45 (m, 6H), 1.19 (s, 3H), 0.93 (s, 3H), 0.87 (s, 3H); **^{13}C NMR (101 MHz, CDCl$_3$)** δ 220.9, 209.3, 139.2, 136.5, 131.9, 129.2 (d, *J* = 6.9 Hz), 125.8 (d, *J* = 10.2 Hz), 125.4, 61.8 (d, *J* = 3.5 Hz), 61.2 (d, *J* = 3.6 Hz), 52.1, 50.5, 48.0, 44.4, 38.0, 37.7, 35.9, 31.6, 29.5 (d, *J* = 3.5 Hz), 28.4, 26.5, 25.6, 23.3, 21.6, 13.9; **HRMS (ESI)** *m*/*z*: [*M* + H]$^+$ Calcd for $C_{25}H_{31}ClO_2$+H$^+$: 399.2085; Found 399.2058.

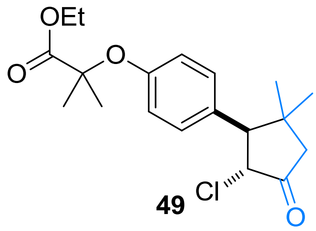

49

Compound 49: 15 h, 64 mg, 91% yield, white solid, mp 103-104 °C, dr > 20:1; Flash column chromatography conditions: petroleum ethers/EtOAc = 30:1; **^{1}H NMR (400 MHz, CDCl$_3$)** δ 7.09 (d, J = 8.6 Hz, 2H), 6.86 (d, J = 8.6 Hz, 2H), 4.65 (d, J = 12.8 Hz, 1H), 4.25 (q, J = 7.1 Hz, 2H), 3.16 (d, J = 12.8 Hz, 1H), 2.51 (d, J = 18.9 Hz, 1H), 2.42 (d, J = 18.9 Hz, 1H), 1.62 (s, 6H), 1.24 (t, J = 7.1 Hz, 3H), 1.15 (s, 3H), 0.83 (s, 3H); **^{13}C NMR (101 MHz, CDCl$_3$)** δ 209.2, 174.2, 155.0, 129.1, 127.9, 118.6, 79.1, 61.8, 61.4, 60.8, 51.9, 37.6, 28.2, 25.4, 25.4, 23.1, 14.0; **HRMS (ESI)** *m*/*z*: [*M* + H]$^+$ Calcd for $C_{19}H_{25}ClO_4$+H$^+$: 353.1514; Found 353.1501.

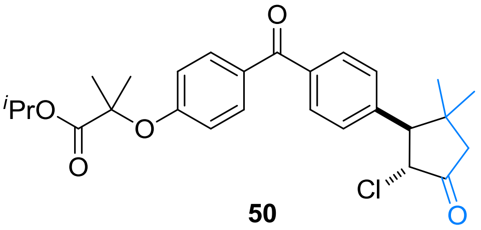

50

Compound 50: 17 h, 82 mg, 87% yield, yellow solid, mp 125-126 °C, dr > 20:1; Flash column chromatography conditions: petroleum ethers/EtOAc = 30:1; **^{1}H NMR (400 MHz, CDCl$_3$)** δ 7.81-7.78 (m, 4H), 7.34 (d, J = 8.2 Hz, 2H), 6.90-6.86 (m, 2H), 5.14-5.15 (m, 1H), 4.75 (d, J = 12.7 Hz, 1H), 3.32 (d, J = 12.7 Hz, 1H), 2.57 (dd, J = 18.9, 1.2 Hz, 1H), 2.48 (d, J = 18.9 Hz, 1H), 1.67 (s, 6H), 1.23-1.19 (m, 9H), 0.90 (s, 3H); **^{13}C NMR (101 MHz, CDCl$_3$)** δ 208.4, 195.0, 173.1, 159.7, 139.0, 137.6, 132.1, 130.4, 129.9, 128.5, 117.2, 79.4, 69.4, 61.6, 61.4, 52.0, 37.9, 28.3, 25.4, 23.2, 21.5; **HRMS (ESI)** *m*/*z*: [*M* + H]$^+$ Calcd for $C_{27}H_{31}ClO_5$+H$^+$: 471.1933; Found 471.1907.

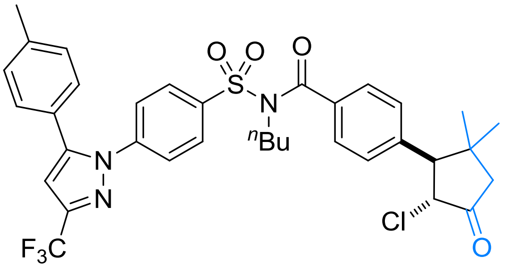

51

Compound 51: 10 h, 112 mg, 82% yield, yellow solid, mp 257-258 °C, dr > 20:1; Flash column chromatography conditions: petroleum ethers/EtOAc = 10:1; **^{1}H NMR (400 MHz, CDCl$_3$)** δ 7.85-7.80 (m, 2H), 7.50-7.44 (m, 4H), 7.24 (d, J = 8.2 Hz, 2H), 7.18 (d, J = 7.9 Hz, 2H), 7.10 (d, J = 8.1 Hz, 2H), 6.75 (s, 1H), 4.69 (d, J = 12.7 Hz, 1H), 3.84-3.79 (m, 2H), 3.26 (d, J = 12.7 Hz, 1H), 2.55 (dd, J = 18.9, 0.9 Hz, 1H), 2.46 (d, J = 19.0 Hz, 1H), 2.38 (s, 3H), 1.68-1.61 (m, 2H), 1.29-1.23 (m, 2H), 1.18 (s, 3H), 0.85 (s, 3H), 0.83 (t, J = 7.4 Hz, 3H); **^{13}C NMR (101 MHz, CDCl$_3$)** δ 208.2, 171.0, 145.3, 144.3 (q, J = 38.3 Hz), 143.2, 139.9, 138.9, 138.2, 134.2, 129.8, 129.5, 128.8, 128.5, 128.4, 125.7, 125.2, 121.0 (q, J = 274.0 Hz), 106.5, 63.7, 61.4 (q, J = 11.9 Hz), 51.9, 48.0, 37.9, 31.7, 28.2, 23.2, 21.4, 19.8, 13.5; **^{19}F NMR (377 MHz, CDCl$_3$)** δ -62.5; **HRMS (ESI)** *m*/*z*: $[M + H]^+$ Calcd for $C_{35}H_{35}ClF_3N_3O_4S+H^+$: 686.2062; Found 686.2030.

1.3 Experimental procedure for the transformation of 17 to 52

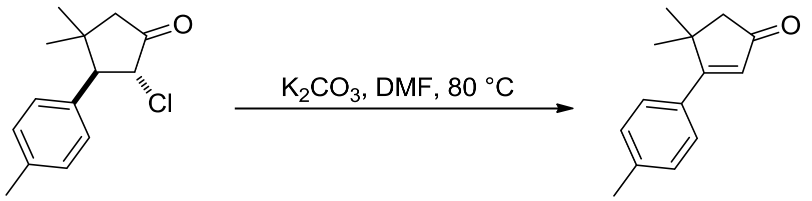

17 **52**

To a solution of **17** (47.2 mg, 0.2 mmol) in 2 mL of DMF was added K_2CO_3 (55.2 mg, 0.4 mmol). After stirring at 80 ℃ for 4 h, the reaction mixture was quenched with water, extracted with EtOAc, washed with brine, dried over anhydrous $MgSO_4$, and concentrated. Column chromatography on silica gel (petroleum ethers/EtOAc = 20:1) gave 37 mg (93% yield) of **52**[3] as a colorless oil; **^{1}H NMR (400 MHz, CDCl$_3$)** δ 7.43-7.40 (m, 2H), 7.23 (d, J = 8.0 Hz, 2H), 6.18 (s, 1H), 2.49 (s, 2H), 2.40 (s, 3H), 1.43 (s, 6H); **^{13}C NMR (101 MHz, CDCl$_3$)** δ 207.5, 183.5, 140.0, 131.7, 129.2, 128.7, 127.8, 53.7, 43.6, 28.1, 21.3.

1.4 Experimental procedure for the transformation of 52 to 53

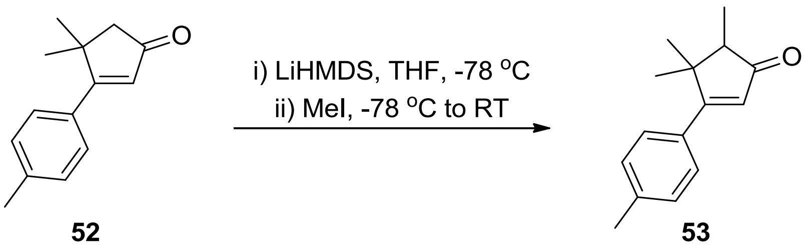

52 **53**

To a solution of **52** (50.8 mg, 0.2 mmol) in 1 mL of dry THF was added LiHMDS (0.24 mL, 1.0 M solution in THF) at -78 ℃. After stirring at -78 ℃ for 1 h, MeI (42.6 mg, 0.3 mmol) was added, then warmed to 25 °C over 2 h. The reaction mixture was quenched with saturated NH_4Cl solution, extracted with EtOAc, dried over anhydrous $MgSO_4$, and concentrated. Column chromatography on silica gel (petroleum ethers/EtOAc = 50:1) gave 36 mg (84% yield) of **53**[4] as a colorless oil; **^{1}H NMR (600 MHz, $CDCl_3$)** δ 7.39 (d, J = 8.2 Hz, 2H), 7.23 (d, J = 7.9 Hz, 2H), 6.16 (s, 1H), 2.40 (s, 3H), 2.37 (q, J = 7.32 Hz, 1H), 1.35 (s, 3H), 1.23 (s, 3H), 1.15 (d, J = 7.4 Hz, 3H); **^{13}C NMR (151 MHz, $CDCl_3$)** δ 209.7, 182.4, 139.7, 132.1, 129.2, 127.8, 127.8, 54.6, 46.7, 26.4, 24.7, 21.3, 9.8.

1.5 Experimental procedure for the transformation of 52 to 55

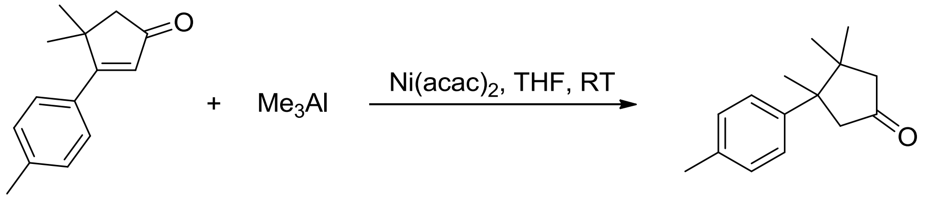

52 **55**

To a solution of **52** (40.0 mg, 0.2 mmol) and Ni(acac)$_2$ (5.1 mg, 0.02 mmol) in 2 mL of THF was added Me_3Al (0.2 mL, 1.0 M solution in THF) at 0 °C. After stirring at 25 °C for 2 h, the reaction mixture was quenched with saturated NH_4Cl solution, extracted with EtOAc, washed with brine, dried over anhydrous Na_2SO_4, and concentrated. Column chromatography on silica gel (petroleum ethers/EtOAc = 50:1) gave 36 mg (83% yield) of **55**[5] as a colorless oil; **^{1}H NMR (600 MHz, $CDCl_3$)** δ 7.24-7.21 (m, 2H), 7.16 (d, J = 8.0 Hz, 2H), 3.15 (d, J = 18.3 Hz, 1H), 2.36 (t, J = 9.5 Hz, 5H), 2.26 (dd, J = 19.5, 1.2 Hz, 1H), 1.44 (s, 3H), 1.25 (s, 3H), 0.75 (s, 3H); **^{13}C NMR (151 MHz, $CDCl_3$)** δ 218.3, 141.2, 135.8, 128.7, 126.5, 52.4, 50.7, 47.8, 41.8, 26.2, 24.4, 24.1, 20.8.

1.6 Mechanistic experiments

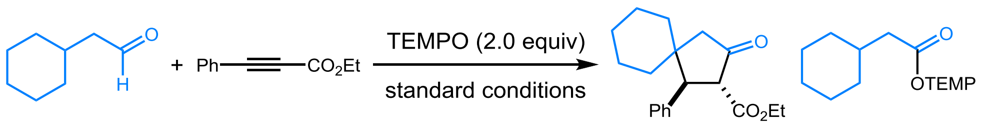

1b **2a** **42**, trace **57**, 38%

To a mixture of TBADT (10.6 mg, 0.004 mmol) in 2 mL of MeCN/H_2O (v:v = 10:1) was added **1b** (50.4 mg, 0.4 mmol), **2a** (34.8 mg, 0.2 mmol), and TEMPO (62.5 mg, 0.4 mmol) under nitrogen atmosphere. After 10 h of irradiation with purple LEDs (100% intensity, Kessil PR160, 40 W, 390

nm, light irradiance at a distance of 3 cm: 76.41 mW/cm^2, and the light irradiance at the reaction site: 4.62 mW/cm^2), the reaction mixture was quenched with water, extracted with EtOAc, washed with brine, dried over anhydrous Na_2SO_4, and concentrated. Column chromatography on silica gel (petroleum ethers/EtOAc = 5:1) gave 43 mg (38% yield) of **57** as a colorless oil; **^{1}H NMR (600 MHz, $CDCl_3$)** δ 2.15 (d, J = 7.0 Hz, 2H), 1.85-1.61 (m, 12H), 1.33 (s, 12H), 1.29-1.24 (m, 2H), 1.16-1.10 (m, 1H), 1.01-0.93 (m, 2H); **^{13}C NMR (151 MHz, $CDCl_3$)** δ 180.4, 65.1, 44.5, 37.2, 35.6, 33.3, 27.7, 26.4, 26.2, 20.3, 16.0; **HRMS (ESI)** m/z: $[M + H]^+$ Calcd for $C_{17}H_{31}NO_2+H^+$: 282.2428; Found 282.2428.

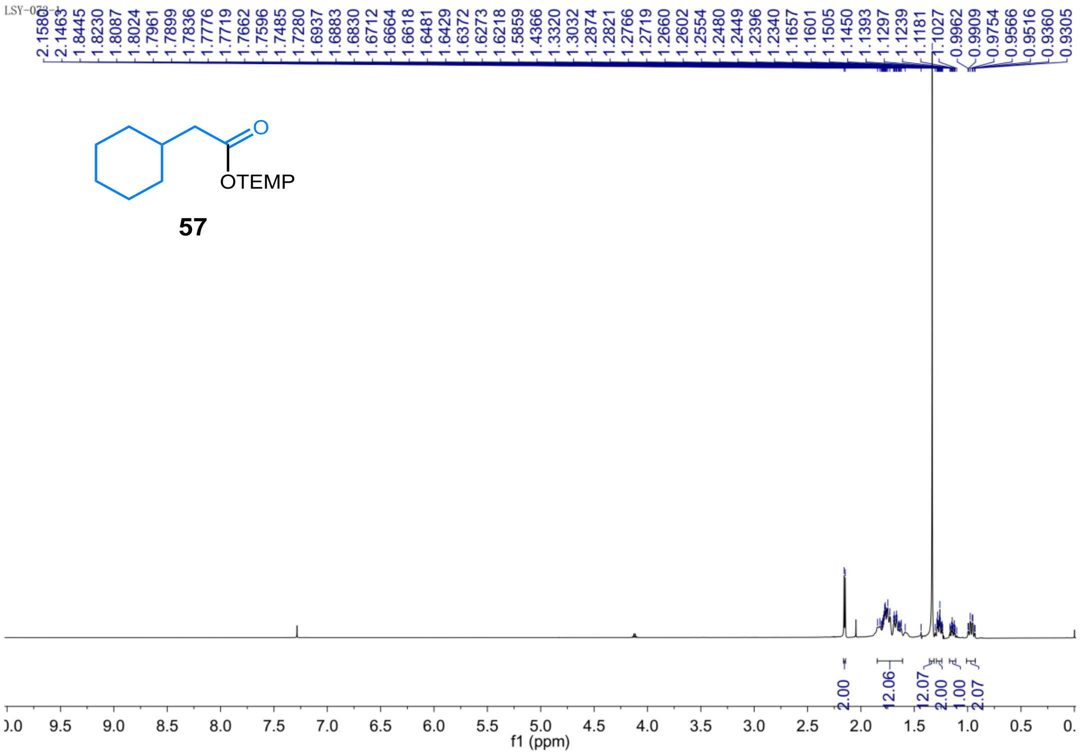

Supplementary Fig. 3. ^{1}H NMR spectrum (400 MHz, $CDCl_3$, 298K) of **57.**

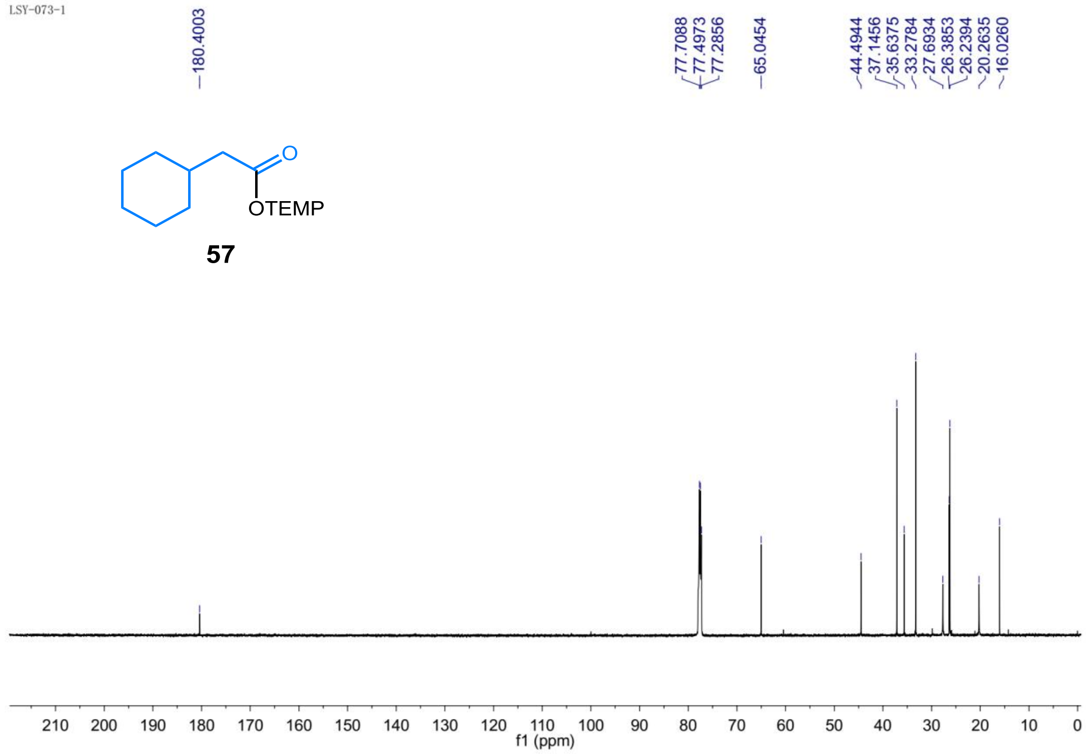

Supplementary Fig. 4. ^{13}C NMR spectrum (151 MHz, $CDCl_3$, 298K) of **57.**

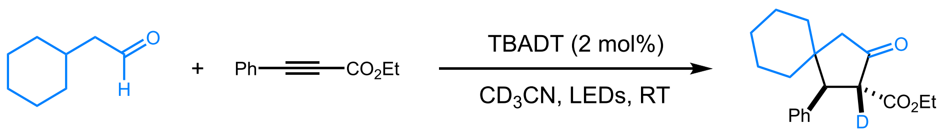

1b **2a** [D]-**42**, <2% D

To a mixture of TBADT (10.6 mg, 0.004 mmol) in 2 mL of CD_3CN (> 99% D) was added **1b** (50.4 mg, 0.4 mmol) and **2a** (34.8 mg, 0.2 mmol) under nitrogen atmosphere. After 10 h of irradiation with purple LEDs (100% intensity, Kessil PR160, 40 W, 390 nm, light irradiance at a distance of 3 cm: 76.41 mW/cm^2, and the light irradiance at the reaction site: 4.62 mW/cm^2), the reaction mixture was directly monitored by GC analysis (Due to the formation of an enol tautomer, the D/H exchange of [D]-**42** may occur upon chromatography on silica gel, as such, the isolated yield could not be obtained) and ^{1}H NMR spectroscopy, indicating that [D]-**42** was formed in 67% GC yield with <2% deuterium incorporation.

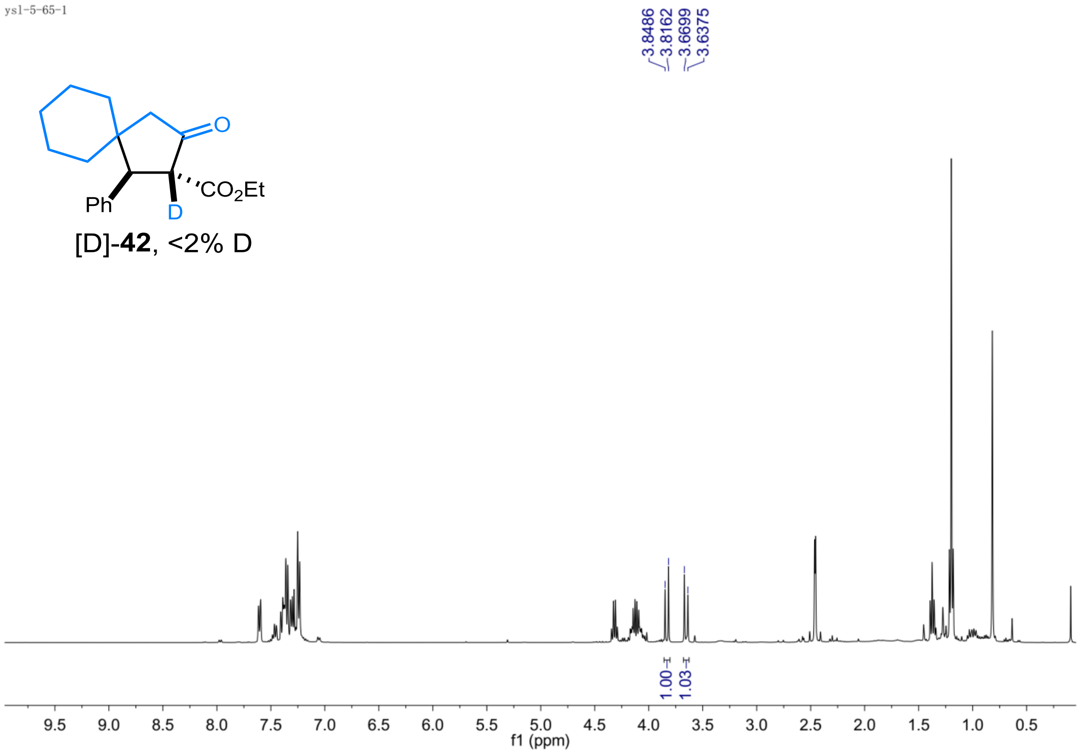

Supplementary Fig. 5. ^{1}H NMR spectrum (600 MHz, $CDCl_3$, 298K) of [D]-**42.**

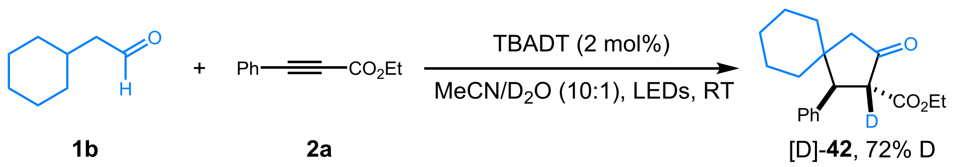

To a mixture of TBADT (10.6 mg, 0.004 mmol) in 2 mL of 10:1 mixture of MeCN and D_2O (> 99% D) was added **1b** (50.8 mg, 0.4 mmol) and **2a** (34.8 mg, 0.2 mmol) under nitrogen atmosphere. After 10 h of irradiation with purple LEDs (100% intensity, Kessil PR160, 40 W, 390 nm, light irradiance at a distance of 3 cm: 76.41 mW/cm^2, and the light irradiance at the reaction site: 4.62 mW/cm^2), the reaction mixture was directly monitored by GC analysis and ^{1}H NMR spectroscopy, indicating that [D]-**42** was formed in 90% GC yield with 72% deuterium incorporation.

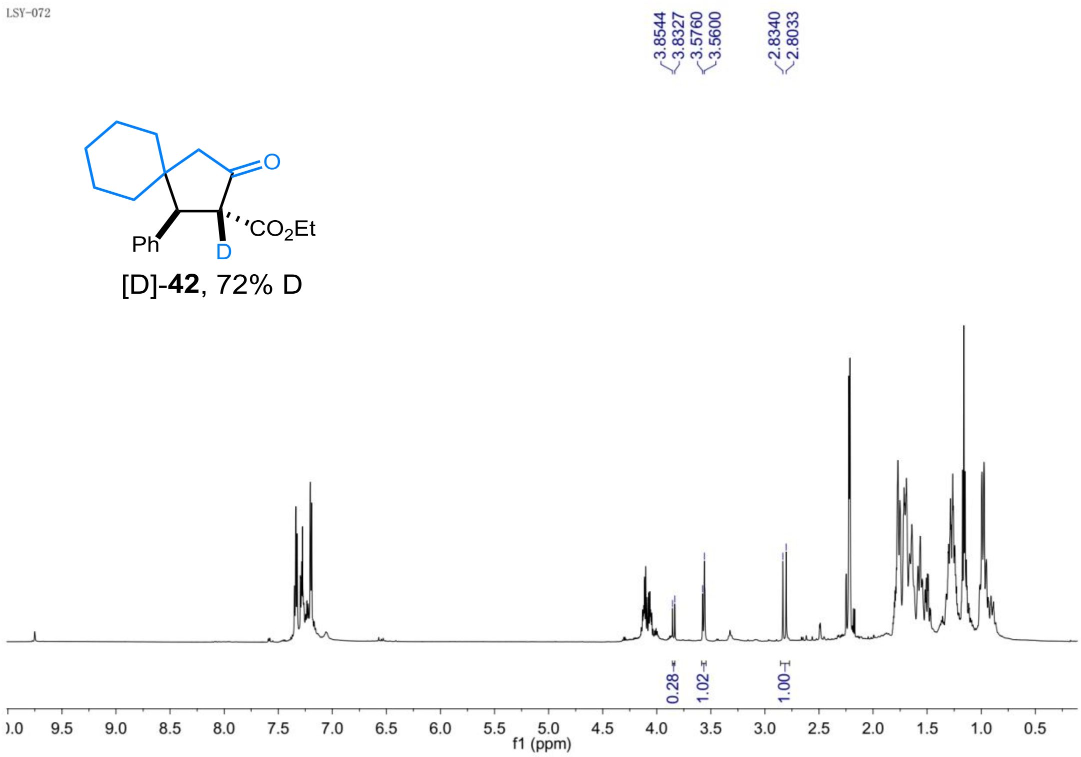

Supplementary Fig. 6. ^{1}H NMR spectrum (600 MHz, $CDCl_3$, 298 K) of [D]-**42.**

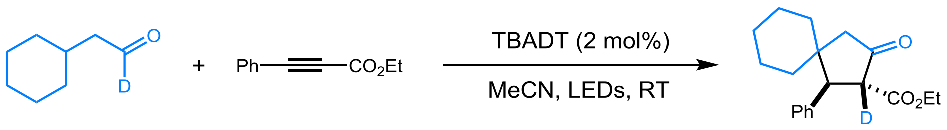

[D]-**1b**, 98% D **2a** [D]-**42**, 60% D

To a mixture of TBADT (10.6 mg, 0.004 mmol) in 400 μL of MeCN was added [D]-**1b**[6] (98% D, 50.8 mg, 0.4 mmol) and **2a** (34.8 mg, 0.2 mmol) under nitrogen atmosphere. After 3 h of irradiation with purple LEDs (100% intensity, Kessil PR160, 40 W, 390 nm, light irradiance at a distance of 3 cm: 76.41 mW/cm^2, and the light irradiance at the reaction site: 4.62 mW/cm^2), the reaction mixture was directly monitored by GC analysis and ^{1}H NMR spectroscopy, indicating that [D]-**42** was formed in 37% GC yield with 60% deuterium incorporation.

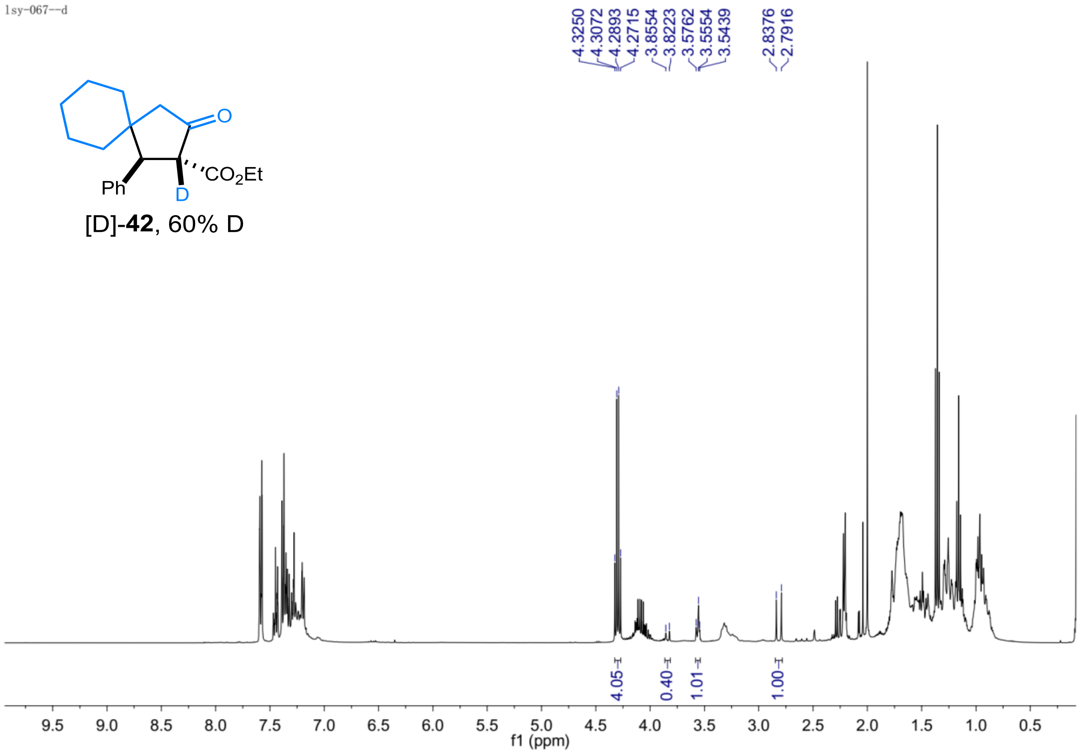

Supplementary Fig. 7. ^{1}H NMR spectrum (600 MHz, $CDCl_3$, 298K) of [D]-**42.**

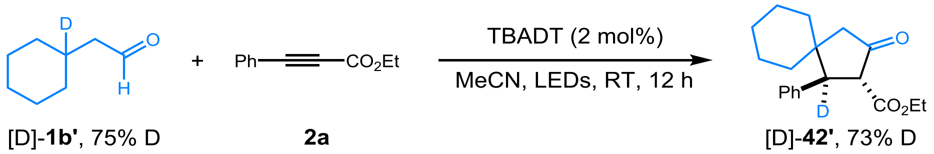

To a mixture of TBADT (10.6 mg, 0.004 mmol) in 2 mL of MeCN was added [D]-**1b'**[7] (75% D, 50.8 mg, 0.4 mmol) and **2a** (34.8 mg, 0.2 mmol) under nitrogen atmosphere. After 16 h of irradiation with purple LEDs (100% intensity, Kessil PR160, 40 W, 390 nm, light irradiance at a distance of 3 cm: 76.41 mW/cm^2, and the light irradiance at the reaction site: 4.62 mW/cm^2), the reaction mixture was quenched with water, extracted with EtOAc, washed with brine, dried over anhydrous Na_2SO_4, and concentrated. Column chromatography on silica gel (petroleum ethers/EtOAc = 40:1) gave 37 mg (61% yield) of [D]-**42'** with 73% deuterium incorporation as a colorless oil; **^{1}H NMR (600 MHz, $CDCl_3$)** δ 7.35-7.32 (m, 2H), 7.30-7.27 (m, 1H), 7.21-7.18 (m, 2H), 4.14-4.04 (m, 2H), 3.82 (s, 1H), 2.82 (dd, J = 18.4, 0.9 Hz, 1H), 2.23 (dd, J = 18.5, 2.0 Hz, 1H), 1.64-1.54 (m, 4H), 1.51-1.46 (m, 1H), 1.32-1.20 (m, 3H), 1.16 (t, J = 7.1 Hz, 3H), 1.00-0.86 (m, 2H); **^{13}C NMR (151 MHz, $CDCl_3$)** δ 210.0, 168.8, 135.8, 129.1, 128.1, 127.3, 61.4, 58.1, 56.6, 49.0, 42.2, 36.9, 29.9, 25.6, 23.9, 22.1, 14.1; **HRMS (ESI)** m/z: $[M + Na]^+$ Calcd for $C_{19}H_{23}DO_3$+Na^+: 324.1680; Found 324.1669.

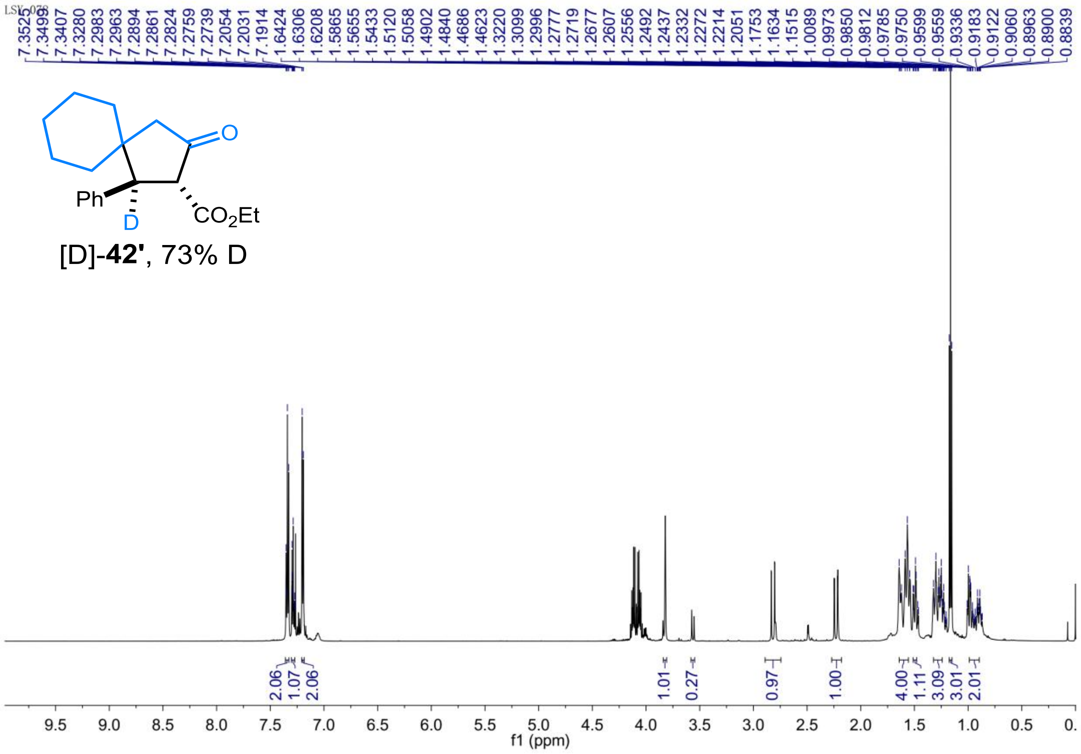

Supplementary Fig. 8. ^{1}H NMR spectrum (600 MHz, $CDCl_3$, 298K) of [D]-**37'.**

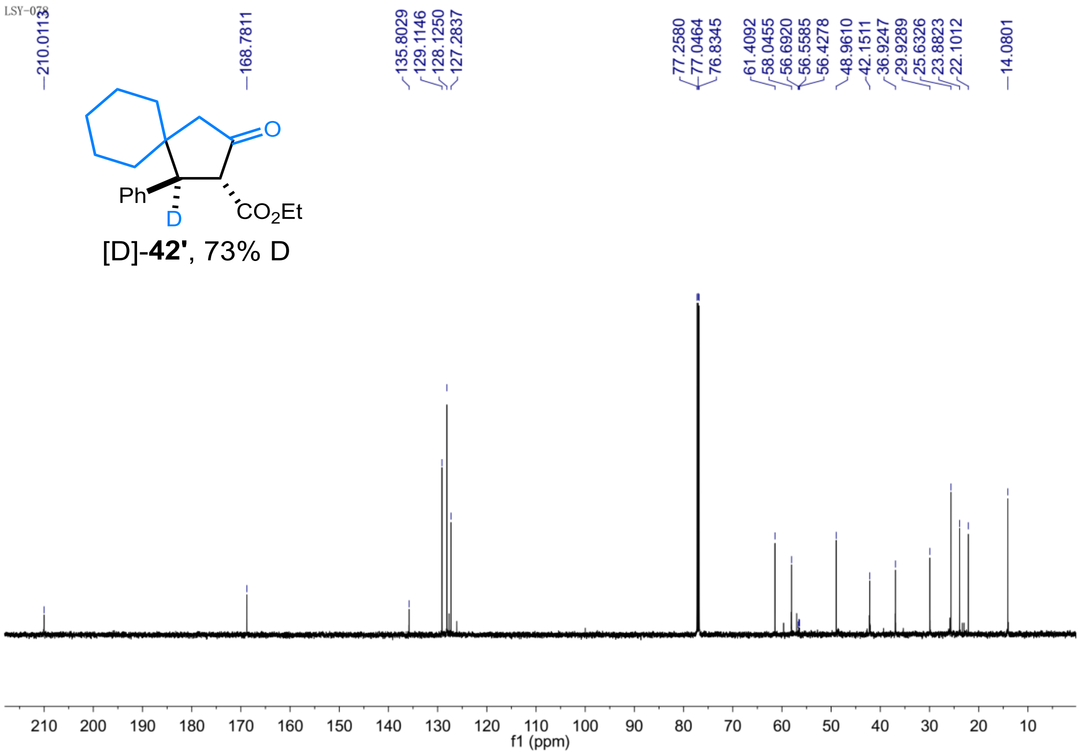

Supplementary Fig. 9. ^{13}C NMR spectrum (151 MHz, $CDCl_3$, 298K) of [D]-**42'.**

1.7 Crystallographic data

The Crystallographic data have been deposited with the Cambridge Crystallographic Data Center as CCDC 2164582 (**6**) and 2164583 (**13**).

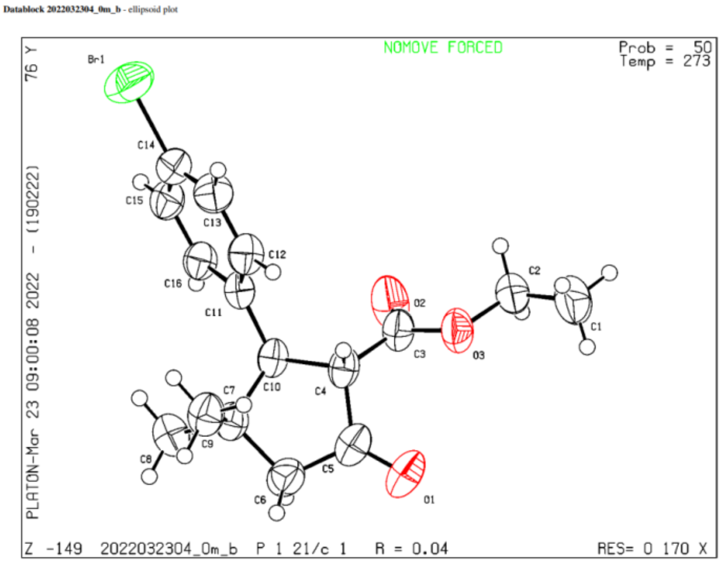

Supplementary Fig. 10. Crystal structure of **6** (CCDC **2164582**).

Supplementary Table 1. Crystal data for product **6**.

Empirical formula	$C_{16}H_{19}BrO_3$
Formula weight	338.05
Temperature	297(2) K
Crystal system, space group	Monoclinic, P2(1) / c
Unit cell dimensions	*a* = 12.1015(19) Å alpha = 90 deg.
	b = 9.7752(13) Å beta = 92.403(4) deg.
	c = 22.586(4) Å gamma = 90 deg.
Volume	2669.5(7) $Å^3$
Z	8
Absorption coefficient	0.120 mm^{-1}
Reflections collected	6094
Independent reflections	4325 [*R*(int) = 0.0398]
Refinement method	by full-matrix least-squares on F^2
Data / restraints / parameters	4325 / 0 / 365

Goodness-of-fit on F^2	1.042
Final R indices [$I > 2s(I)$]	$wR_1 = 0.0685$, $wR_2 = 0.2334$
Largest diff peak and hole	0.493 and -0.462 e.Å^{-3}

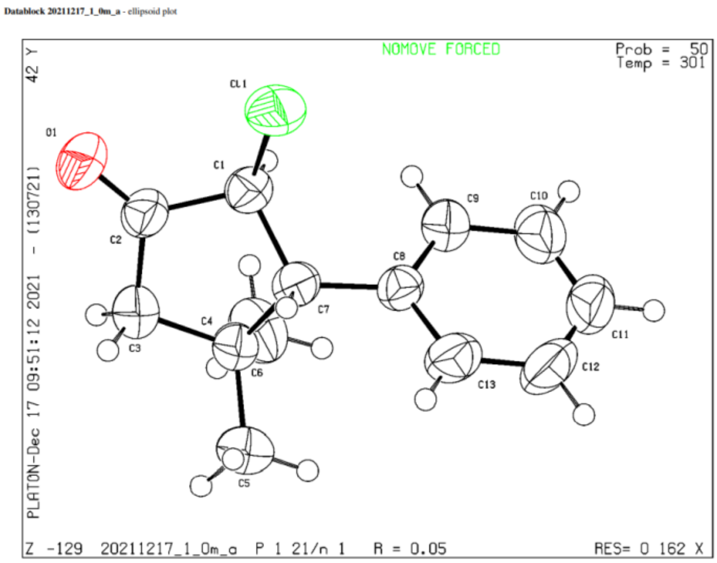

Supplementary Fig. 11. Crystal structure of **13** (CCDC **2164583**).

Supplementary Table 2. Crystal data for product **13**.

Empirical formula	$C_{13}H_{15}ClO$
Formula weight	222.08
Temperature	250 K
Crystal system, space group	Monoclinic, P2(1) / c
Unit cell dimensions	*a* = 6.1123(5) Å alpha = 90 deg.
	b = 7.4620(5) Å beta = 96.134(3) deg.
	c = 13.8843(11) Å gamma = 90 deg.
Volume	629.64(8) Å^3
Z	2
Absorption coefficient	0.114 mm^{-1}
Reflections collected	2811
Independent reflections	2560 [*R*(int) = 0.0215]
Refinement method	by full-matrix least-squares on F^2

Data / restraints / parameters	2560/1/165
Goodness-of-fit on F^2	1.027
Final R indices [I > 2s(I)]	wR_1 = 0.0334, wR_2 = 0.0805
Largest diff peak and hole	0.152 and -0.097 e.Å^{-3}

2. Supplementary figures

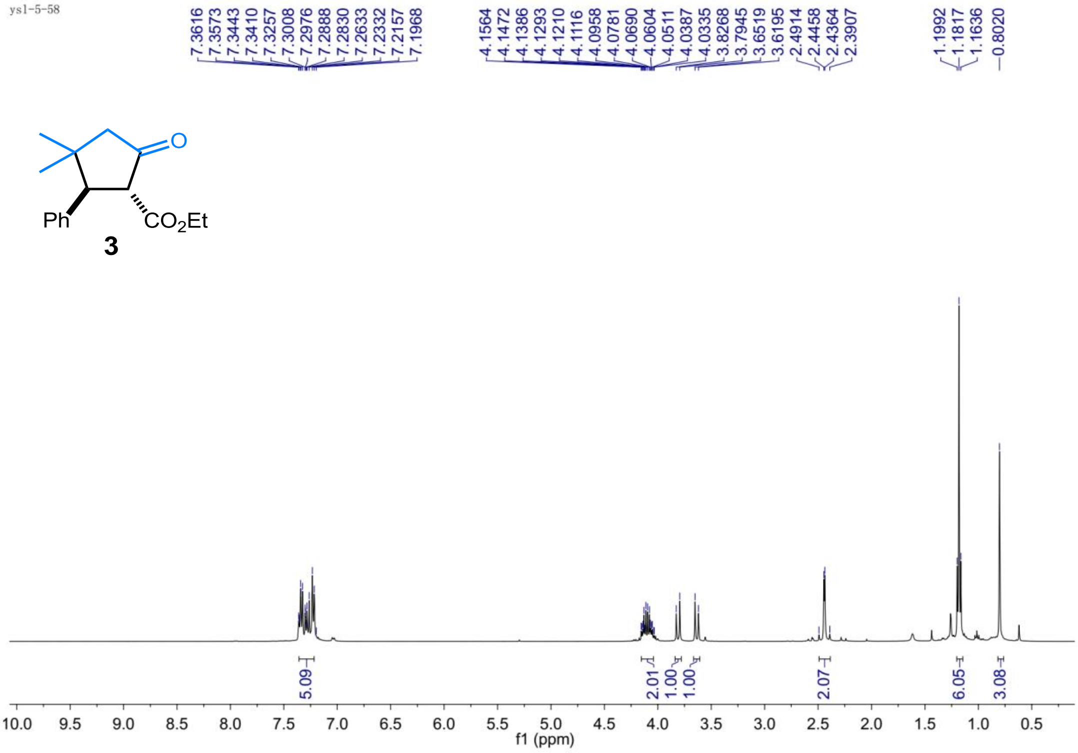

Supplementary Fig. 12. ^{1}H NMR spectrum (400 MHz, $CDCl_3$, 298K) of **3.**

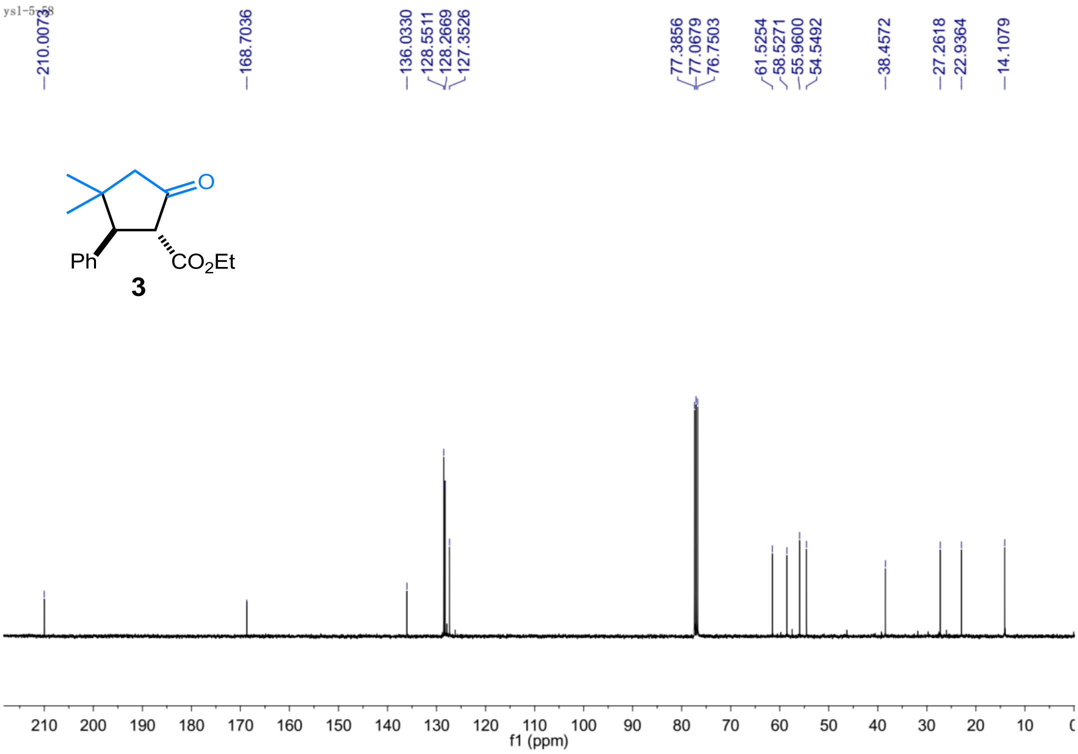

Supplementary Fig. 13. ^{13}C NMR spectrum (101 MHz, $CDCl_3$, 298K) of **3.**

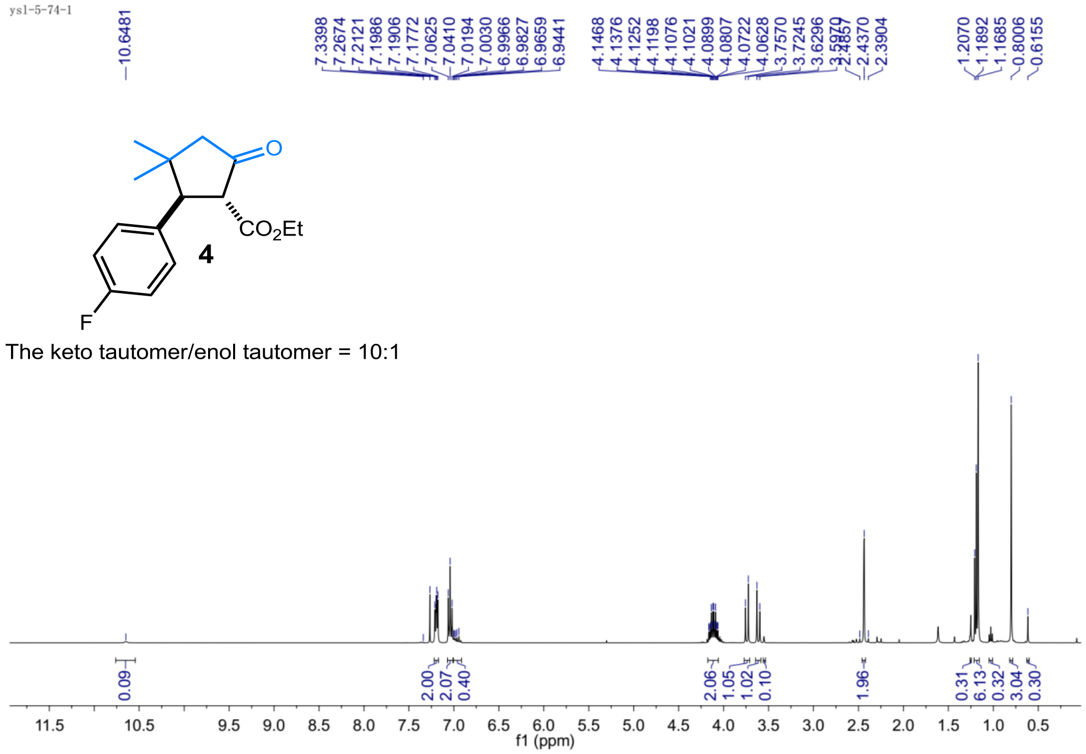

Supplementary Fig. 14. ^{1}H NMR spectrum (400 MHz, $CDCl_3$, 298K) of **4.**

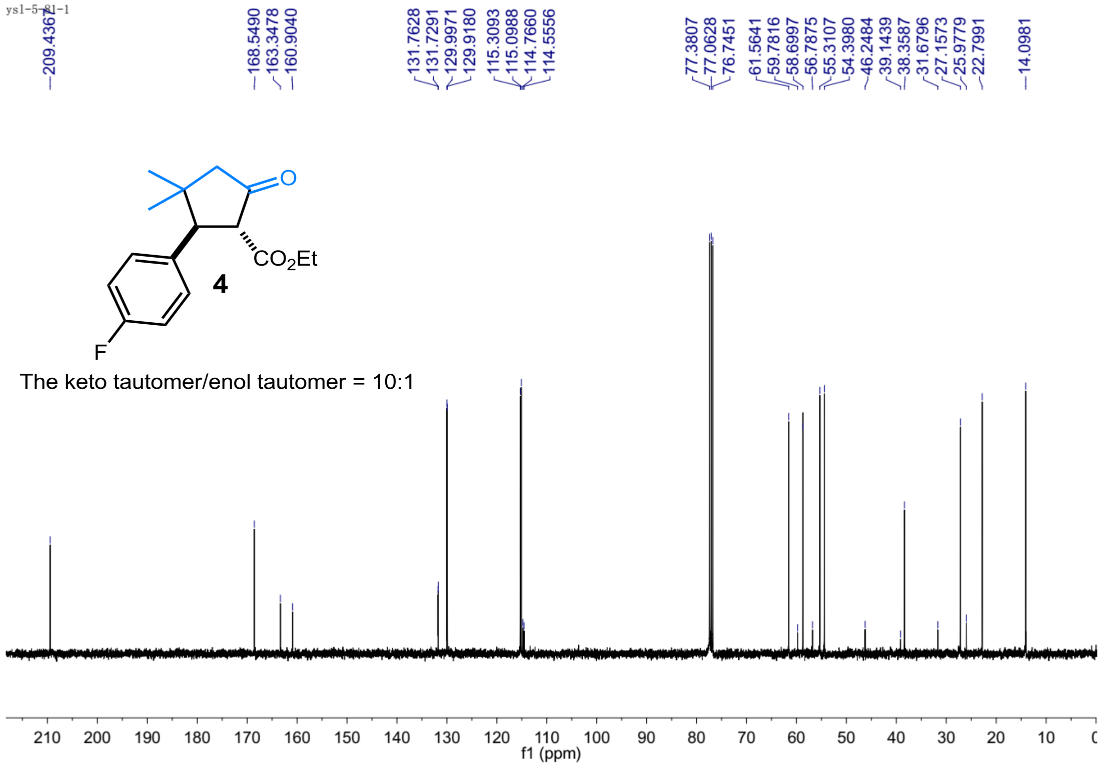

Supplementary Fig. 15. ^{13}C NMR spectrum (101 MHz, $CDCl_3$, 298K) of **4.**

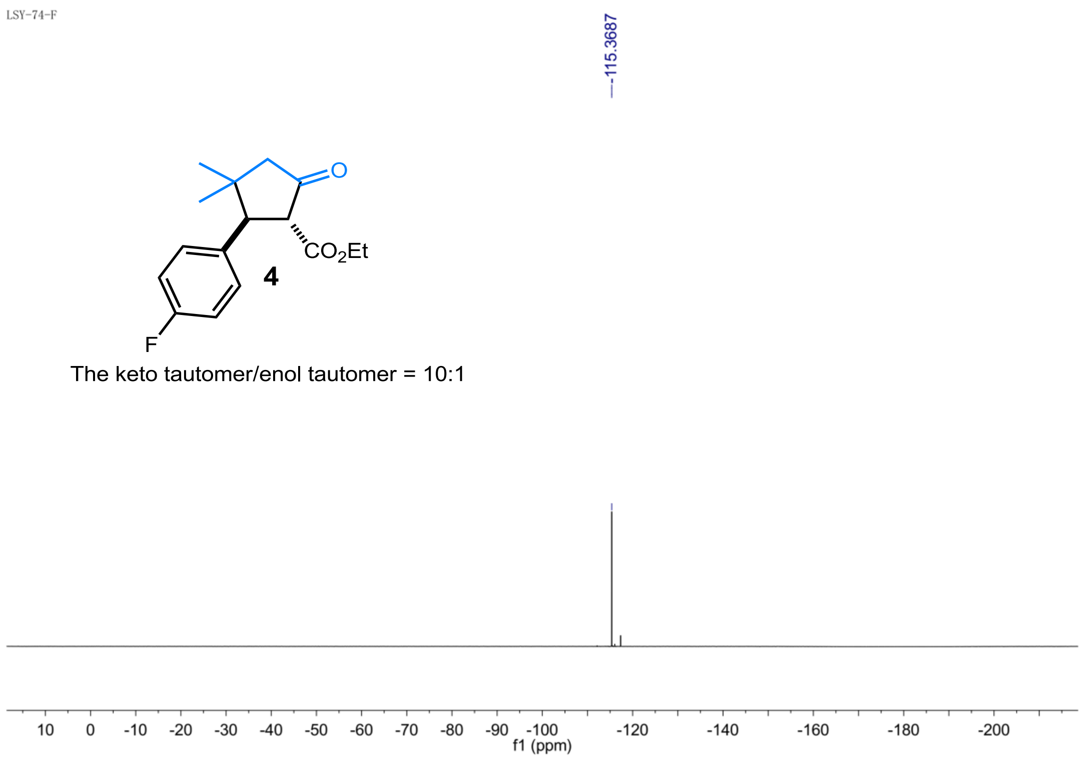

Supplementary Fig. 16. ^{19}F NMR spectrum (565 MHz, $CDCl_3$, 298K) of **4.**

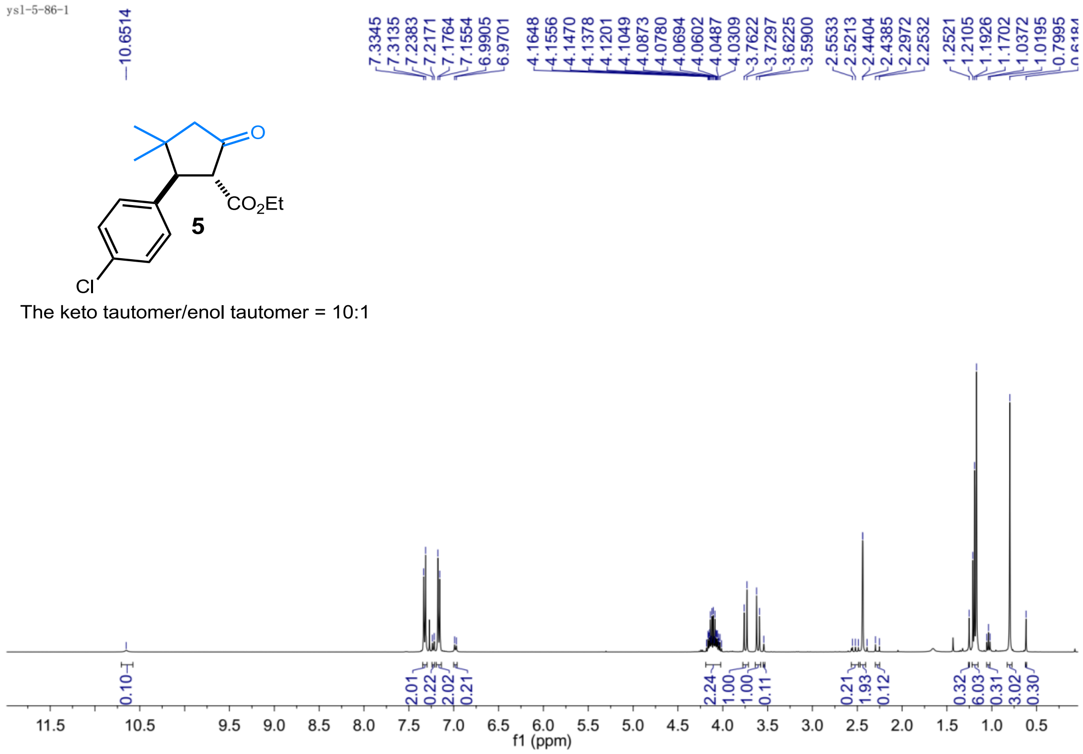

Supplementary Fig. 17. ^{1}H NMR spectrum (400 MHz, $CDCl_3$, 298K) of **5.**

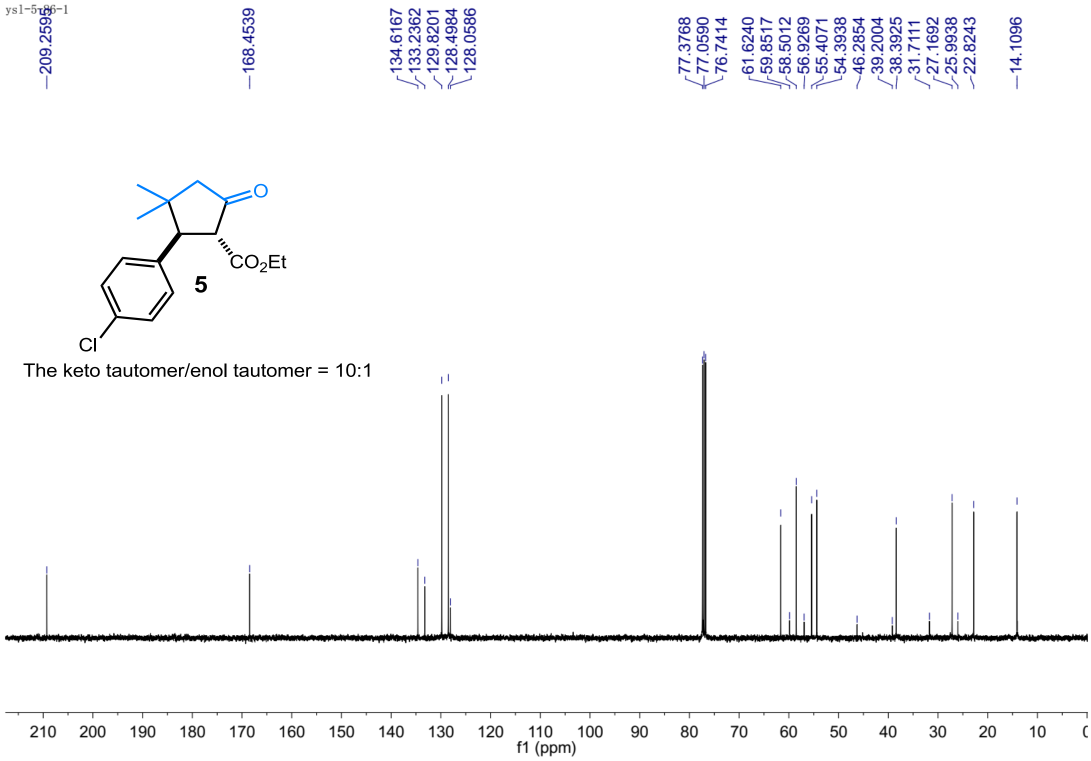

Supplementary Fig. 18. ^{13}C NMR spectrum (101 MHz, $CDCl_3$, 298K) of **5.**

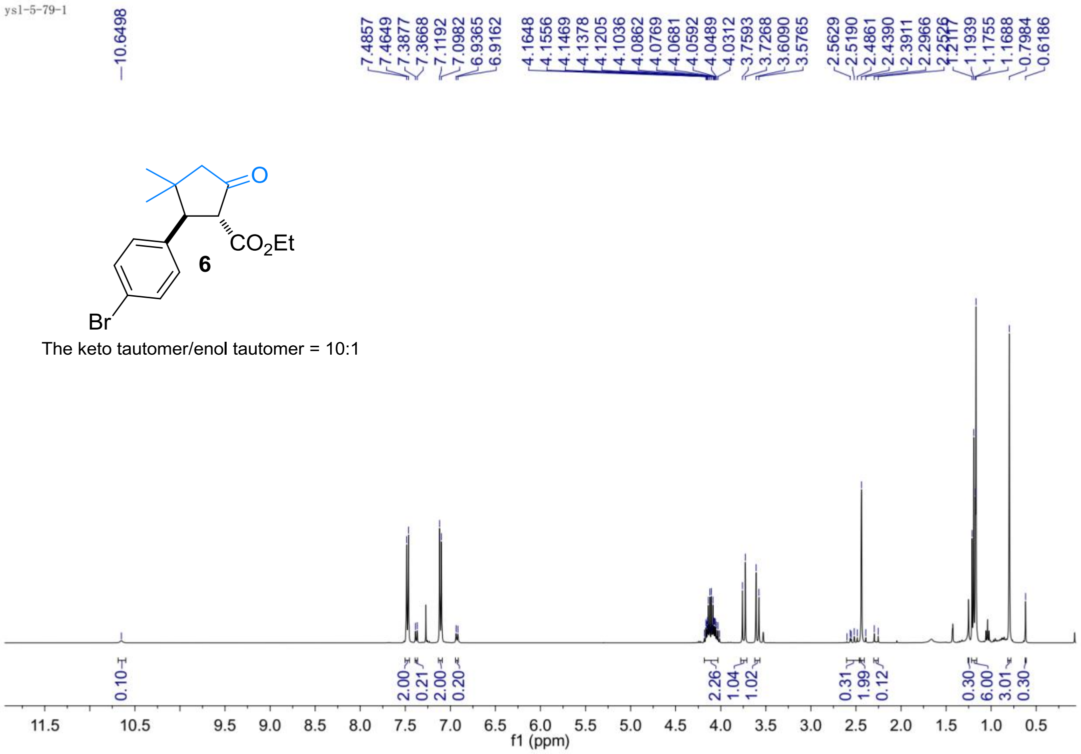

Supplementary Fig. 19. ^{1}H NMR spectrum (400 MHz, $CDCl_3$, 298K) of **6.**

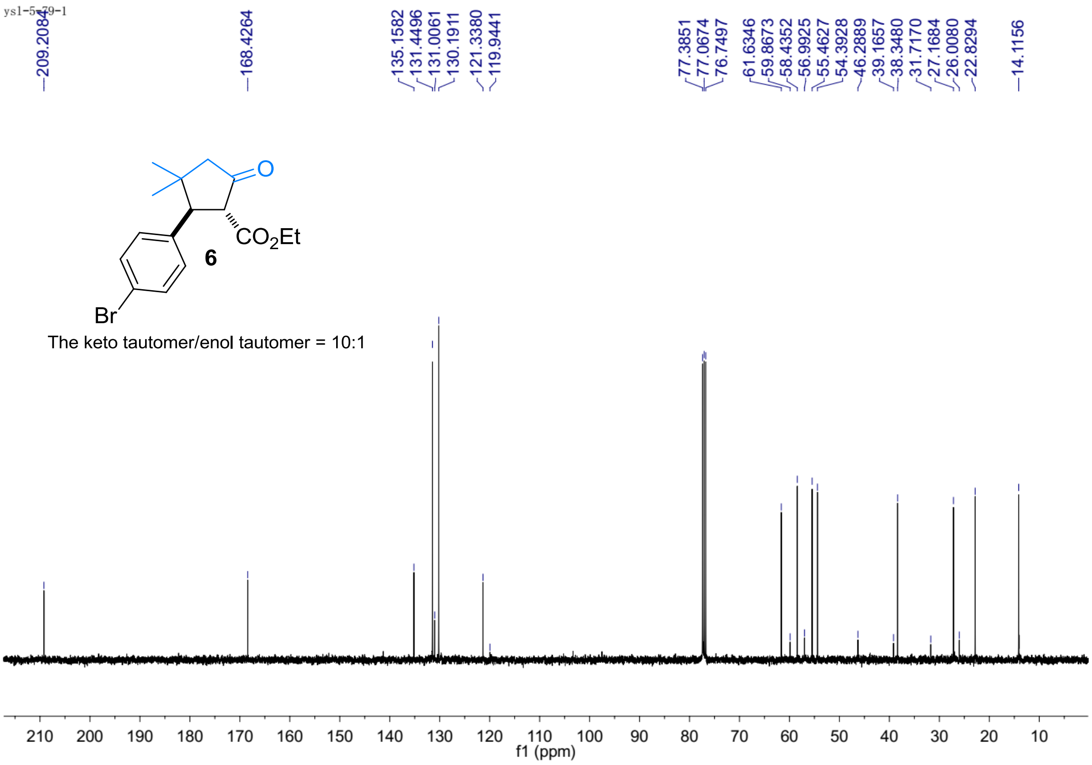

Supplementary Fig. 20. ^{13}C NMR spectrum (101 MHz, $CDCl_3$, 298K) of **6.**

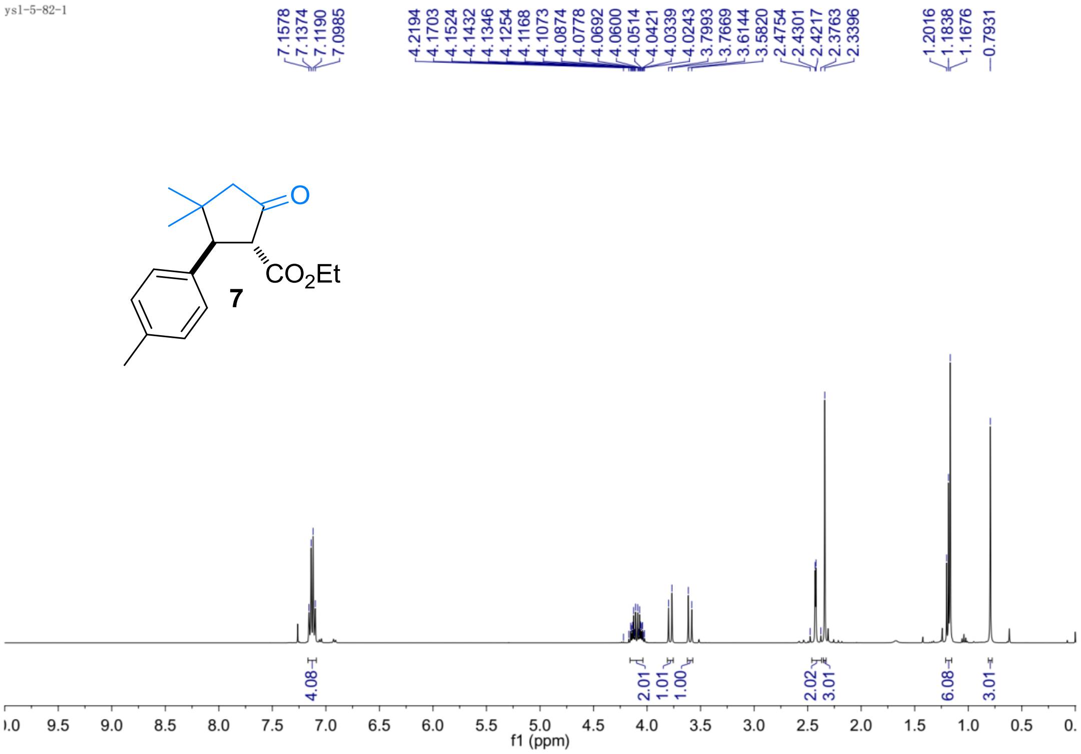

Supplementary Fig. 21. ^{1}H NMR spectrum (400 MHz, $CDCl_3$, 298K) of **7.**

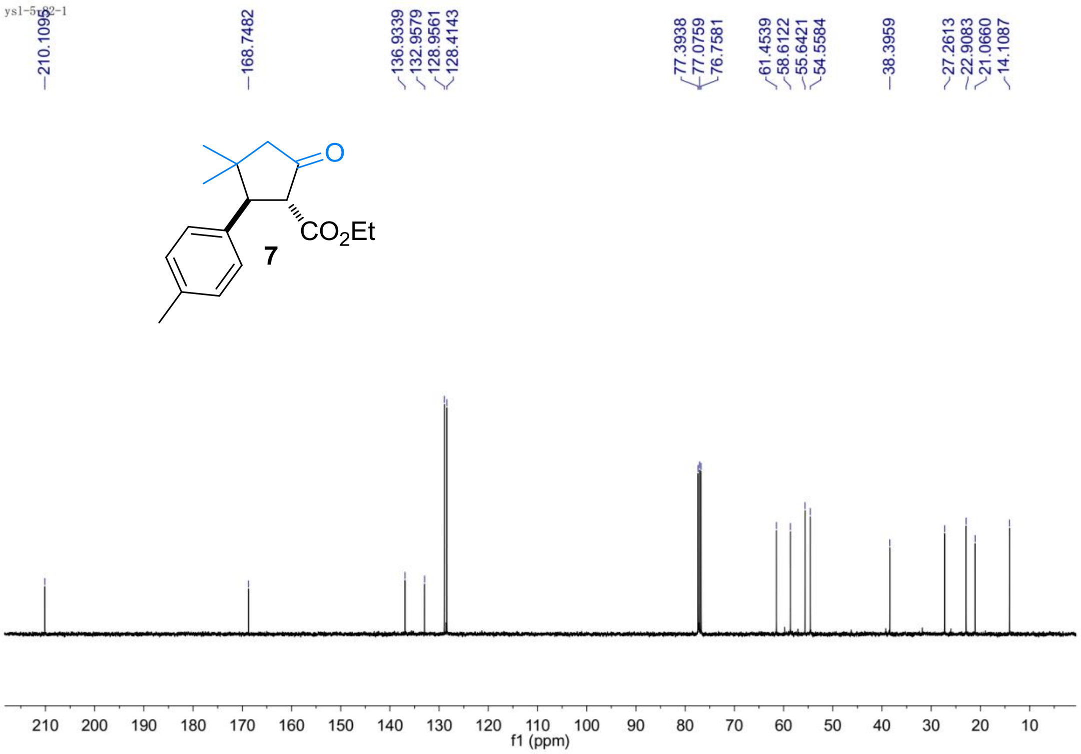

Supplementary Fig. 22. ^{13}C NMR spectrum (101 MHz, $CDCl_3$, 298K) of **7.**

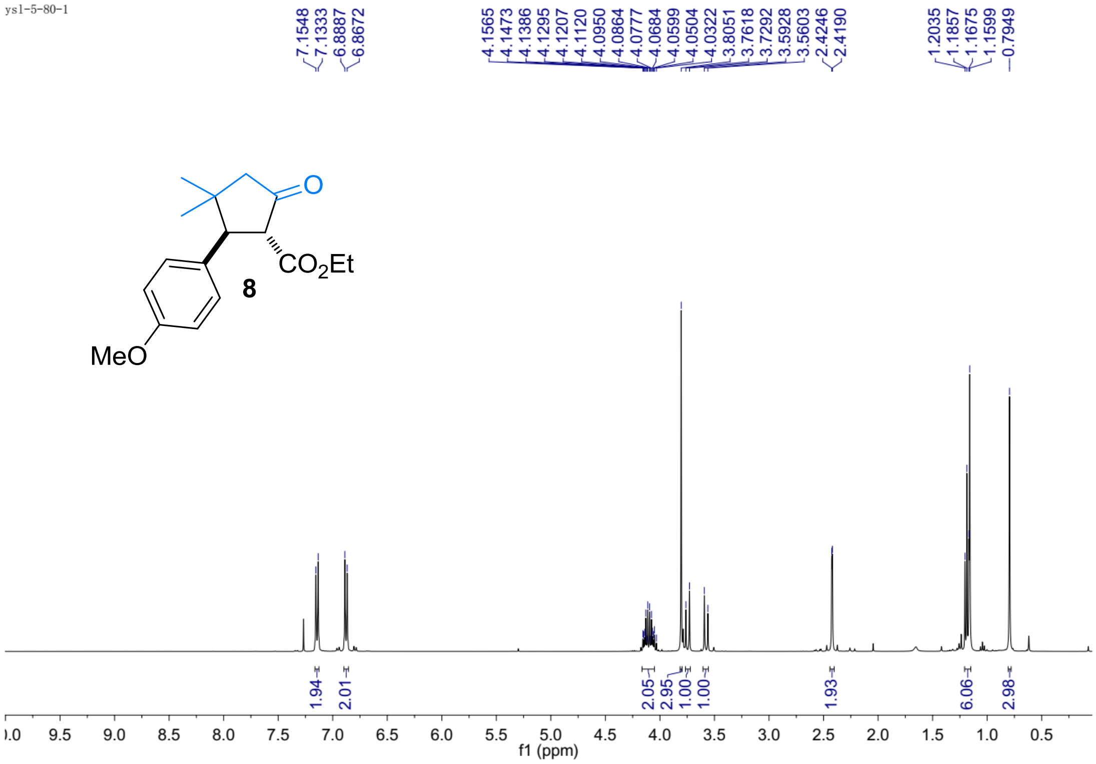

Supplementary Fig. 23. ^{1}H NMR spectrum (400 MHz, $CDCl_3$, 298K) of **8.**

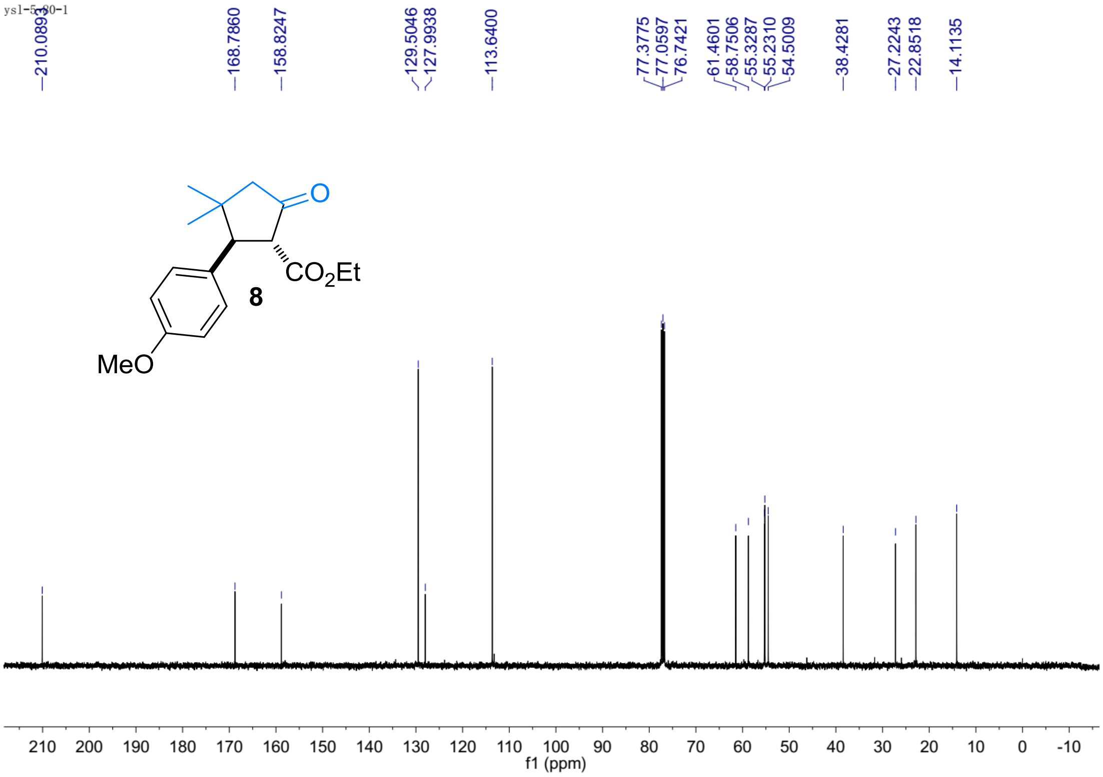

Supplementary Fig. 24. ^{13}C NMR spectrum (101 MHz, $CDCl_3$, 298K) of **8.**

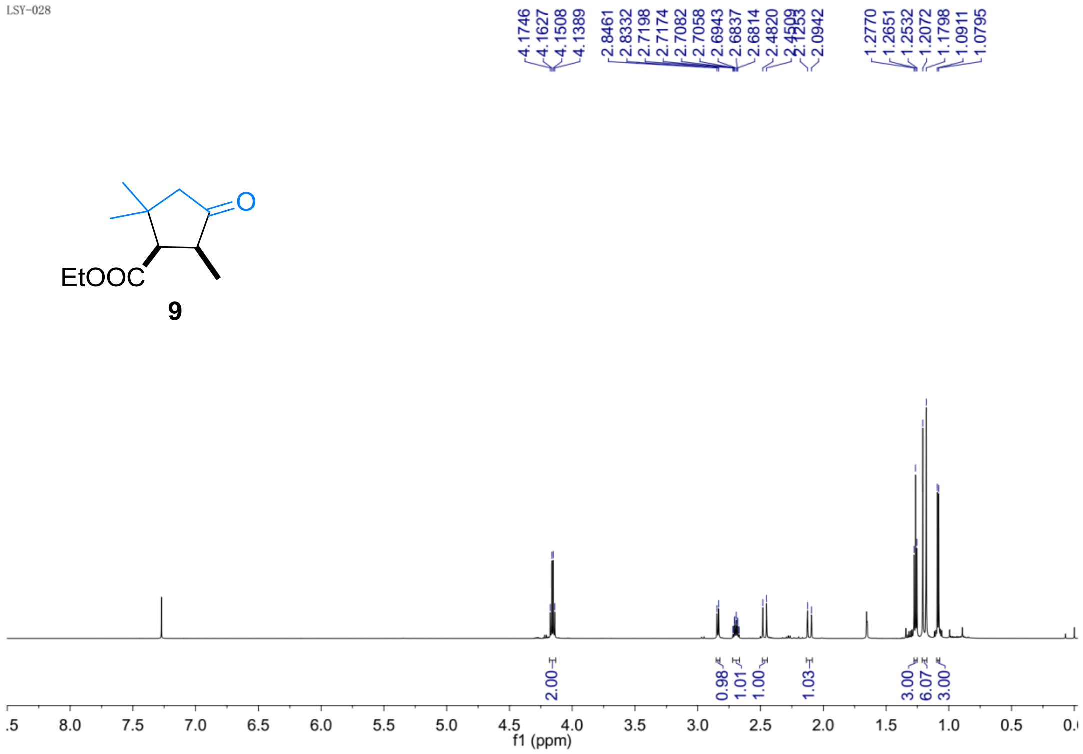

Supplementary Fig. 25. ^{1}H NMR spectrum (600 MHz, $CDCl_3$, 298K) of **9.**

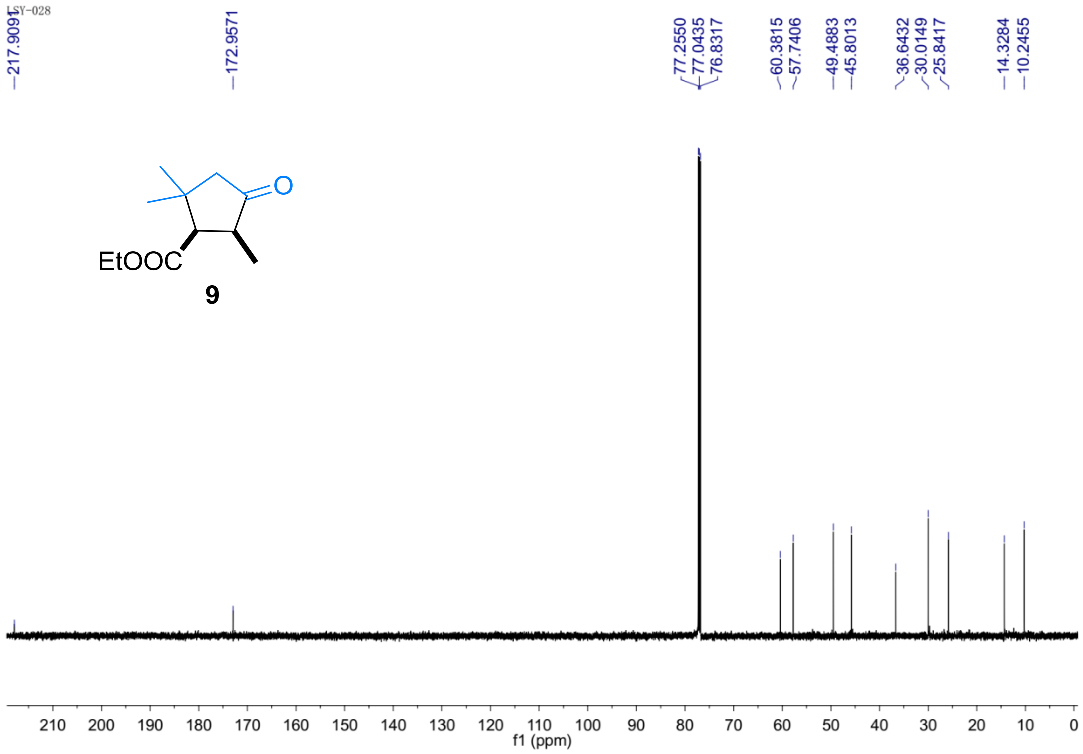

Supplementary Fig. 26. ^{13}C NMR spectrum (151 MHz, $CDCl_3$, 298K) of **9.**

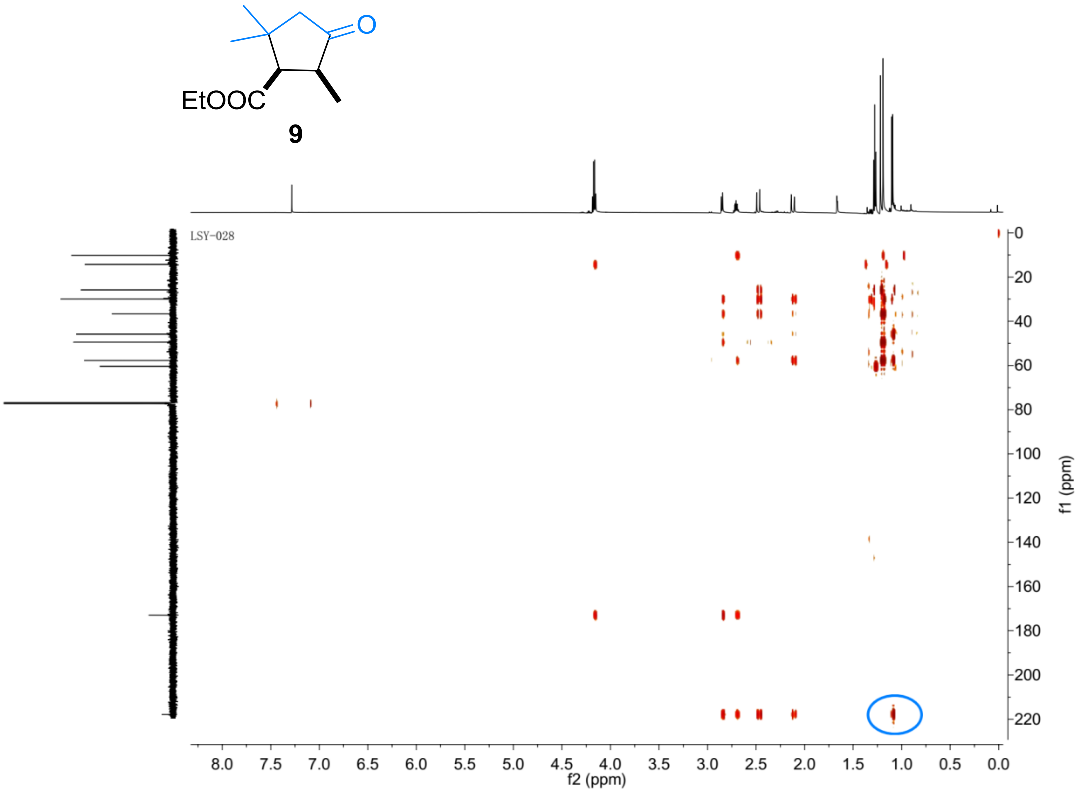

Supplementary Fig. 27. 2D HMBC spectrum ($CDCl_3$, 298K) of **9.**

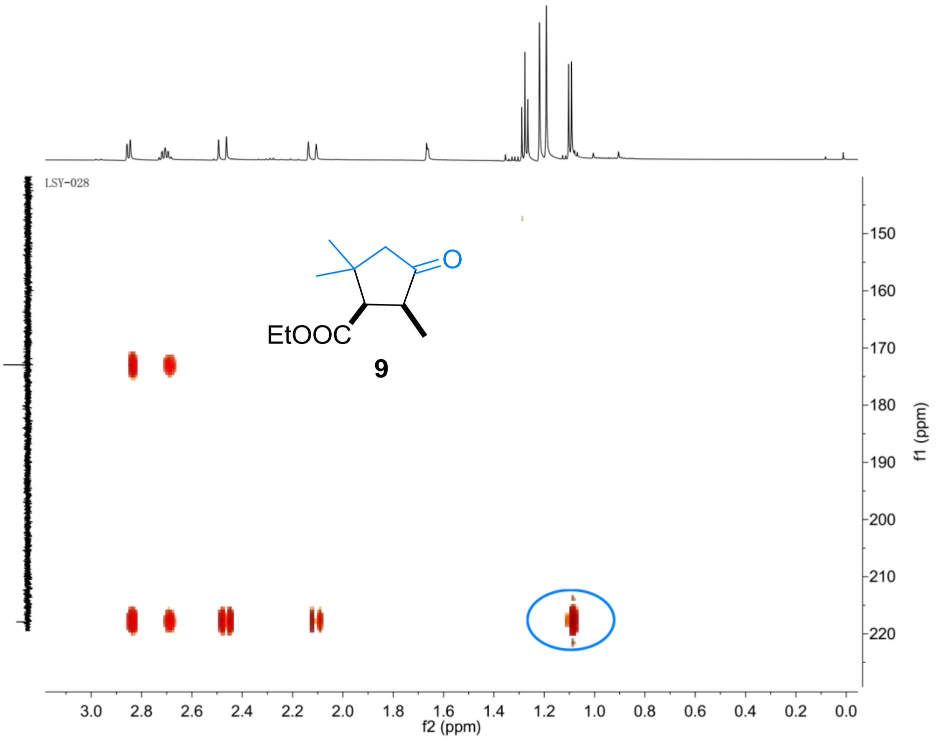

Supplementary Fig. 28. Expansion of 2D HMBC spectrum ($CDCl_3$, 298K) of **9.**

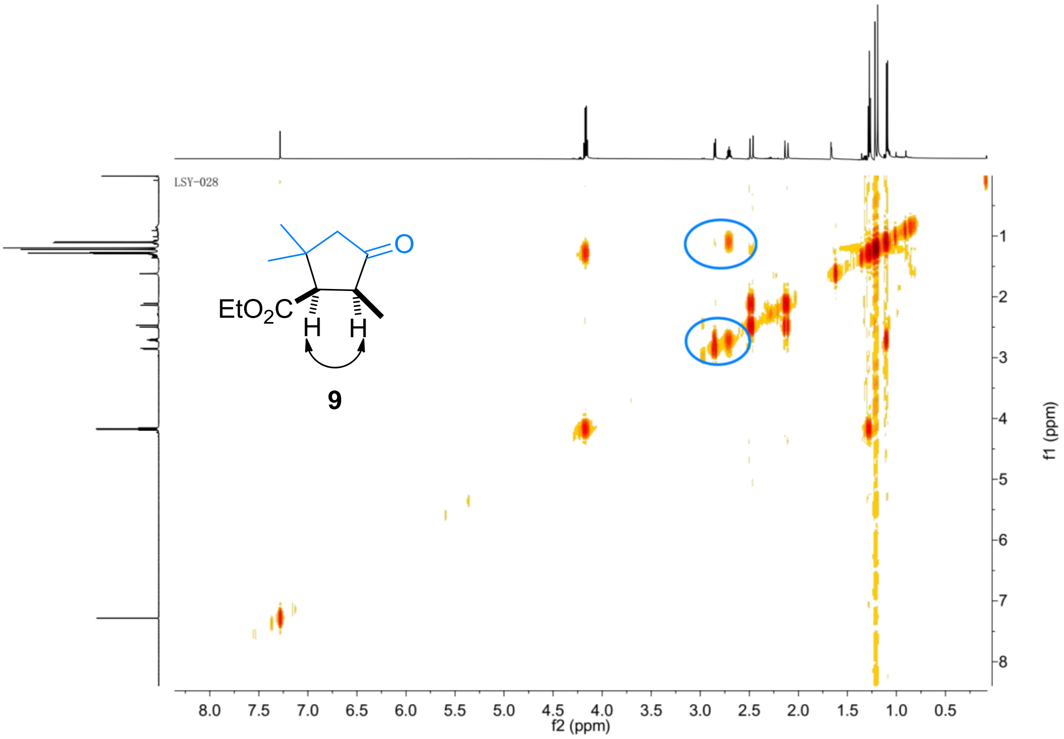

Supplementary Fig. 29. NOESY spectrum ($CDCl_3$, 298K) of **9.**

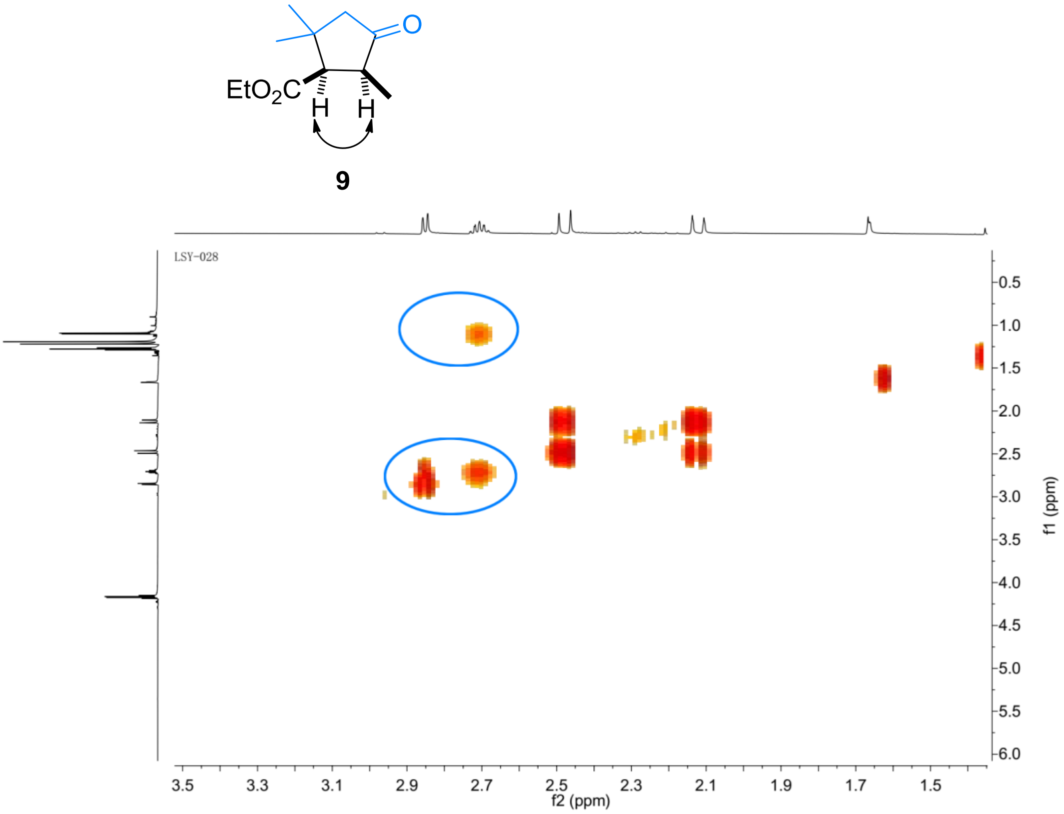

Supplementary Fig. 30. Expansion of **NOESY** spectrum ($CDCl_3$, 298K) of **9.**

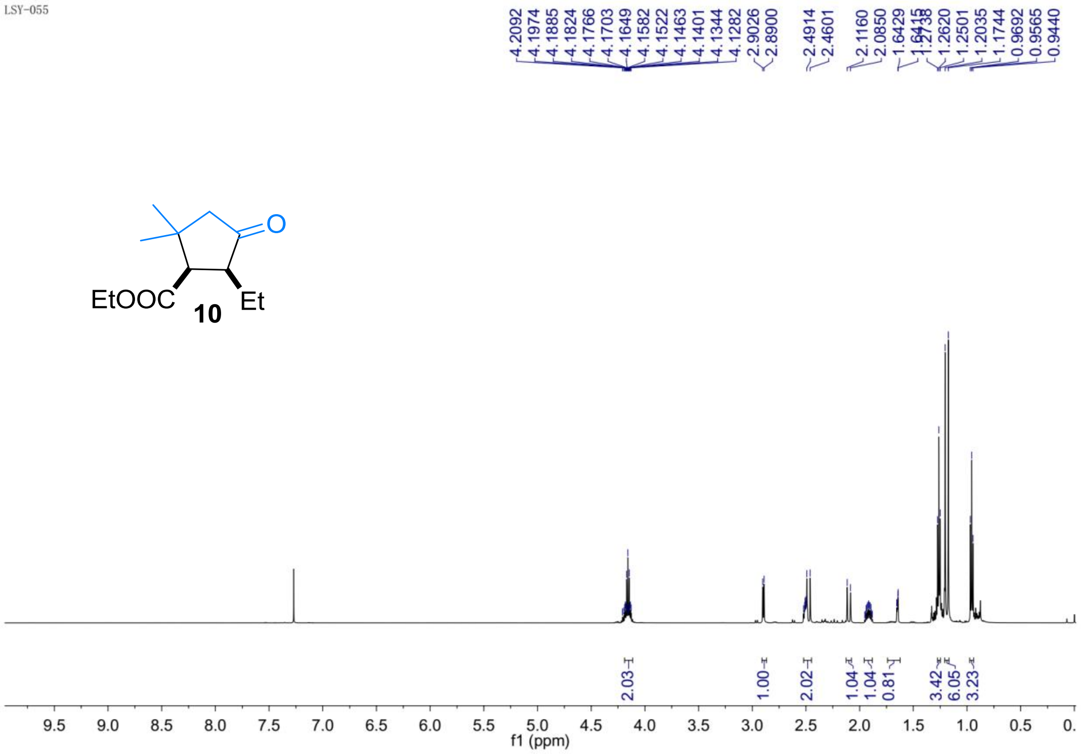

Supplementary Fig. 31. ^{1}H NMR spectrum (600 MHz, $CDCl_3$, 298K)of **10.**

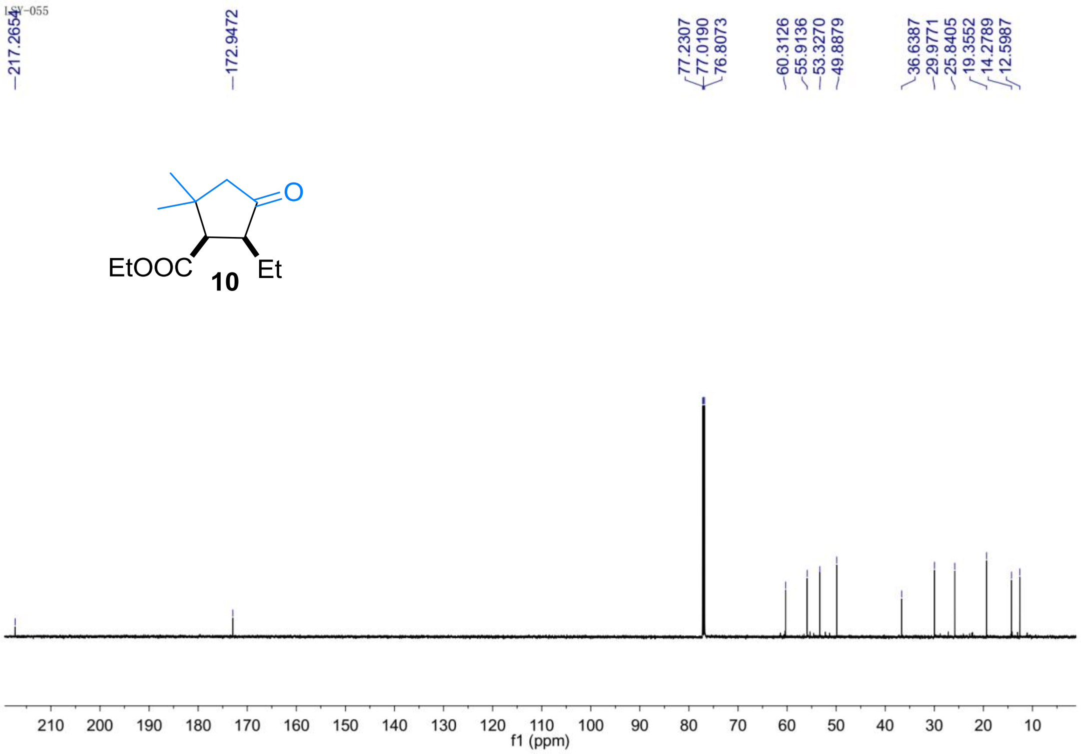

Supplementary Fig. 32. ^{13}C NMR spectrum (151 MHz, $CDCl_3$, 298K) of **10.**

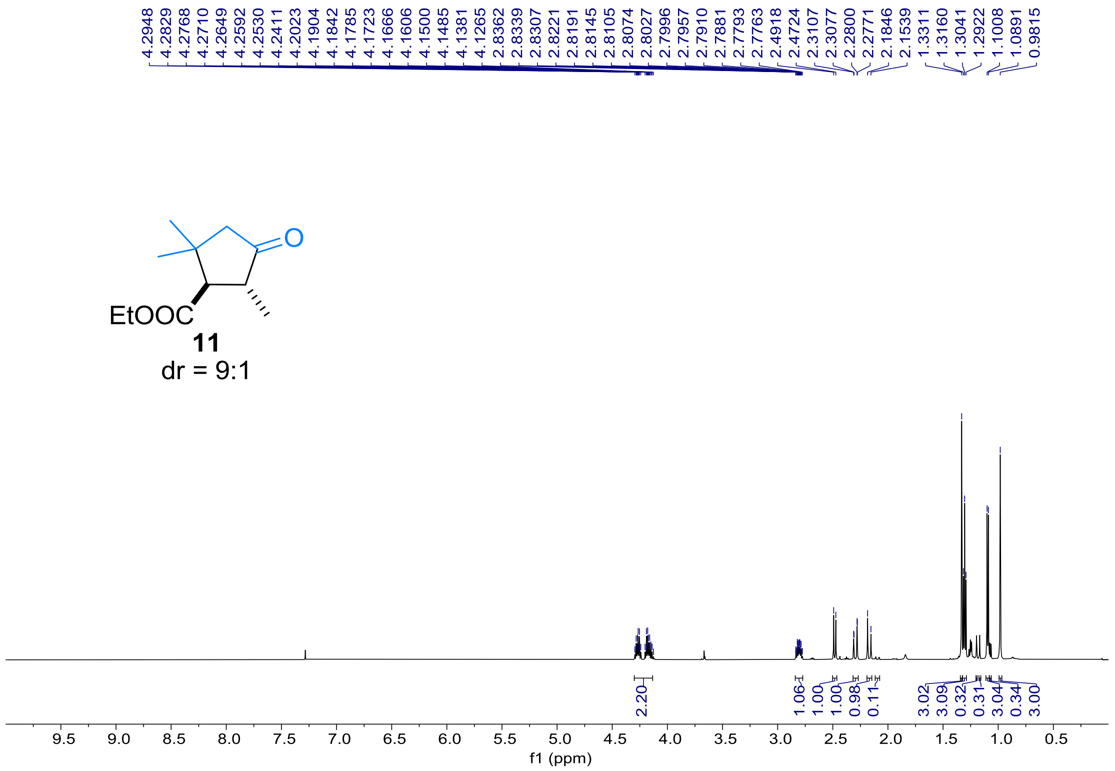

Supplementary Fig. 33. ^{1}H NMR spectrum (600 MHz, $CDCl_3$, 298K)of **11.**

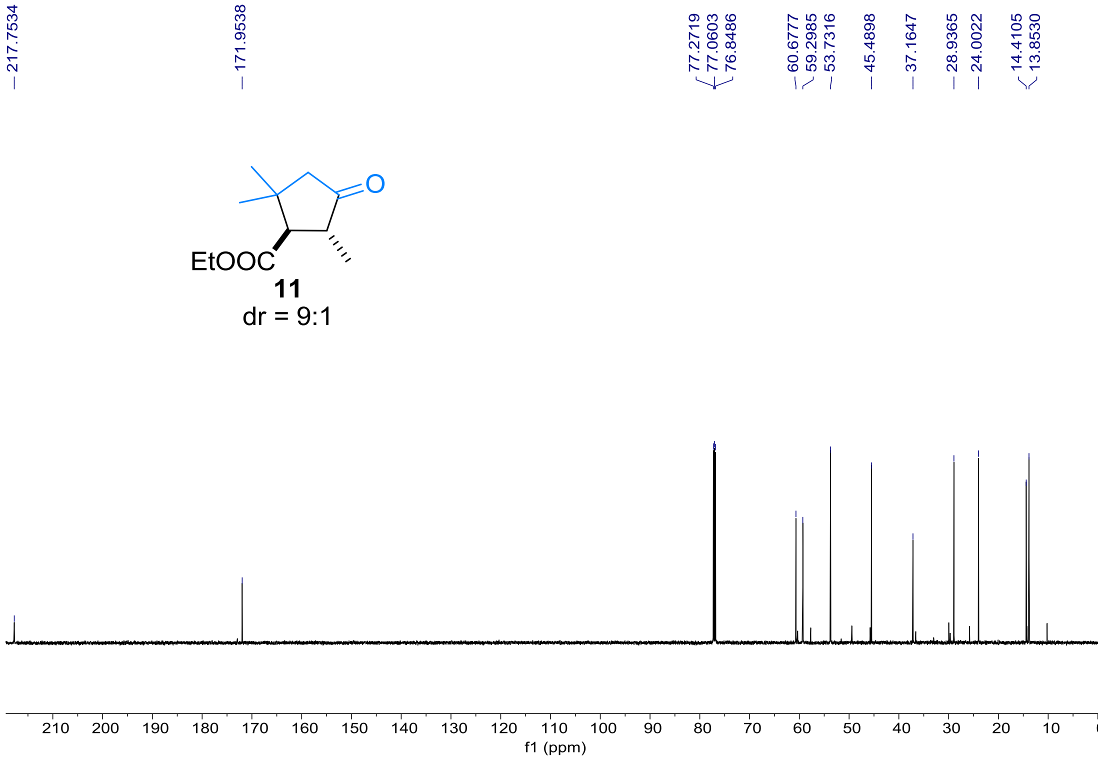

Supplementary Fig. 34. ^{13}C NMR spectrum (151 MHz, $CDCl_3$, 298K) of **11.**

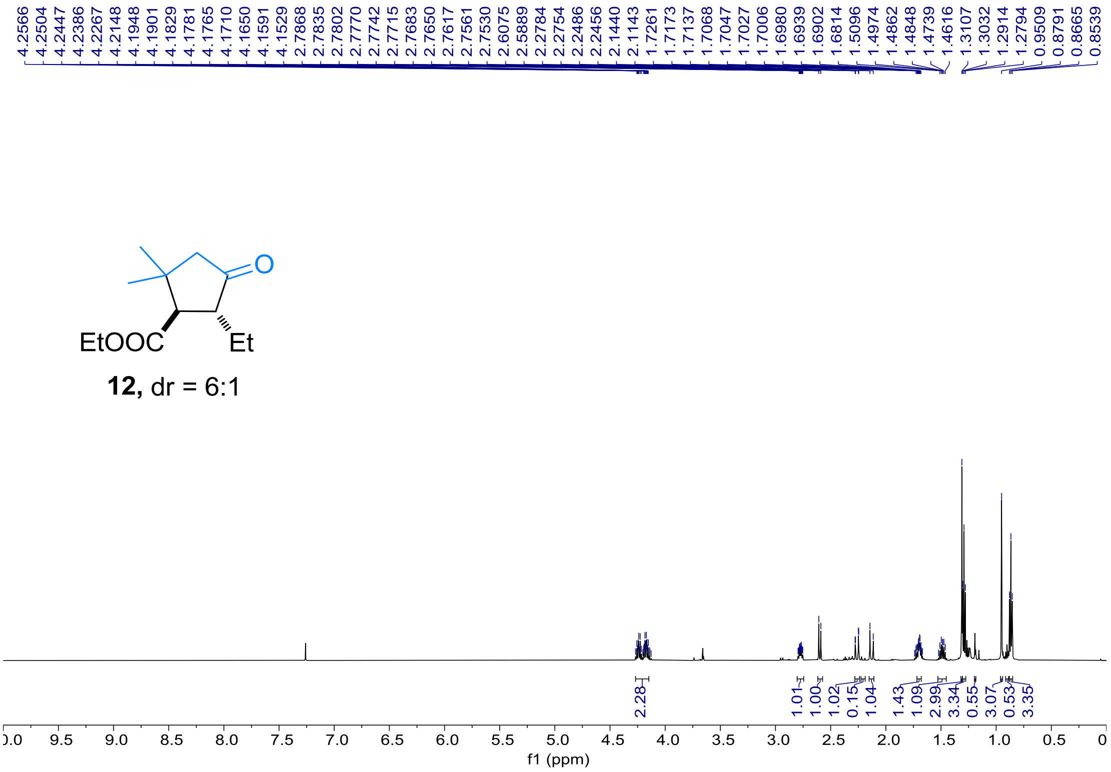

Supplementary Fig. 35. ^{1}H NMR spectrum (600 MHz, $CDCl_3$, 298K) of **12.**

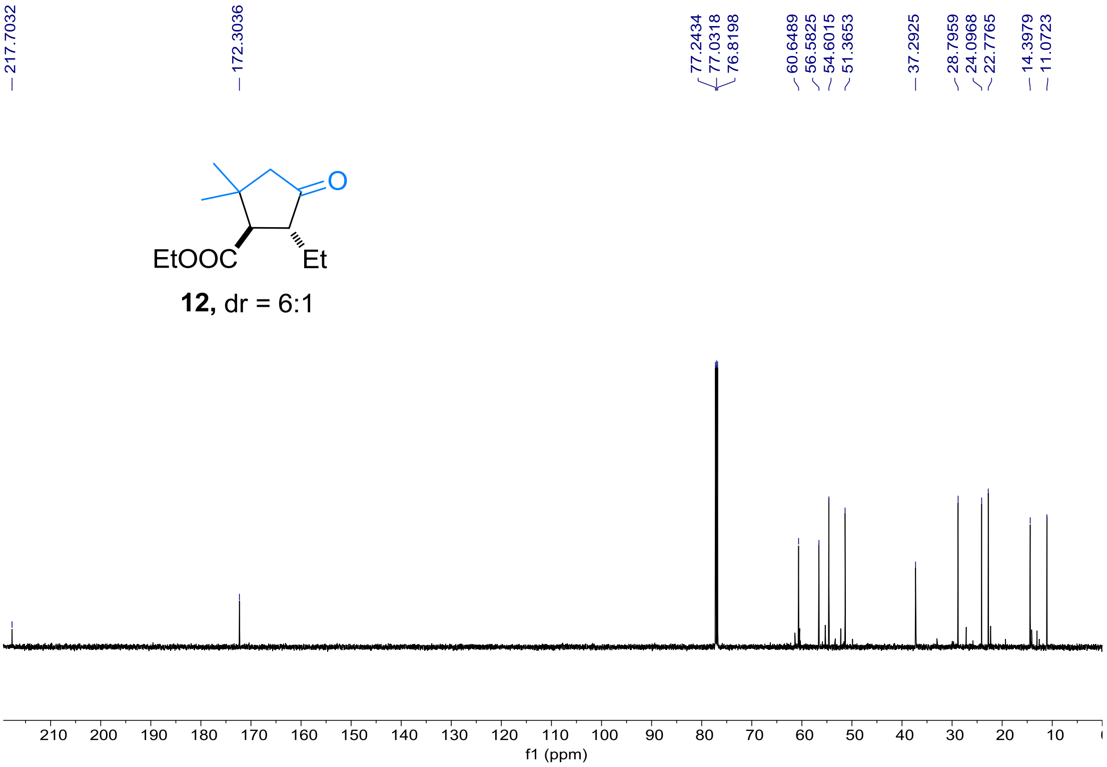

Supplementary Fig. 36. ^{13}C NMR spectrum (151 MHz, $CDCl_3$, 298K) of **12.**

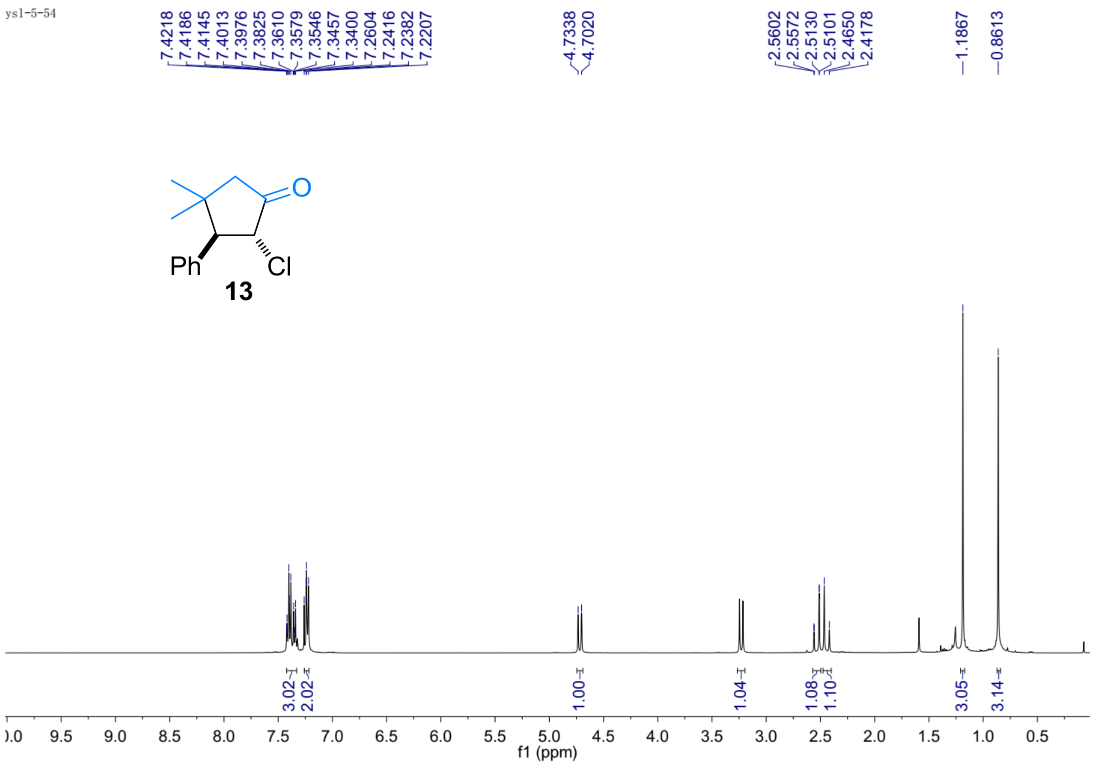

Supplementary Fig. 37. ^{1}H NMR spectrum (400 MHz, $CDCl_3$, 298K) of **13.**

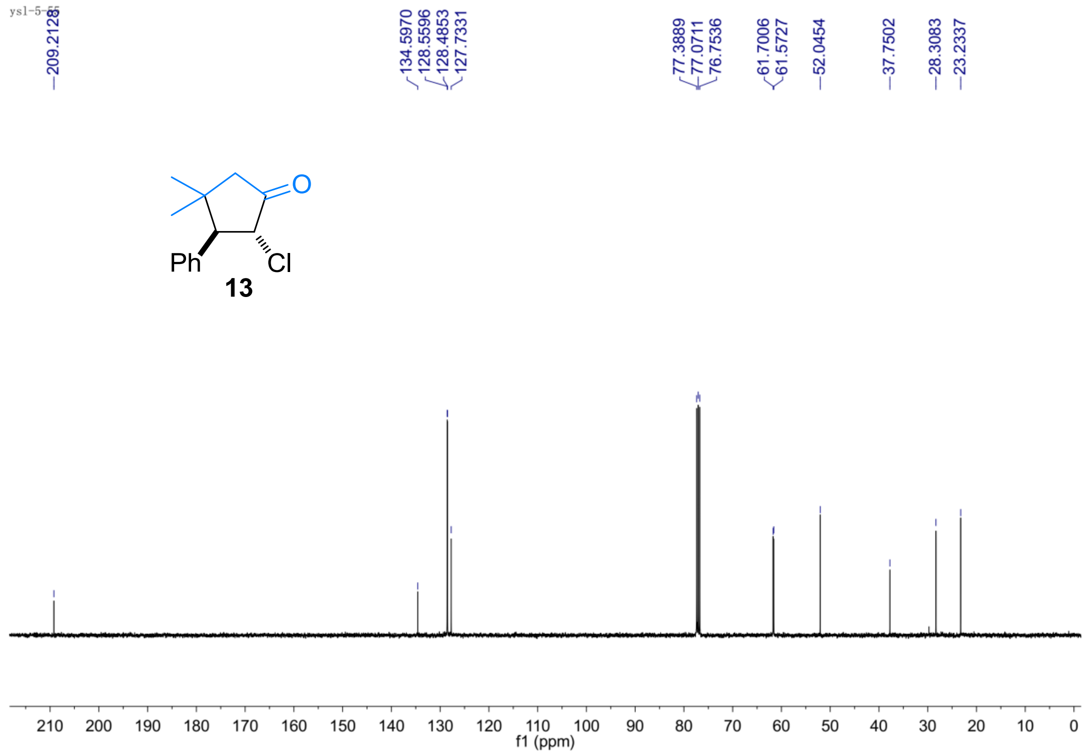

Supplementary Fig. 38. ^{13}C NMR spectrum (101 MHz, $CDCl_3$, 298K) of **13.**

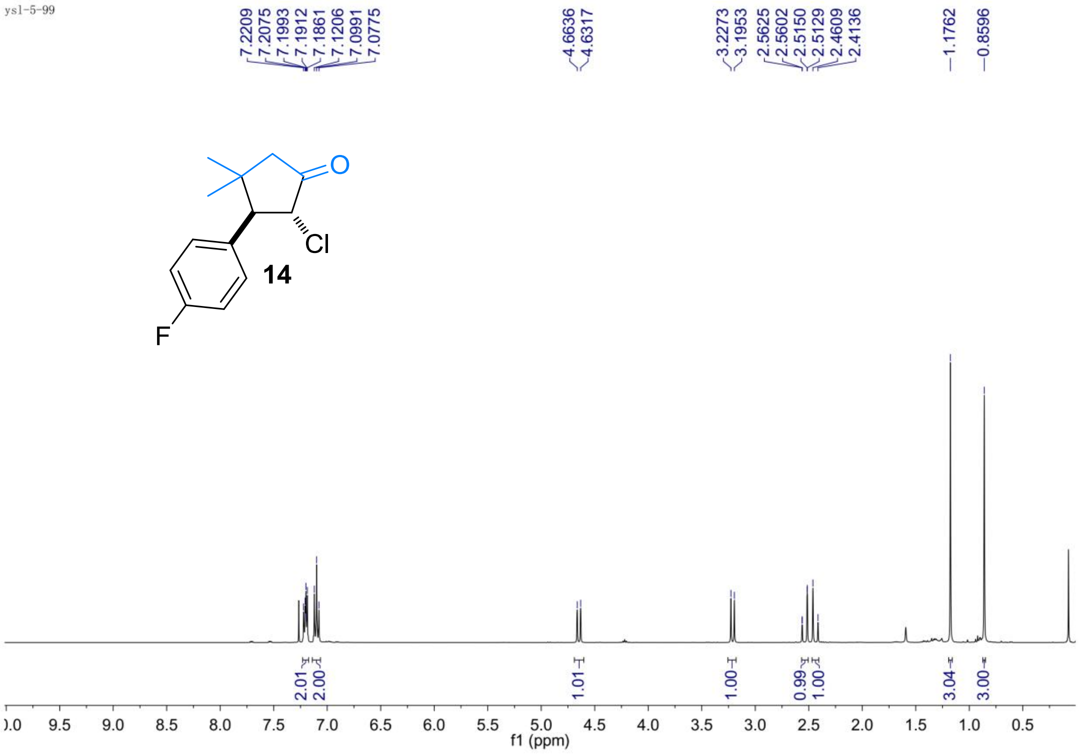

Supplementary Fig. 39. ^{1}H NMR spectrum (400 MHz, $CDCl_3$, 298K) of **14.**

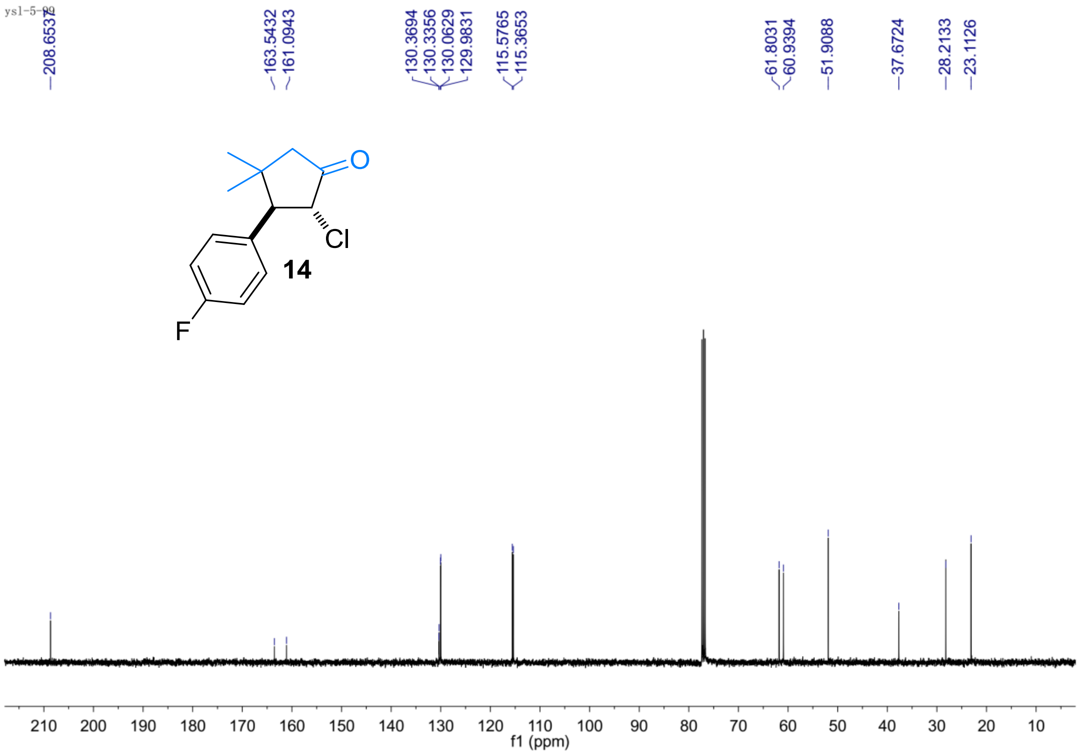

Supplementary Fig. 40. ^{13}C NMR spectrum (101 MHz, $CDCl_3$, 298K) of **14.**

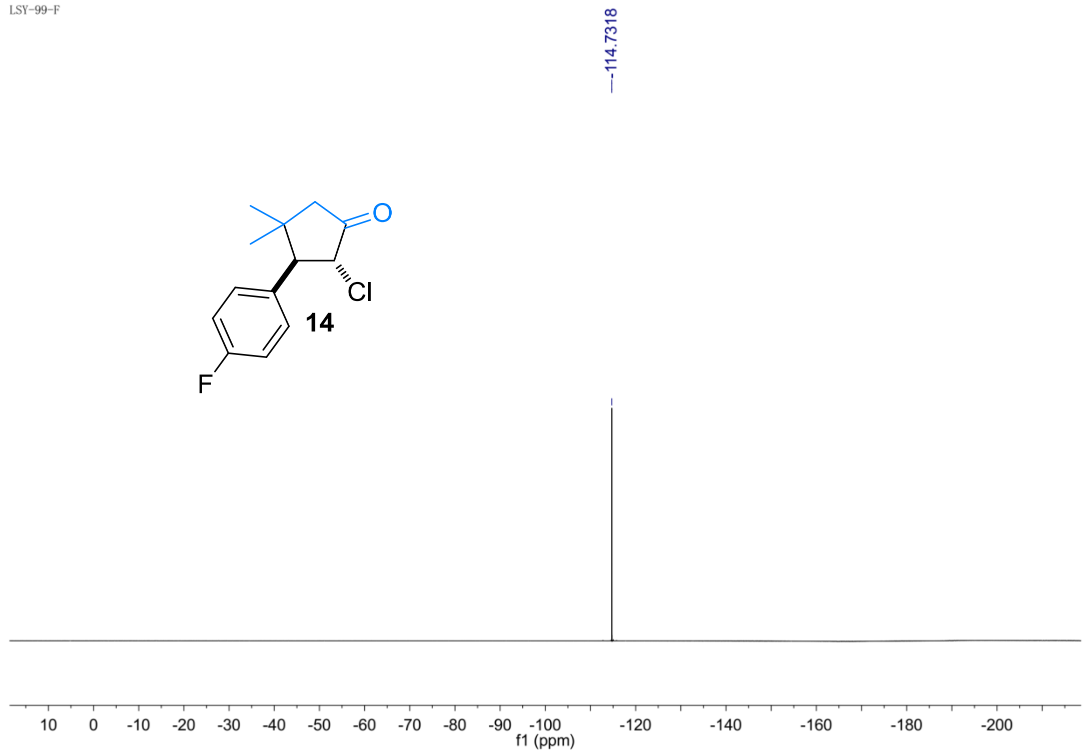

Supplementary Fig. 41. ^{19}F NMR spectrum (565 MHz, $CDCl_3$, 298K) of **14.**

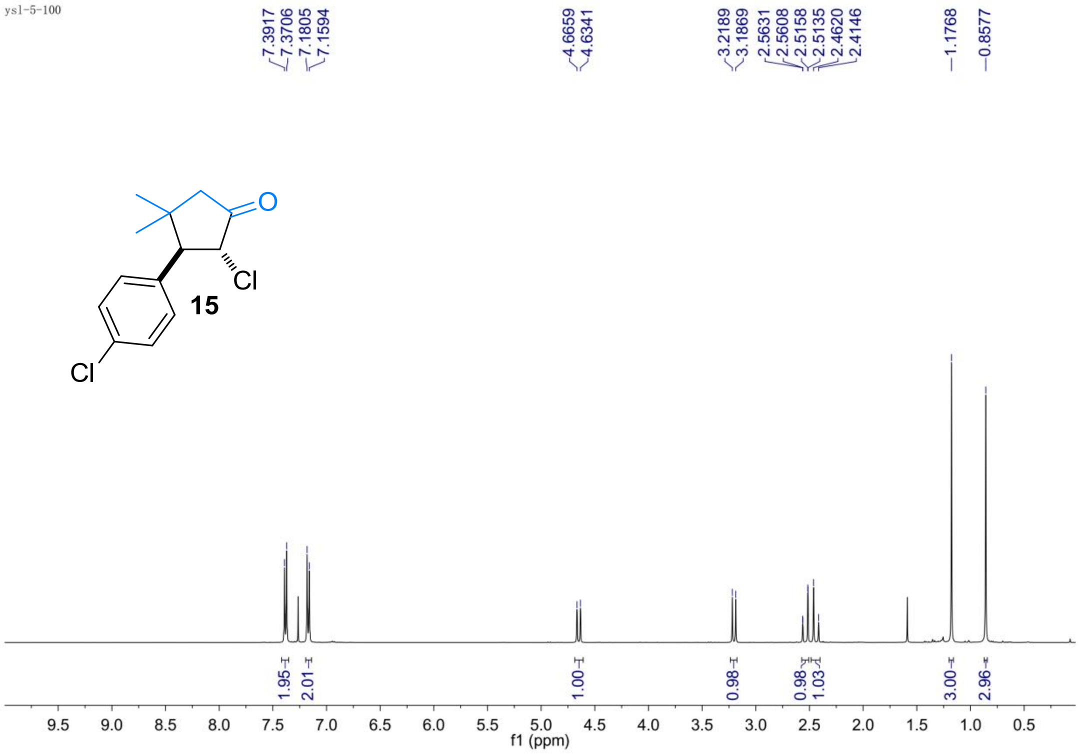

Supplementary Fig. 42. ^{1}H NMR spectrum (400 MHz, $CDCl_3$, 298K) of **15.**

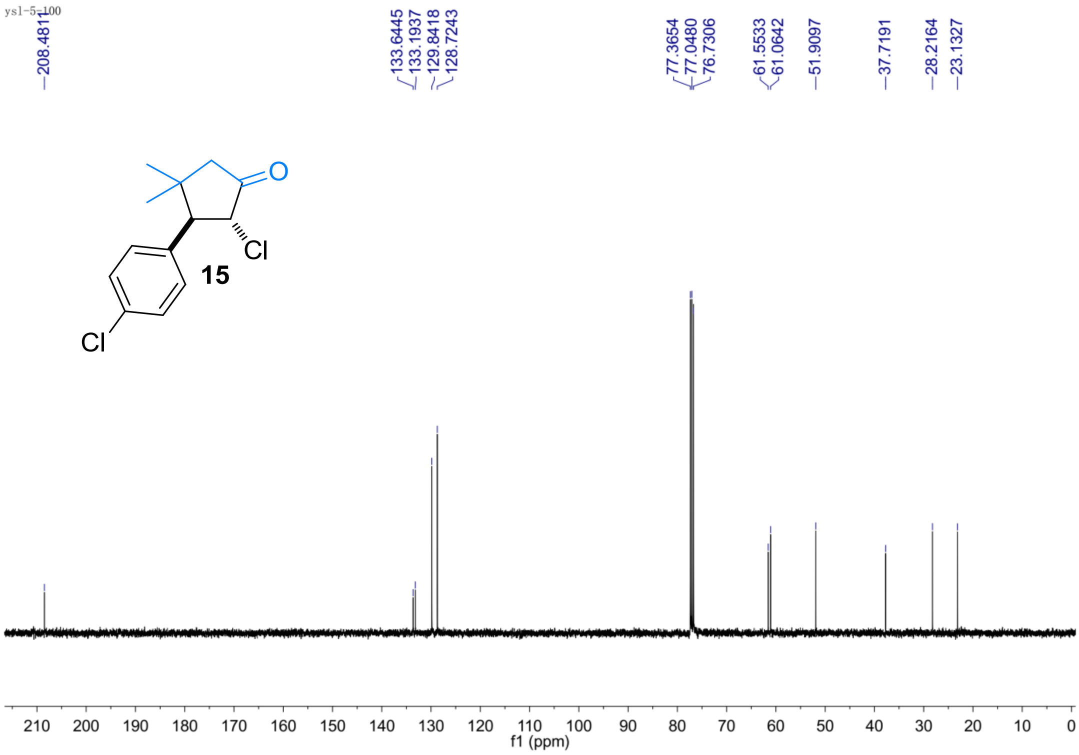

Supplementary Fig. 43. ^{13}C NMR spectrum (101 MHz, $CDCl_3$, 298K) of **15.**

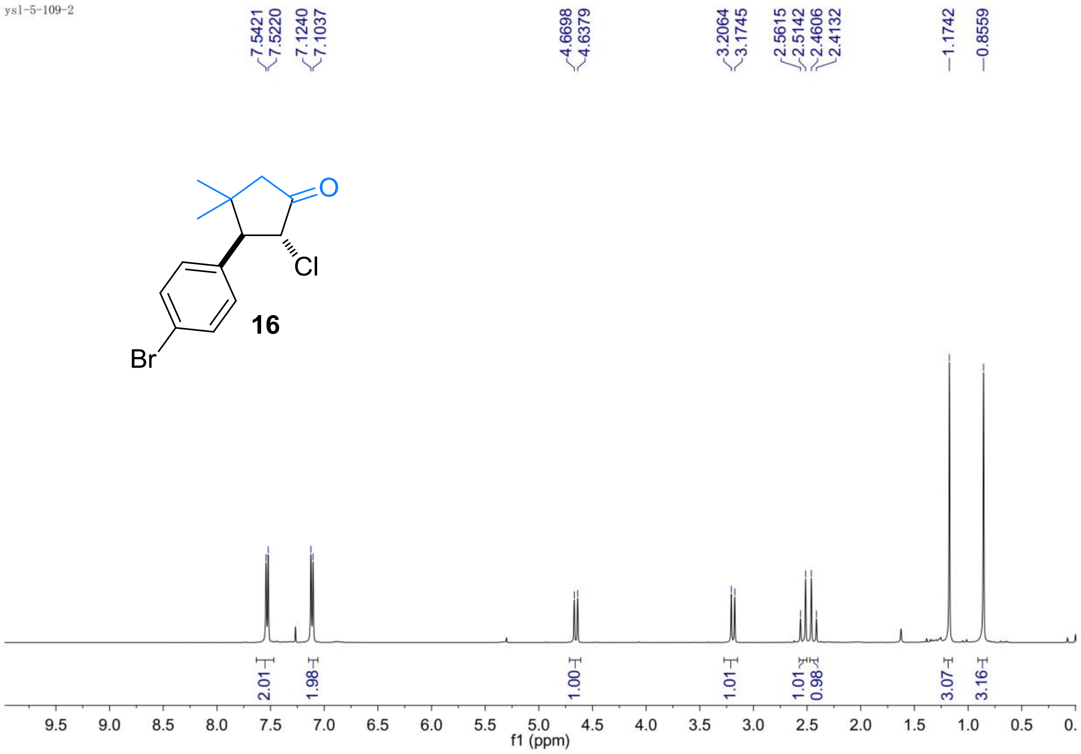

Supplementary Fig. 44. ^{1}H NMR spectrum (400 MHz, $CDCl_3$, 298K) of **16.**

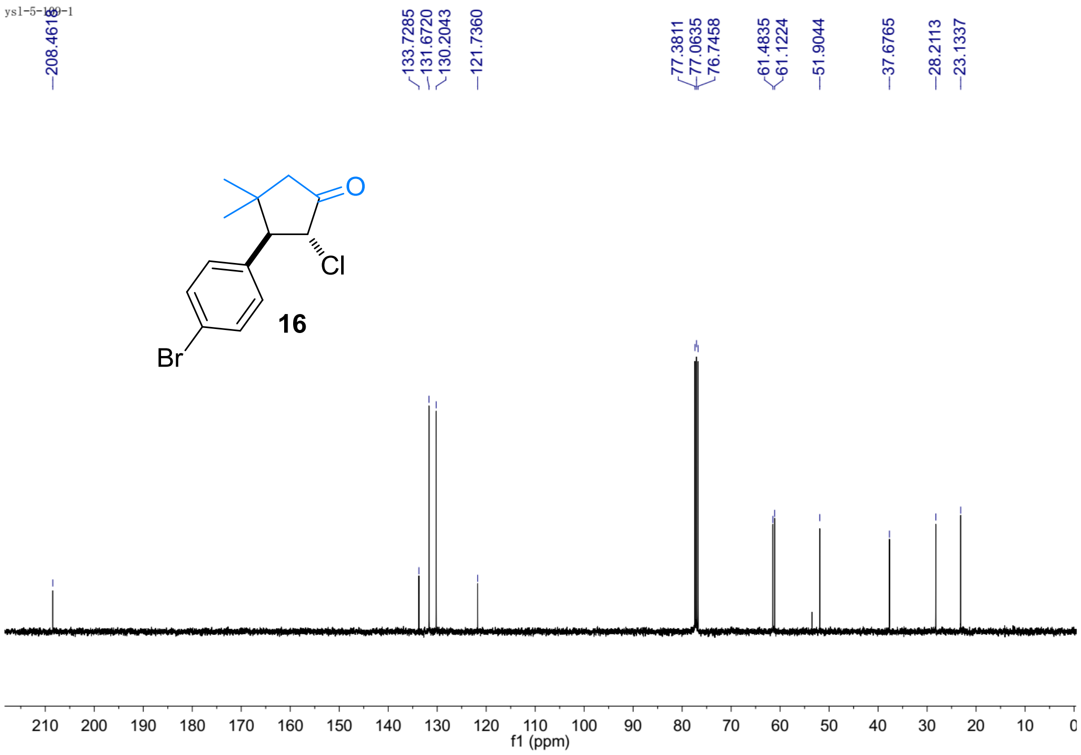

Supplementary Fig. 45. ^{13}C NMR spectrum (101 MHz, $CDCl_3$, 298K) of **16.**

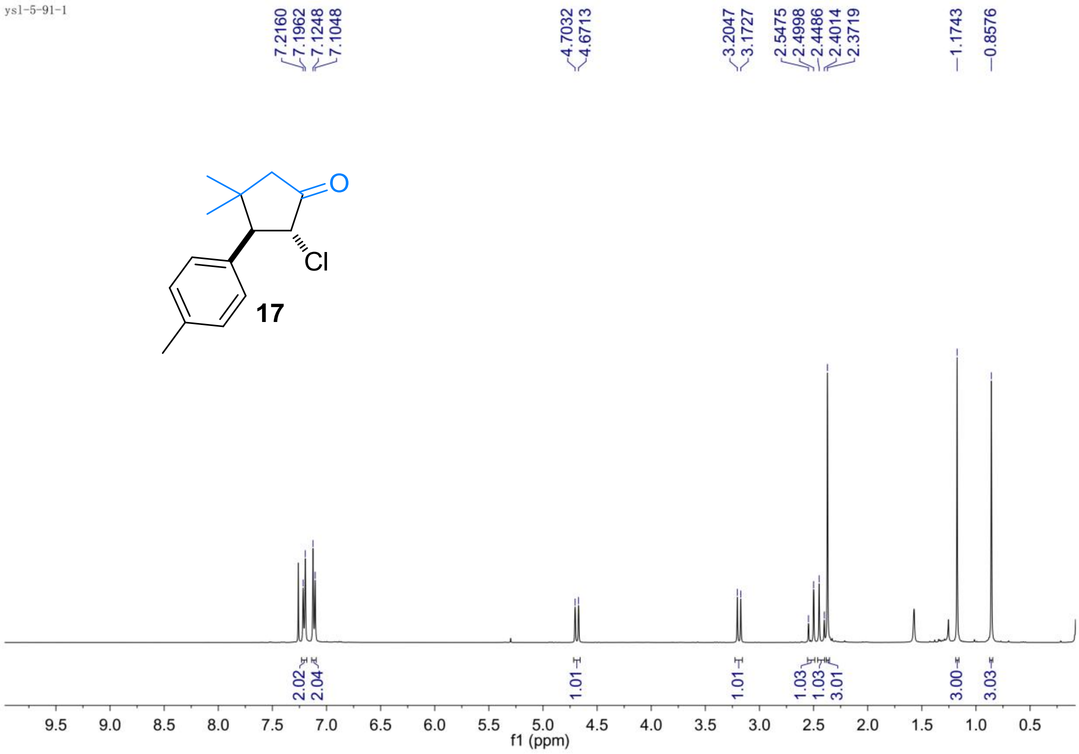

Supplementary Fig. 46. ^{1}H NMR spectrum (400 MHz, $CDCl_3$, 298K) of **17.**

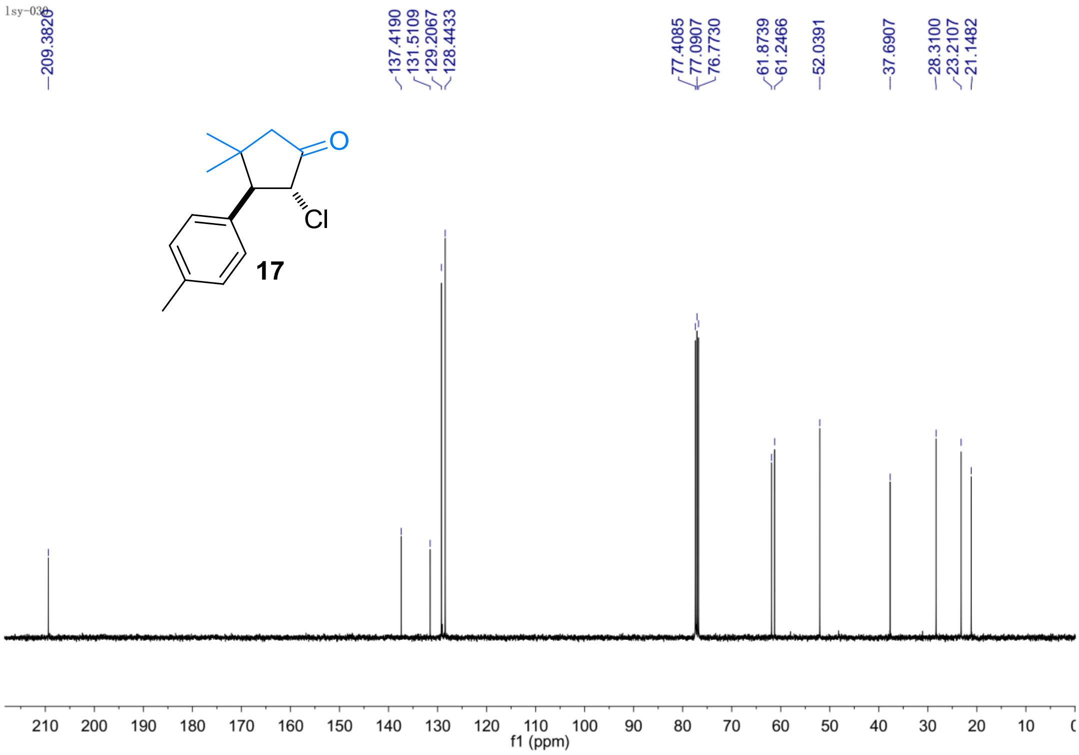

Supplementary Fig. 47. ^{13}C NMR spectrum (151 MHz, $CDCl_3$, 298K) of **17.**

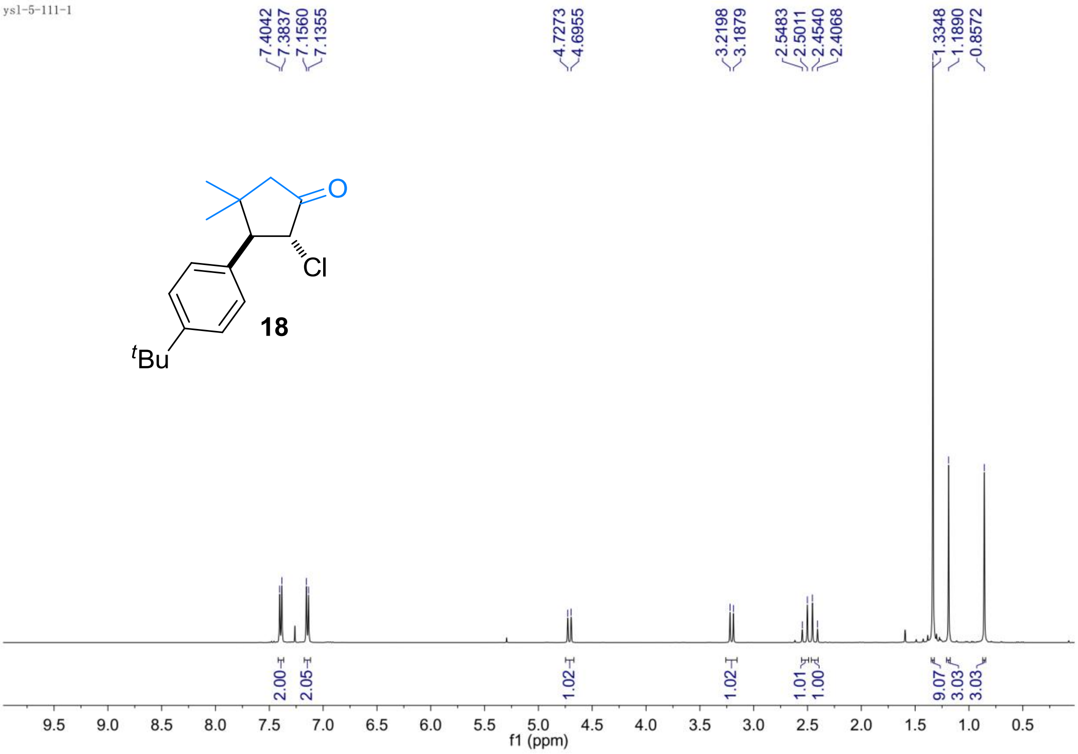

Supplementary Fig. 48. ^{1}H NMR spectrum (400 MHz, $CDCl_3$, 298K) of **18.**

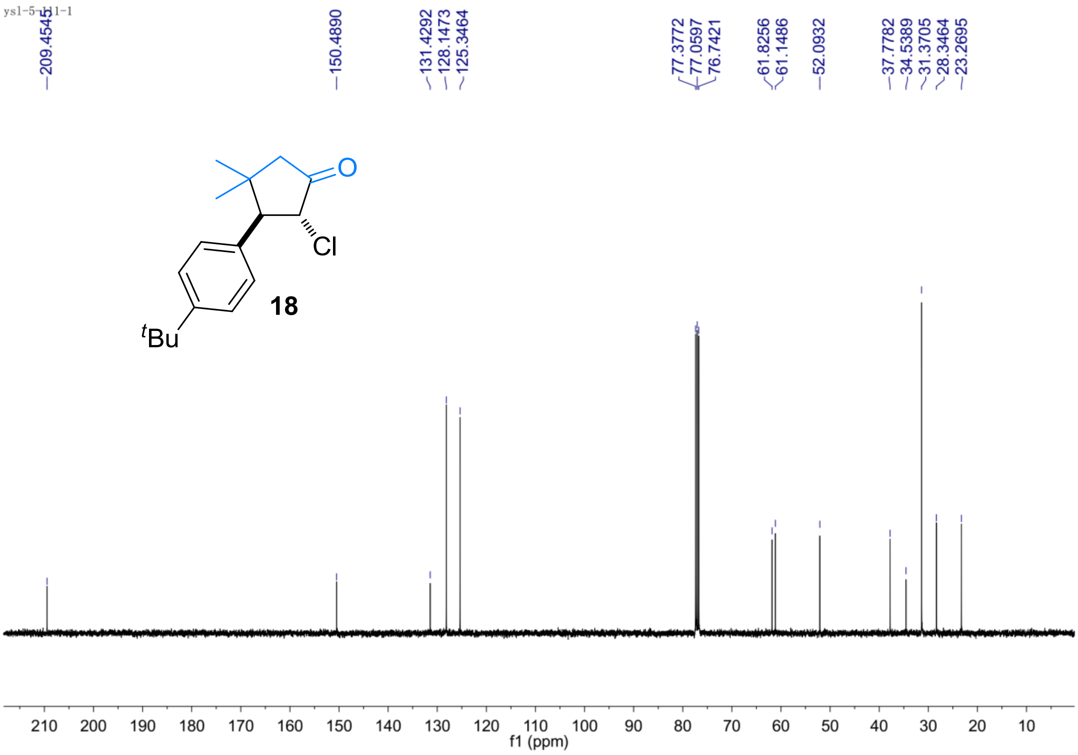

Supplementary Fig. 49. ^{13}C NMR spectrum (101 MHz, $CDCl_3$, 298K) of **18.**

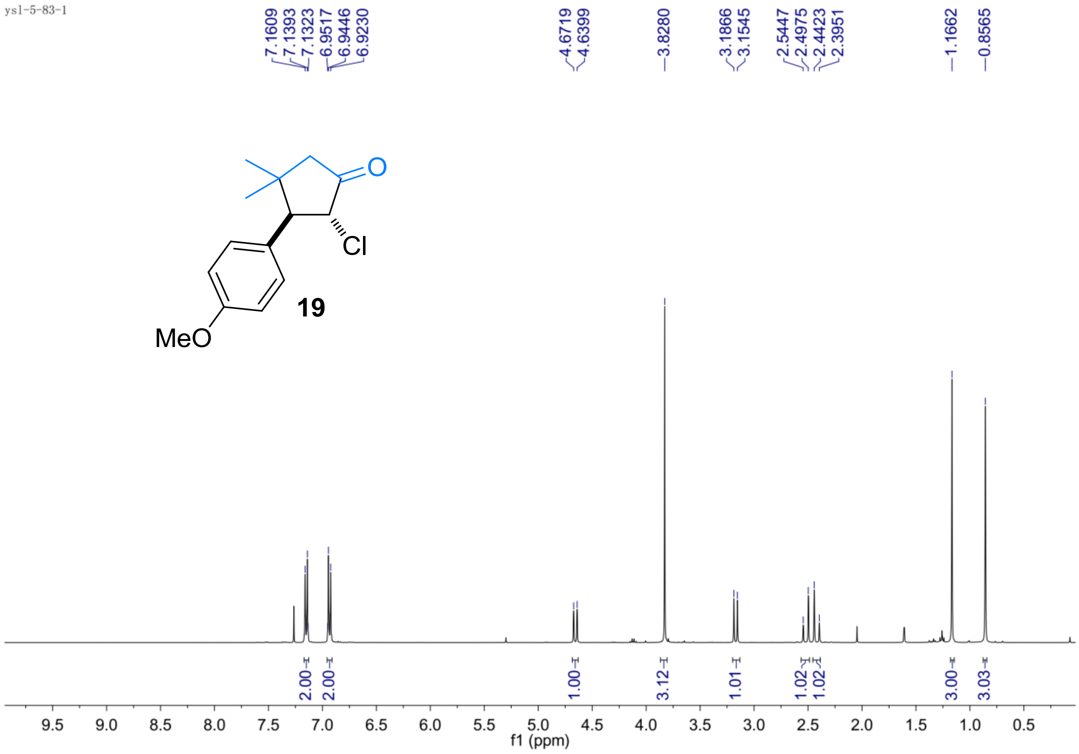

Supplementary Fig. 50. ^{1}H NMR spectrum (400 MHz, $CDCl_3$, 298K) of **19.**

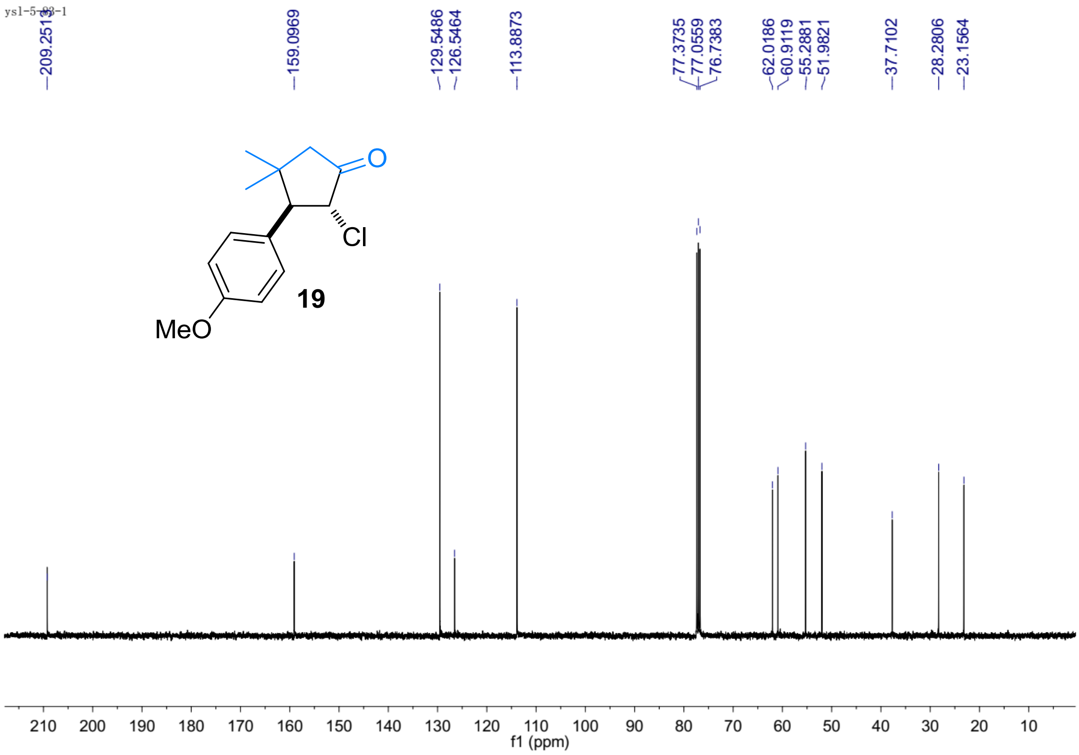

Supplementary Fig. 51. ^{13}C NMR spectrum (101 MHz, $CDCl_3$, 298K) of **19.**

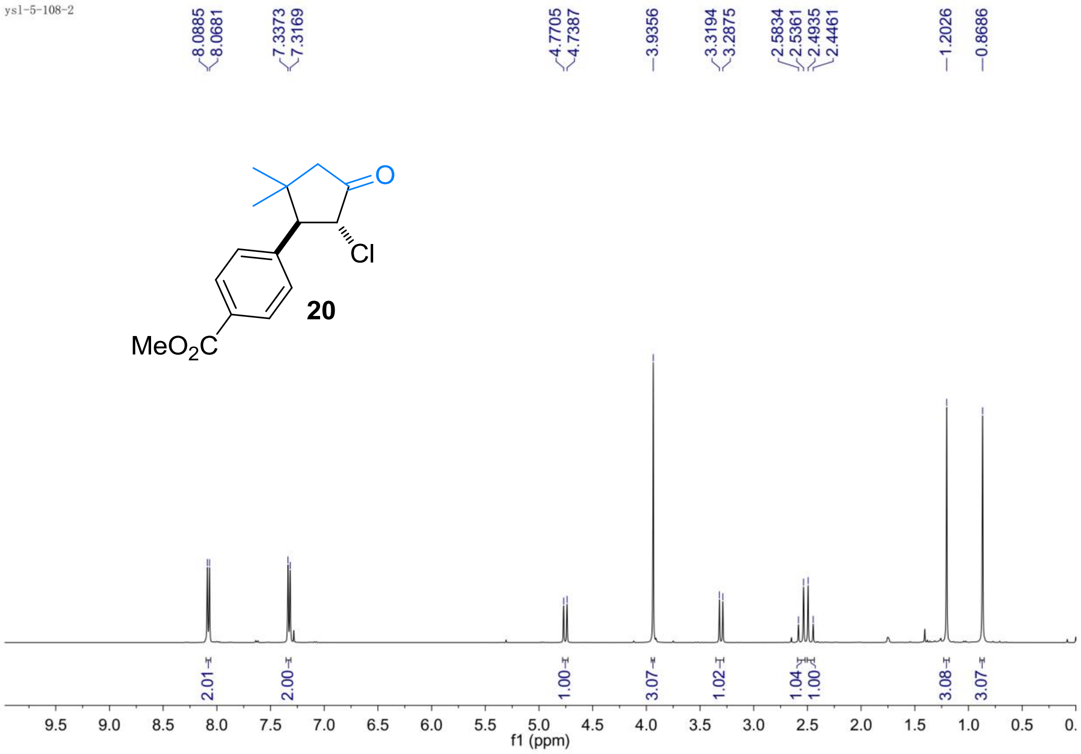

Supplementary Fig. 52. ^{1}H NMR spectrum (400 MHz, $CDCl_3$, 298K) of **20.**

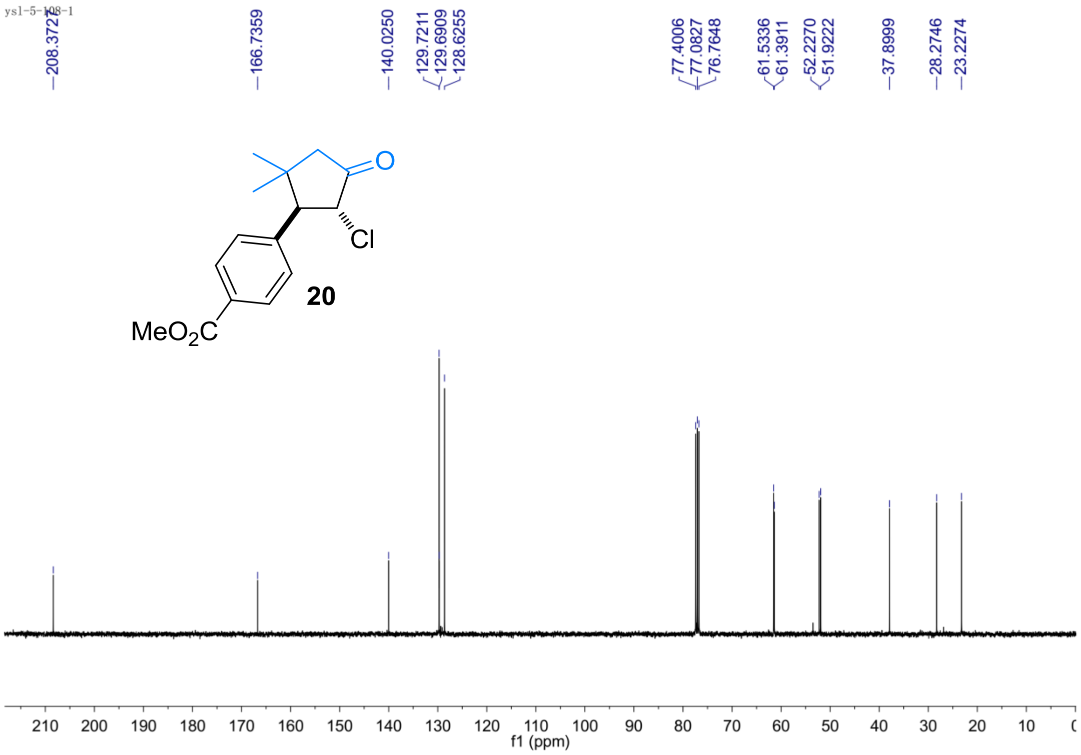

Supplementary Fig. 53. ^{13}C NMR spectrum (101 MHz, $CDCl_3$, 298K) of **20.**

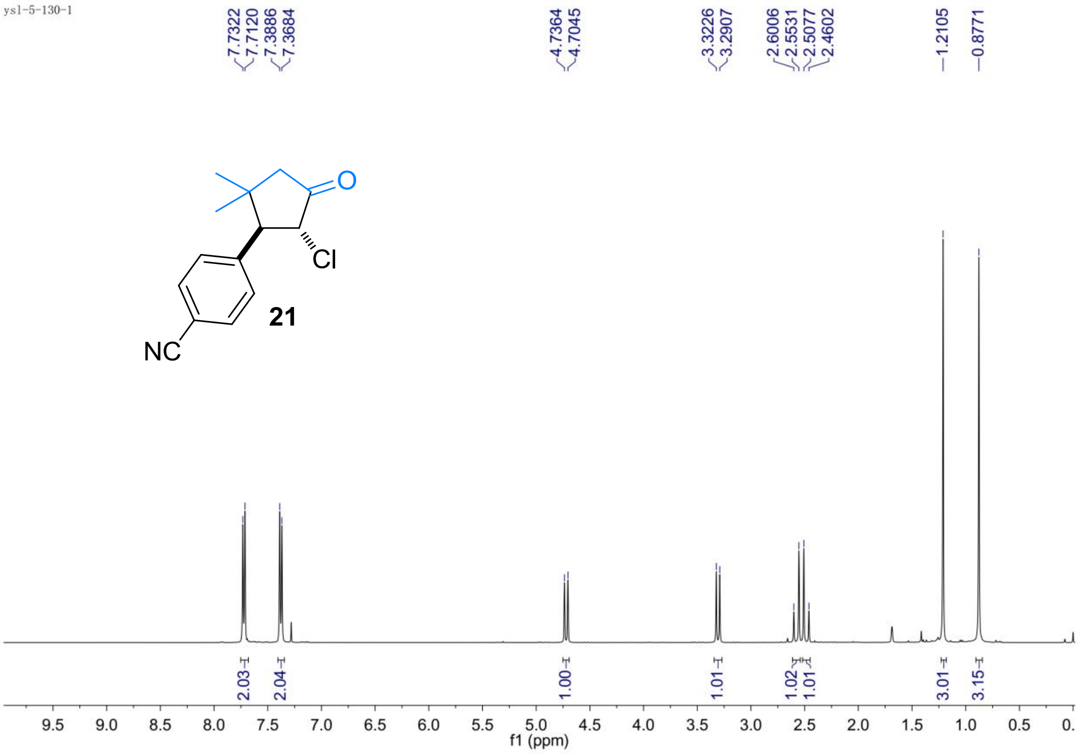

Supplementary Fig. 54. ^{1}H NMR spectrum (400 MHz, $CDCl_3$, 298K) of **21.**

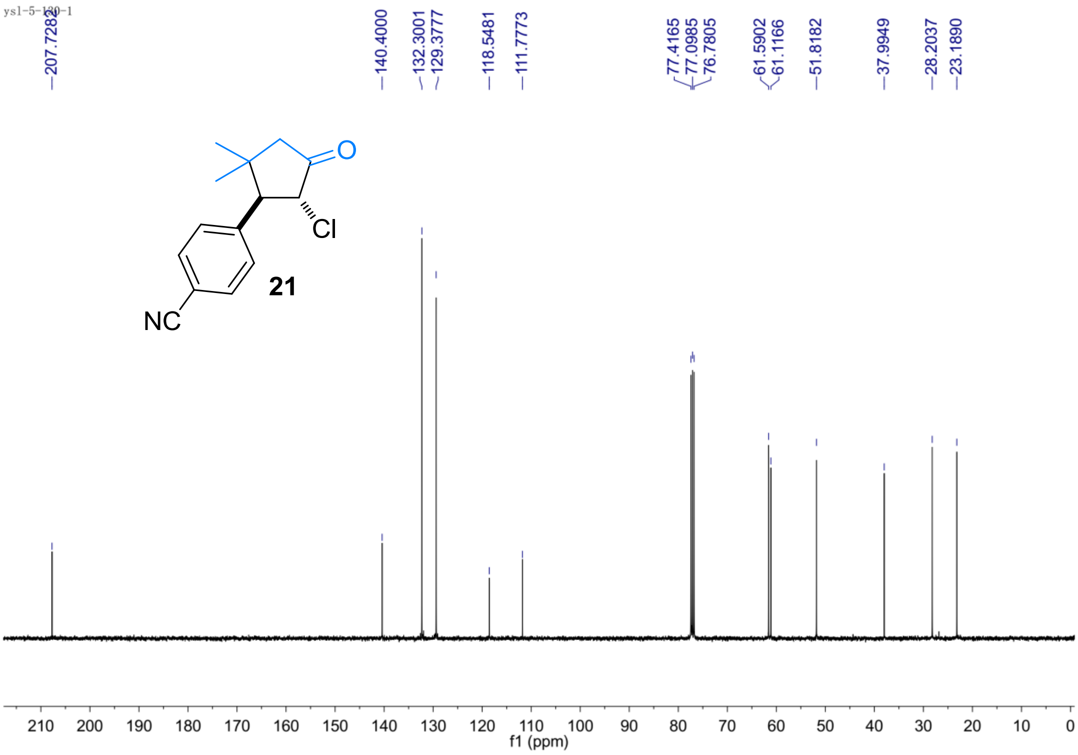

Supplementary Fig. 55. ^{13}C NMR spectrum (101 MHz, $CDCl_3$, 298K) of **21.**

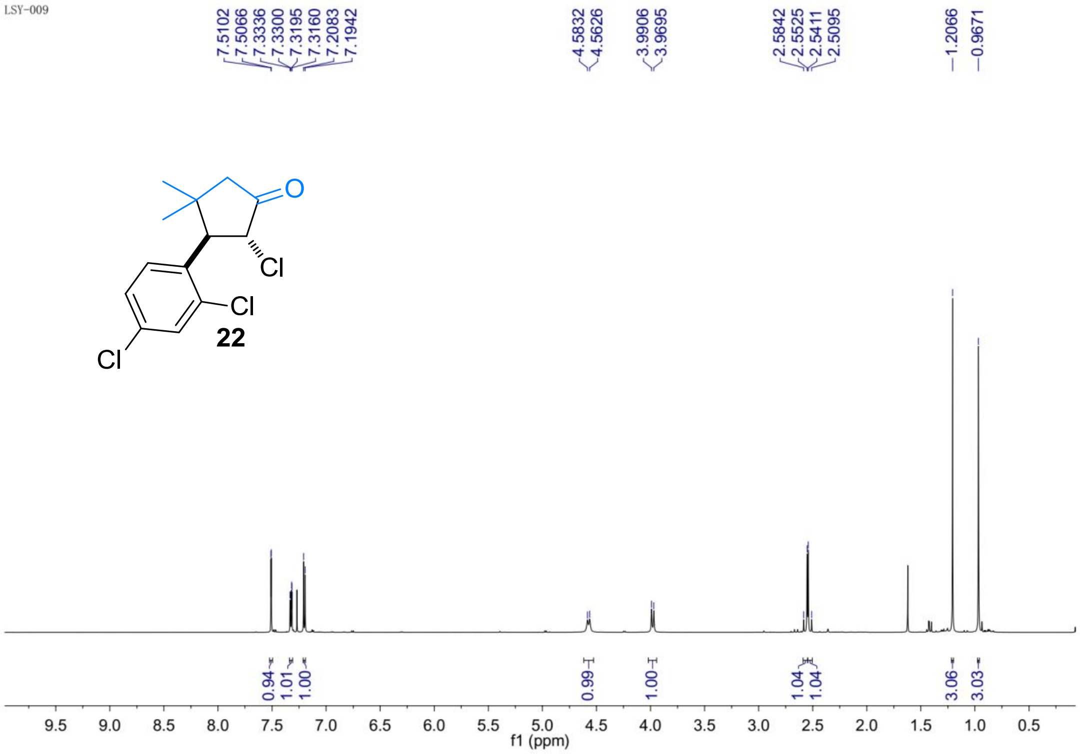

Supplementary Fig. 56. ^{1}H NMR spectrum (600 MHz, $CDCl_3$, 298K) of **22.**

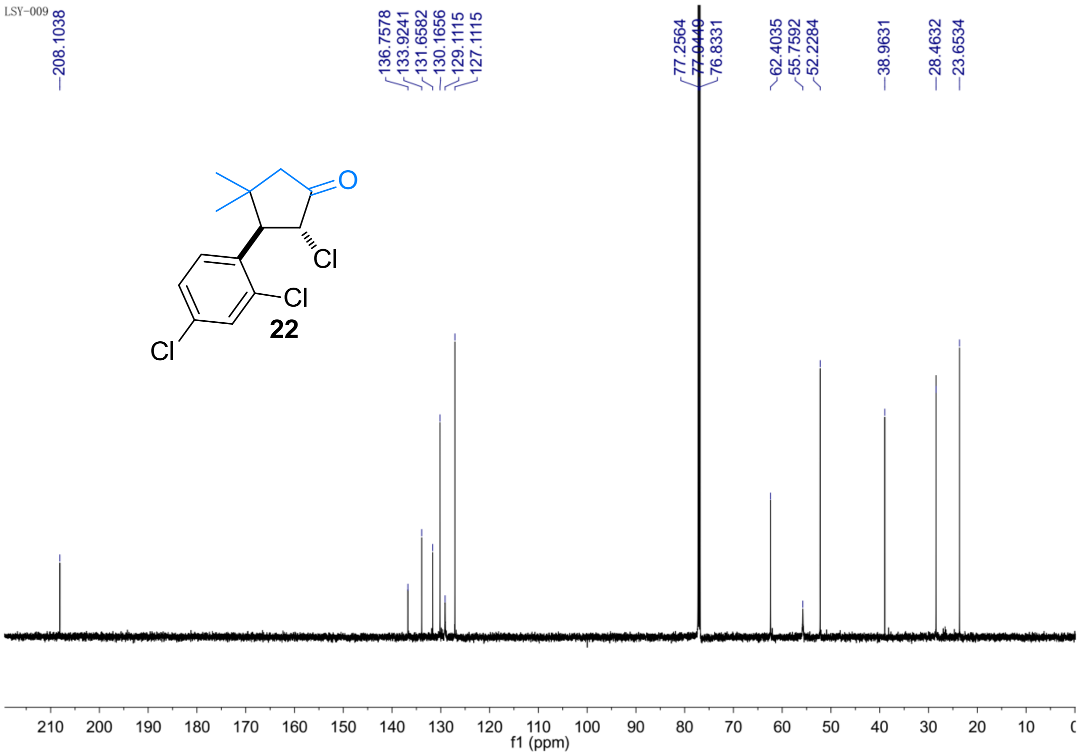

Supplementary Fig. 57. ^{13}C NMR spectrum (151 MHz, $CDCl_3$, 298K) of **22.**

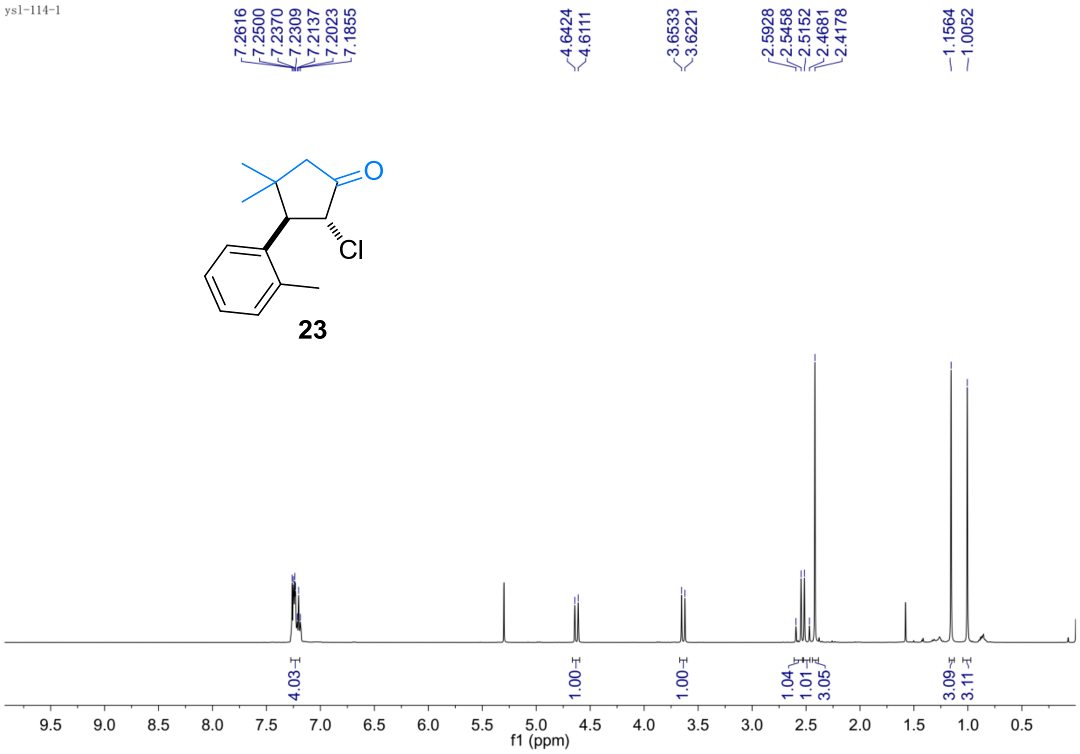

Supplementary Fig. 58. ^{1}H NMR spectrum (400 MHz, $CDCl_3$, 298K) of **23.**

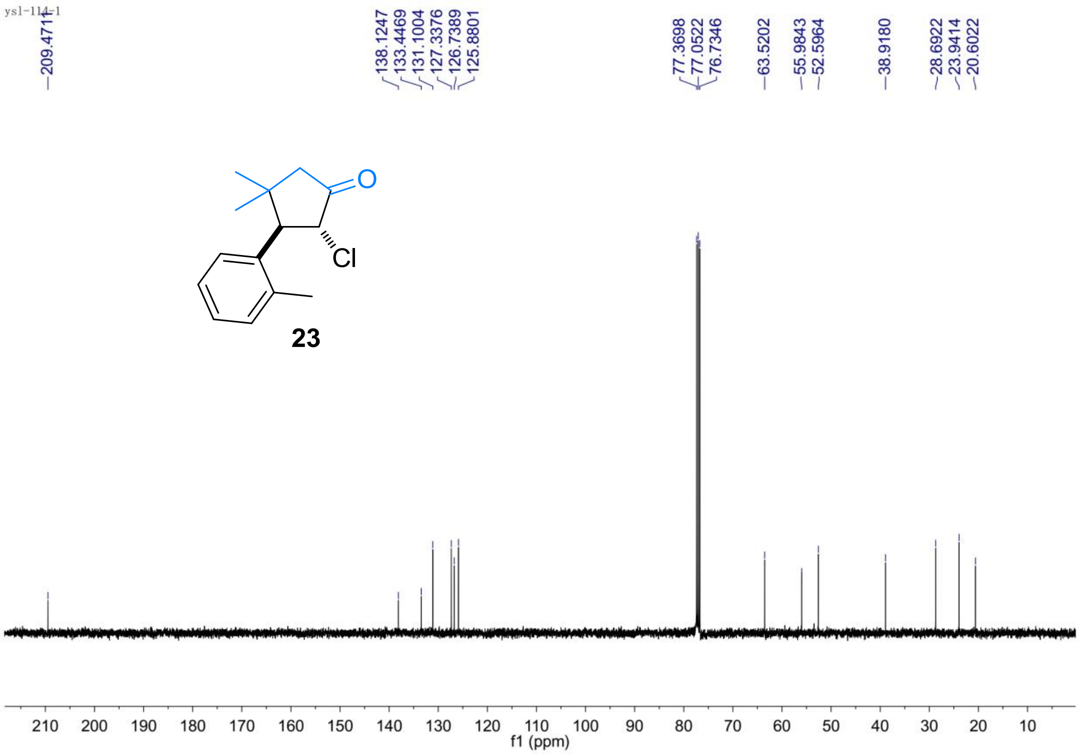

Supplementary Fig. 59. ^{13}C NMR spectrum (101 MHz, $CDCl_3$, 298K) of **23.**

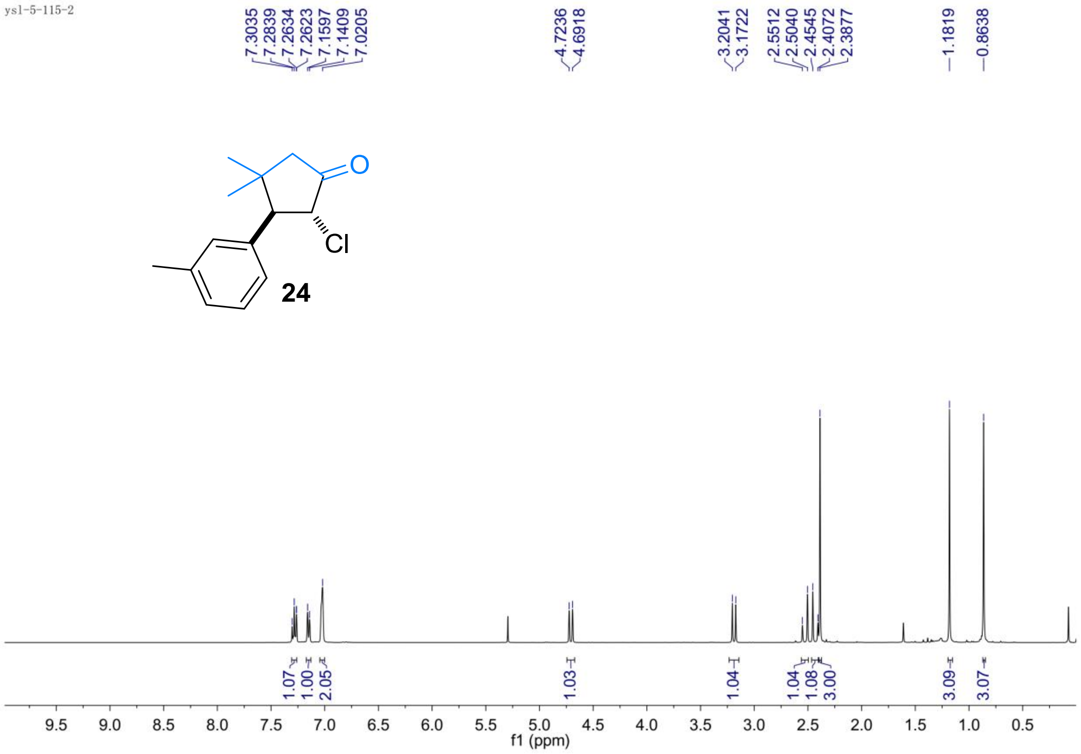

Supplementary Fig. 60. ^{1}H NMR spectrum (400 MHz, $CDCl_3$, 298K) of **24.**

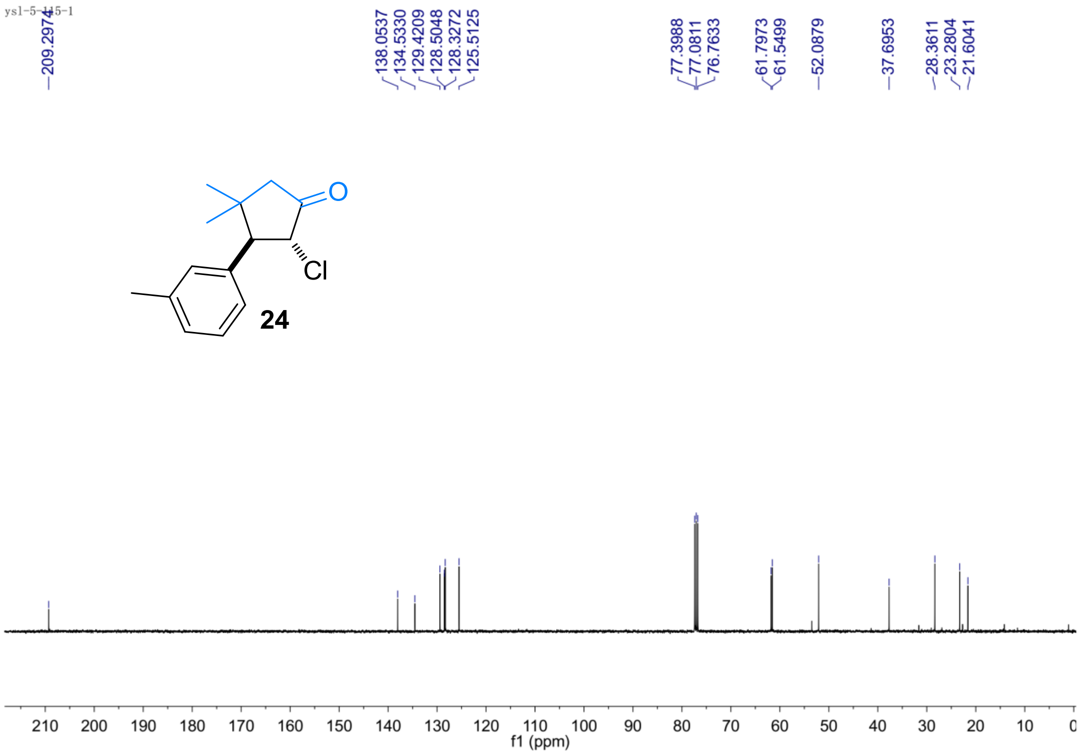

Supplementary Fig. 61. ^{13}C NMR spectrum (101 MHz, $CDCl_3$, 298K) of **24.**

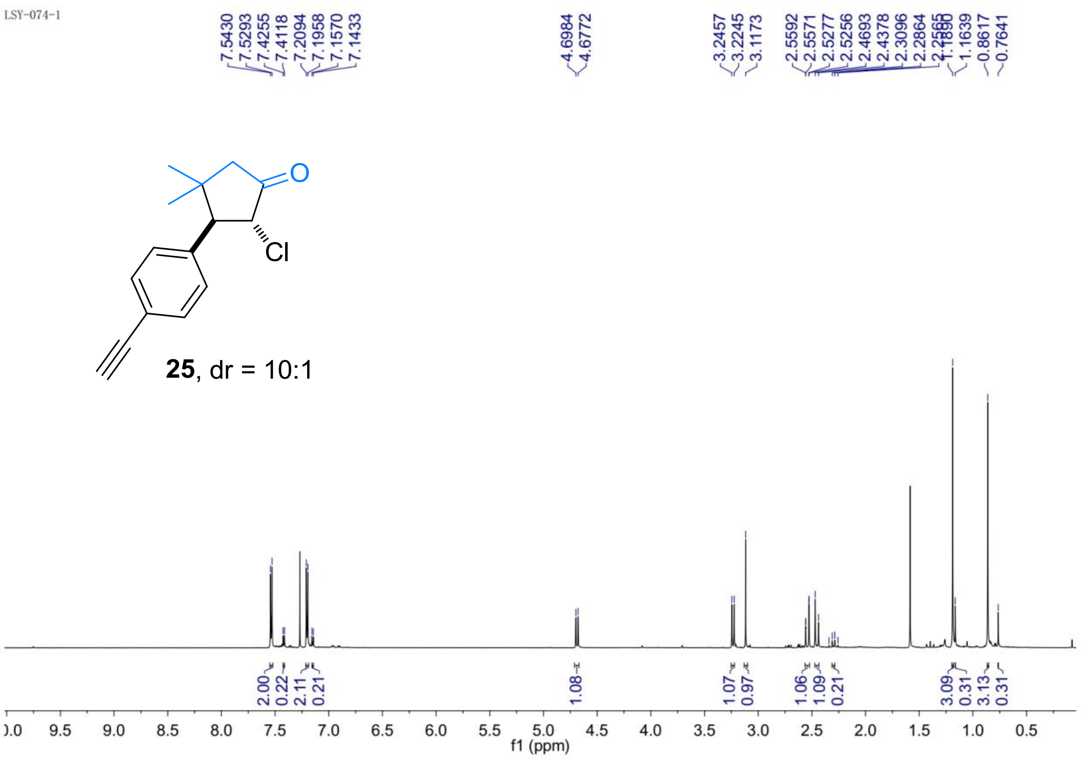

Supplementary Fig. 62. ^{1}H NMR spectrum (600 MHz, $CDCl_3$, 298K) of **25.**

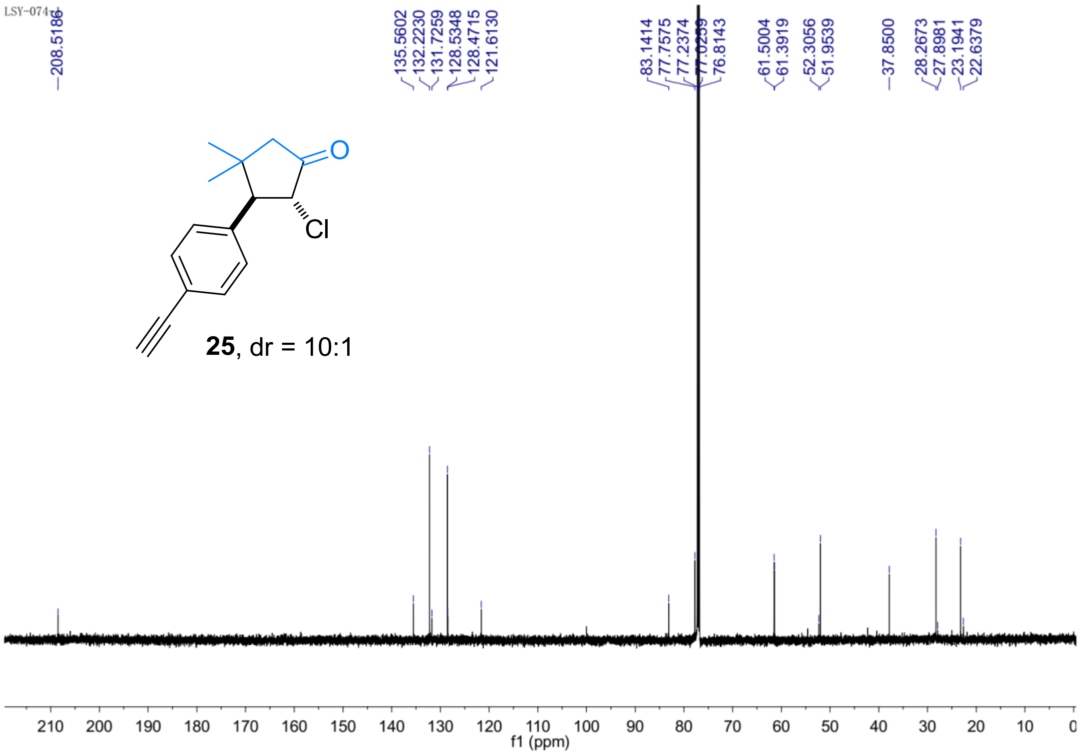

Supplementary Fig. 63. ^{13}C NMR spectrum (151 MHz, $CDCl_3$, 298K) of **25.**

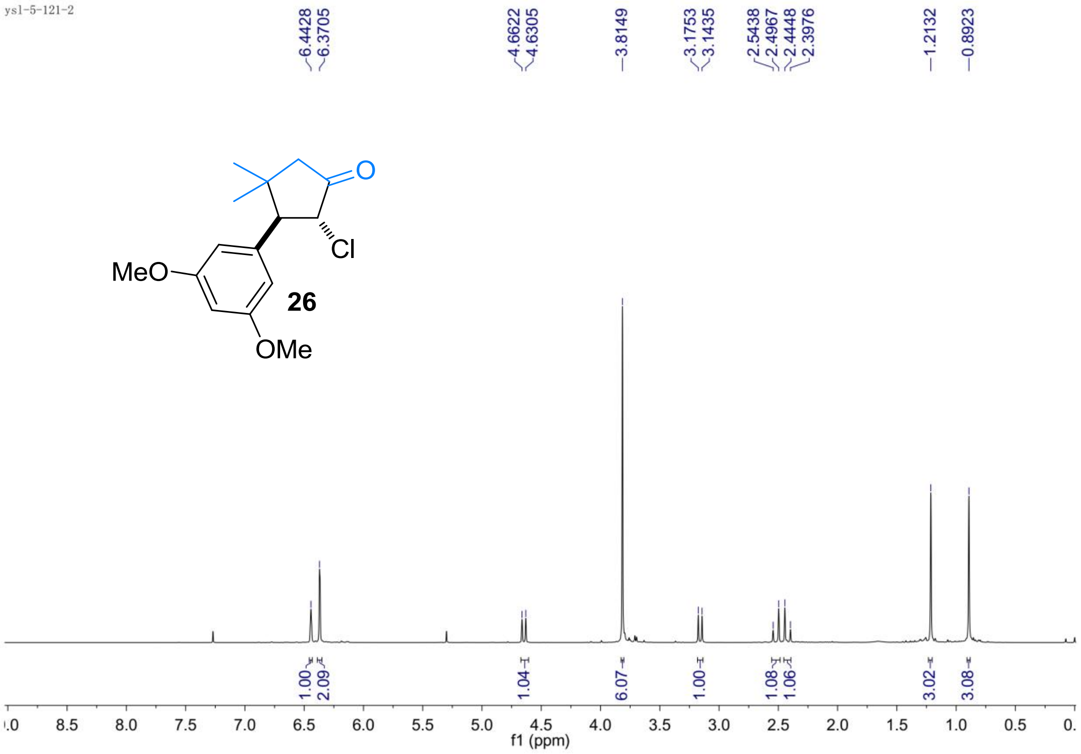

Supplementary Fig. 64. ^{1}H NMR spectrum (400 MHz, $CDCl_3$, 298K) of **26.**

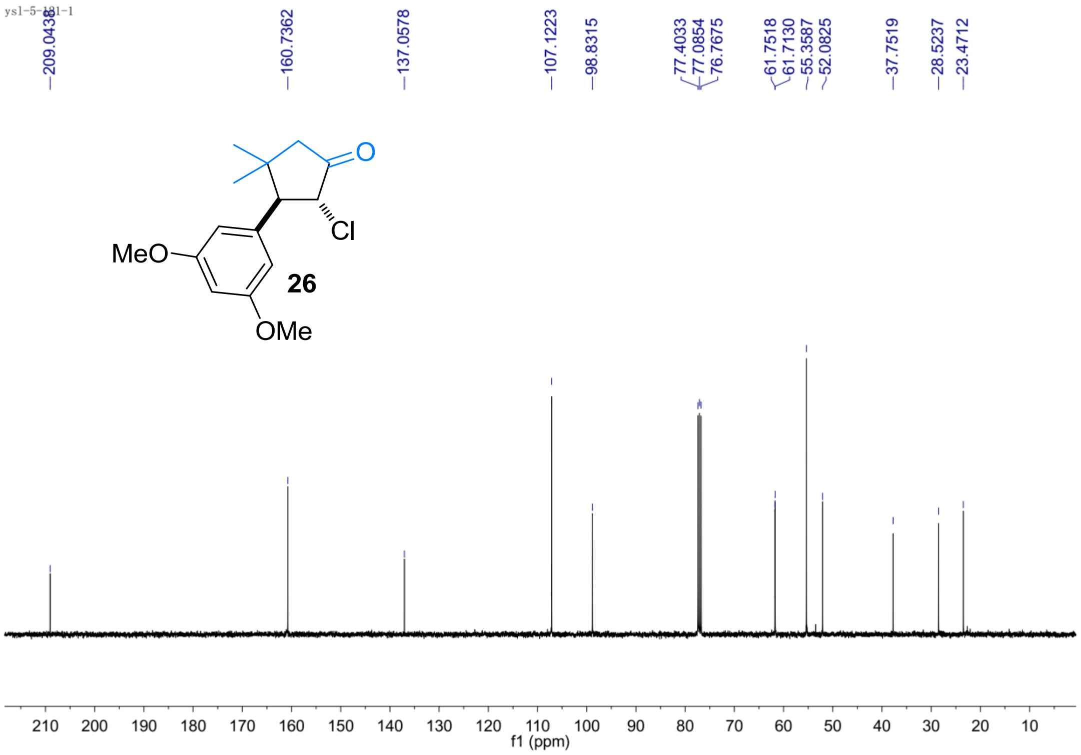

Supplementary Fig. 65. ^{13}C NMR spectrum (101 MHz, $CDCl_3$, 298K) of **26.**

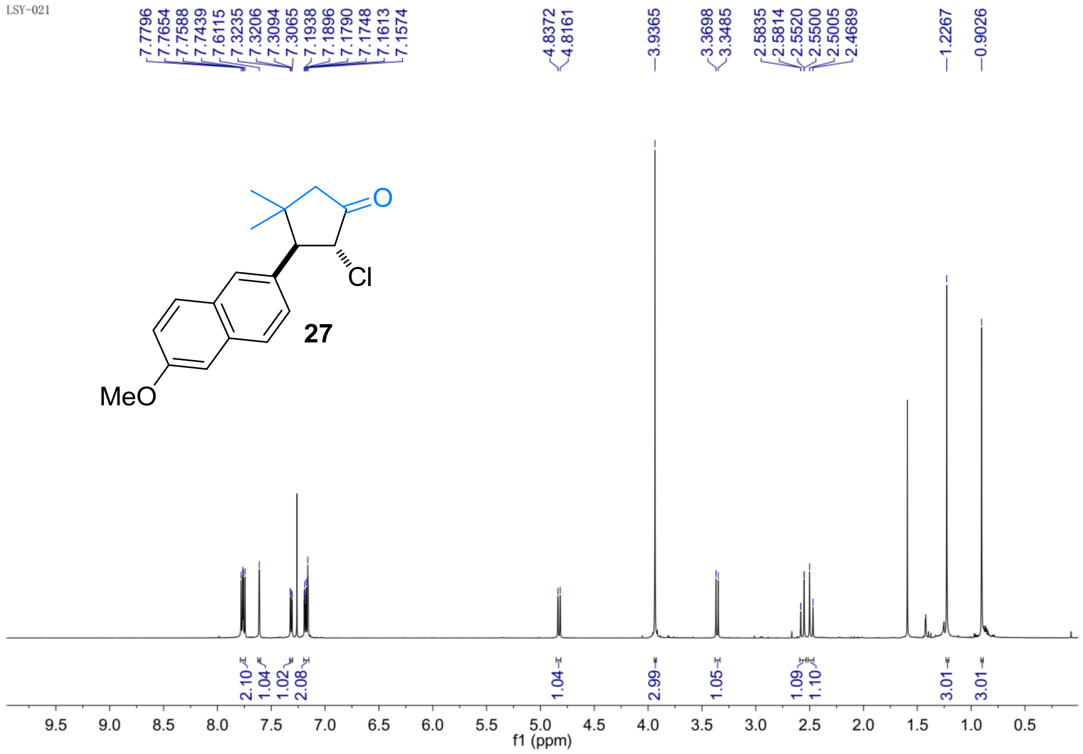

Supplementary Fig. 66. ^{1}H NMR spectrum (600 MHz, $CDCl_3$, 298K) of **27.**

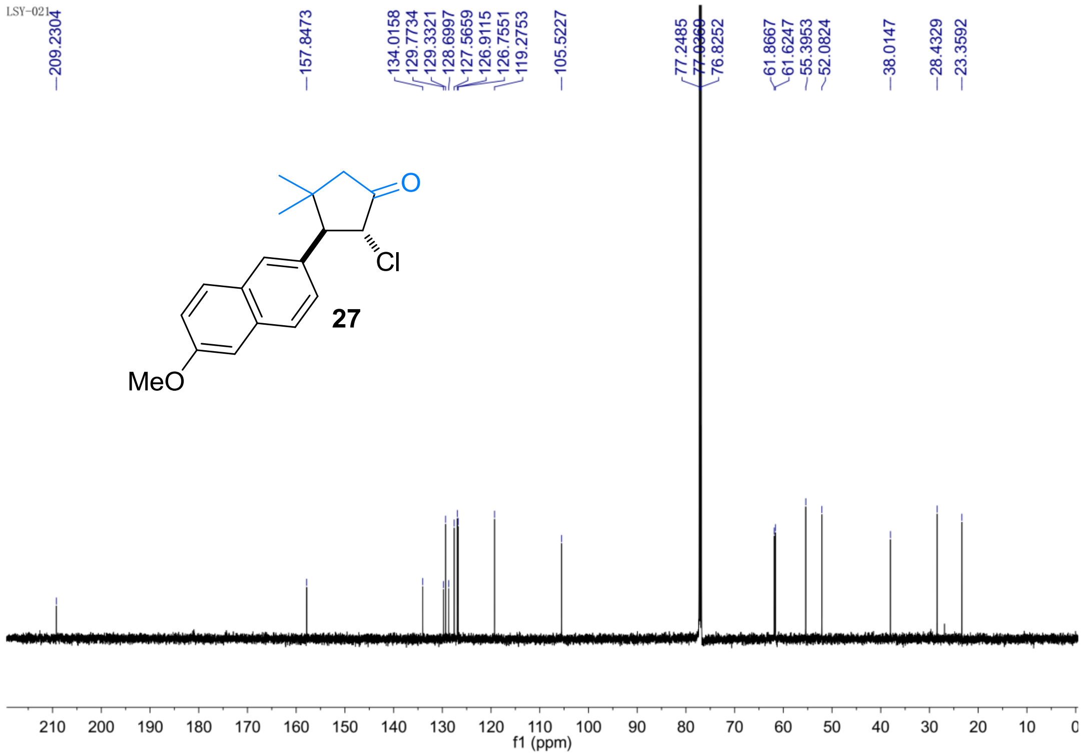

Supplementary Fig. 67. ^{13}C NMR spectrum (151 MHz, $CDCl_3$, 298K) of **27.**

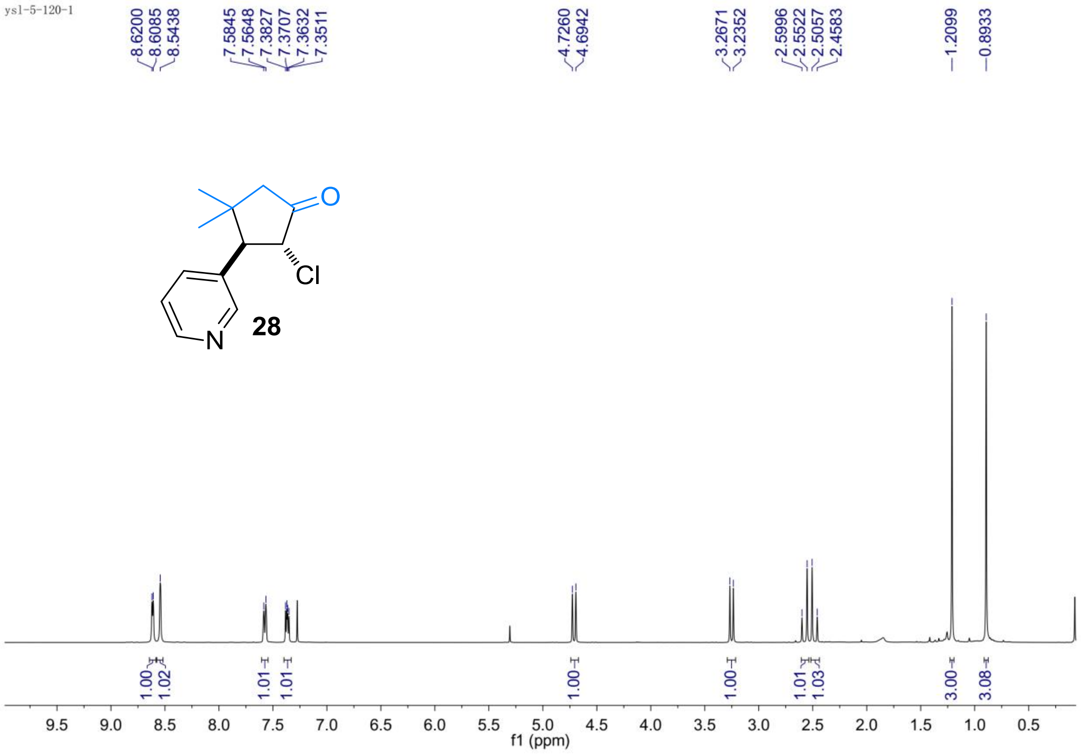

Supplementary Fig. 68. ^{1}H NMR spectrum (400 MHz, $CDCl_3$, 298K) of **28.**

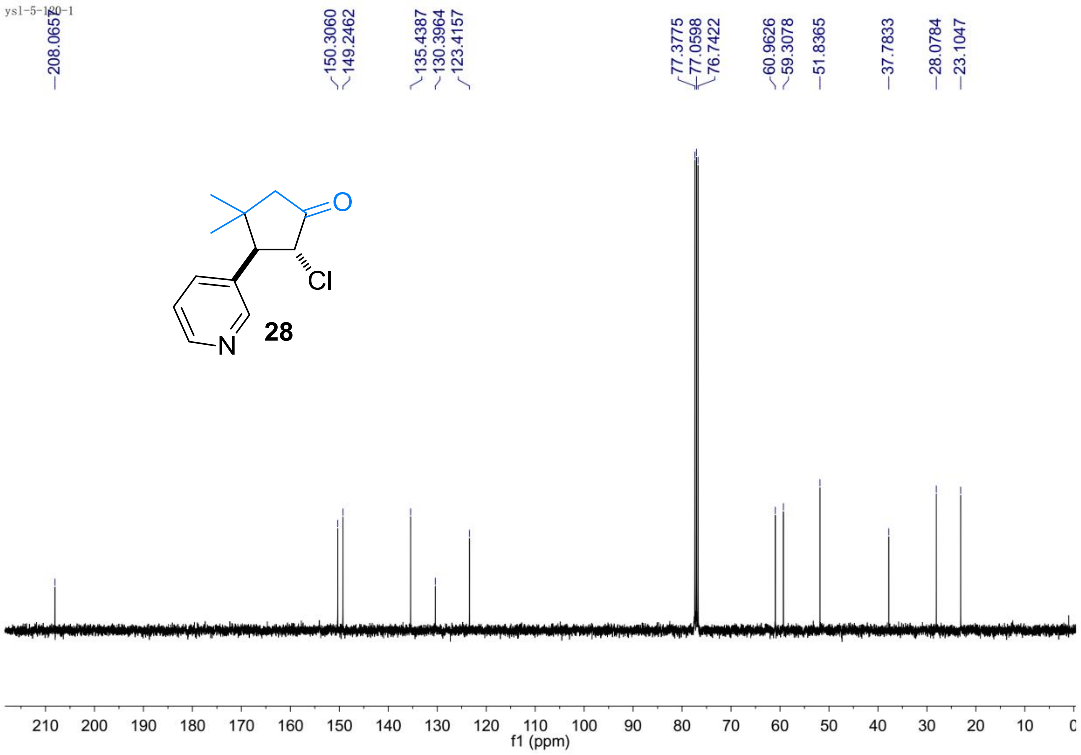

Supplementary Fig. 69. ^{13}C NMR spectrum (101 MHz, $CDCl_3$, 298K) of **28.**

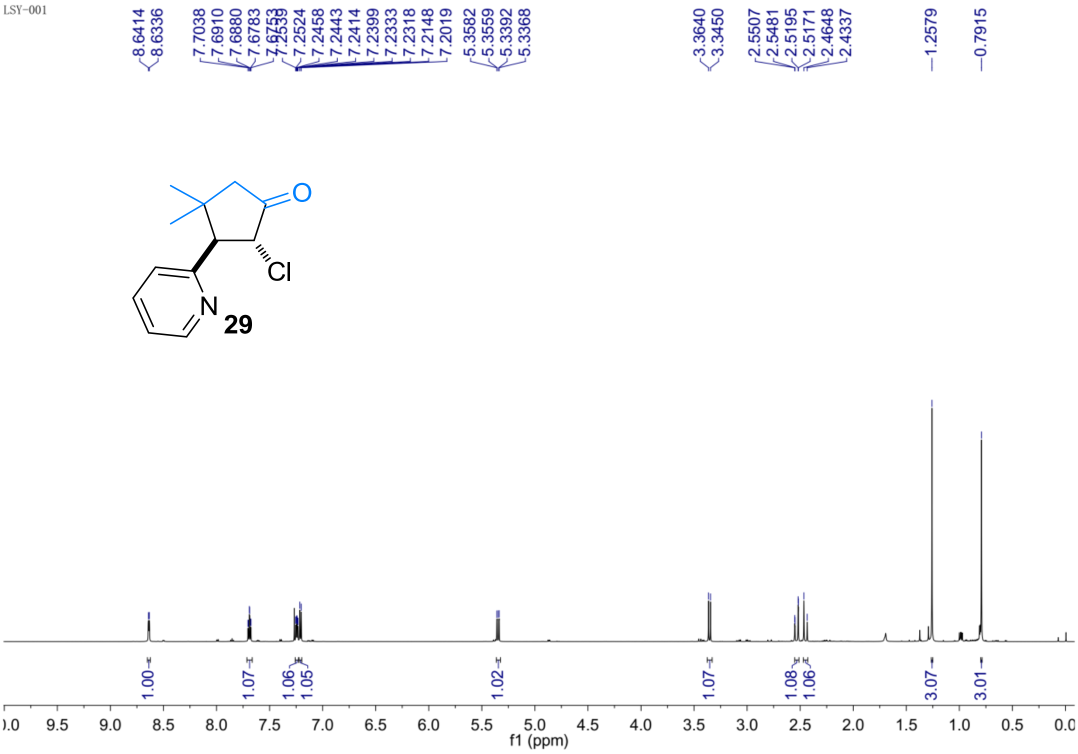

Supplementary Fig. 70. ^{1}H NMR spectrum (600 MHz, $CDCl_3$, 298K) of **29.**

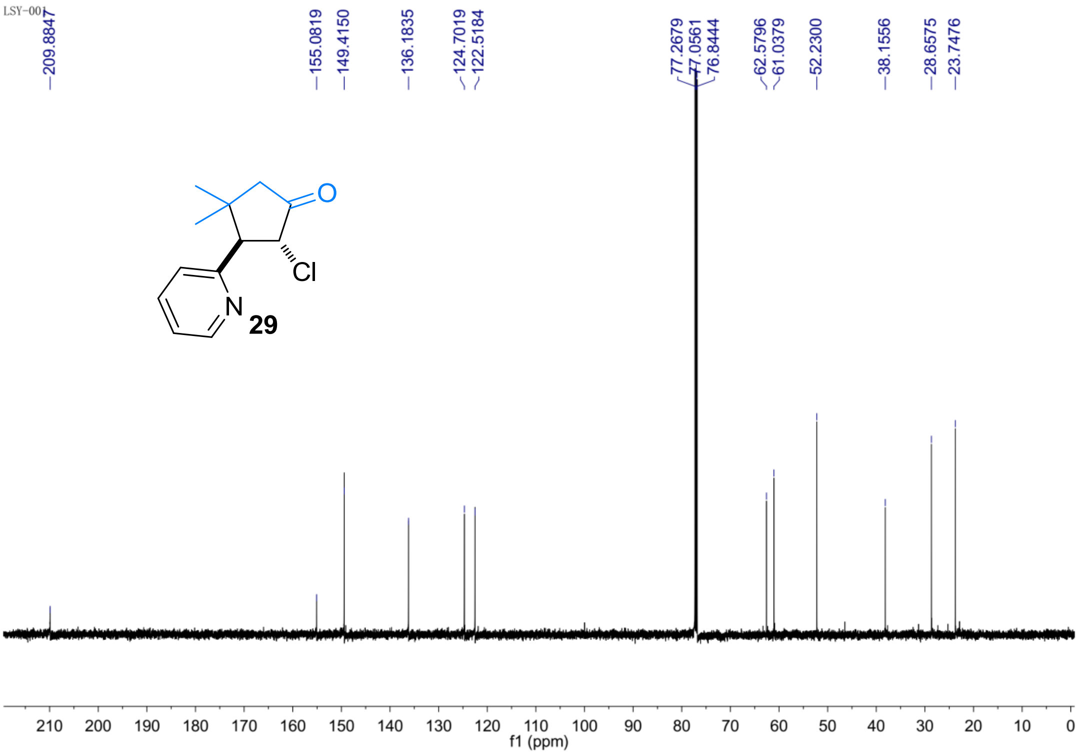

Supplementary Fig. 71. ^{13}C NMR spectrum (151 MHz, $CDCl_3$, 298K) of **29.**

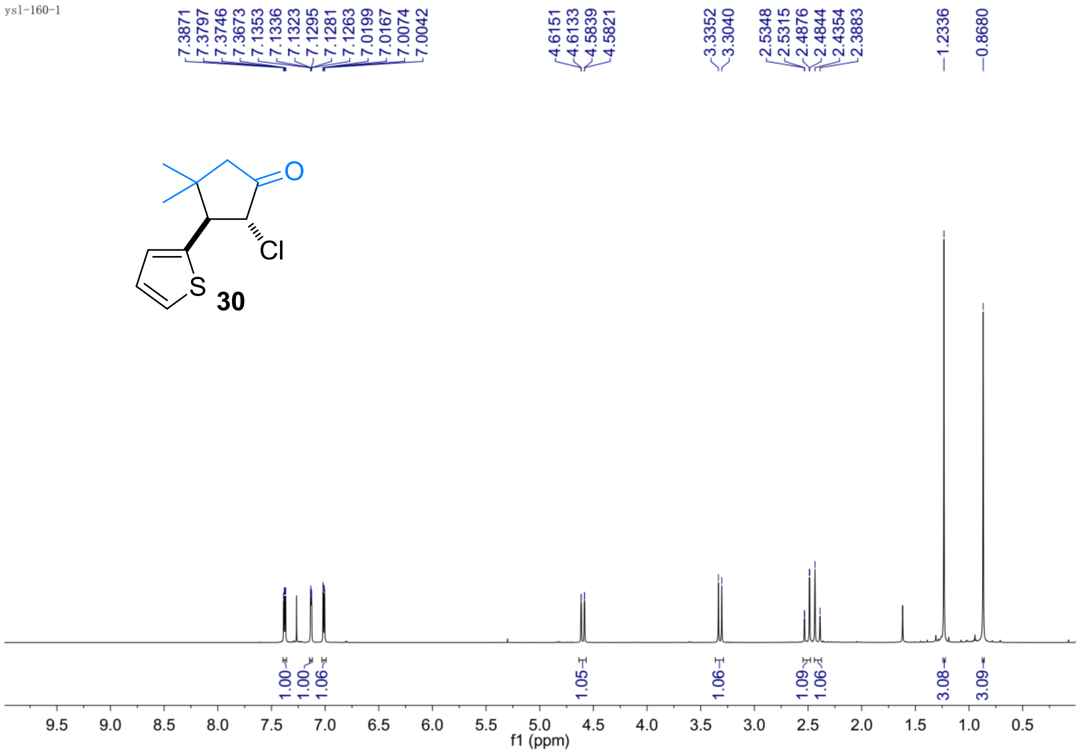

Supplementary Fig. 72. ^{1}H NMR spectrum (400 MHz, $CDCl_3$, 298K) of **30.**

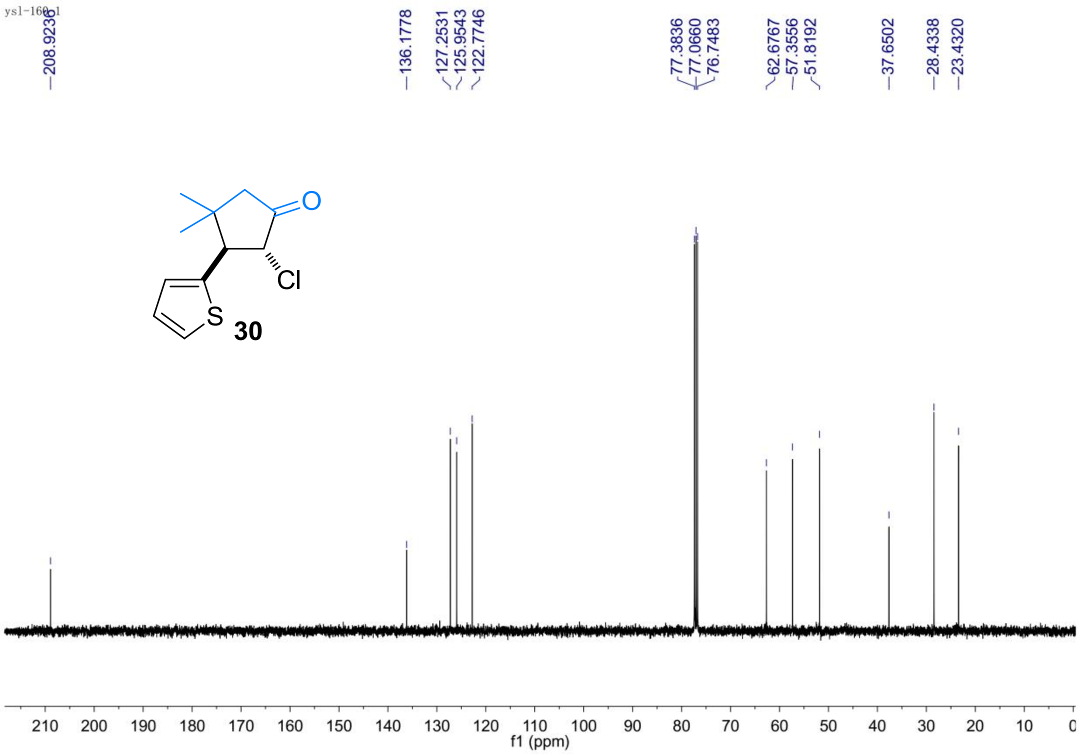

Supplementary Fig. 73. ^{13}C NMR spectrum (101 MHz, $CDCl_3$, 298K) of **30.**

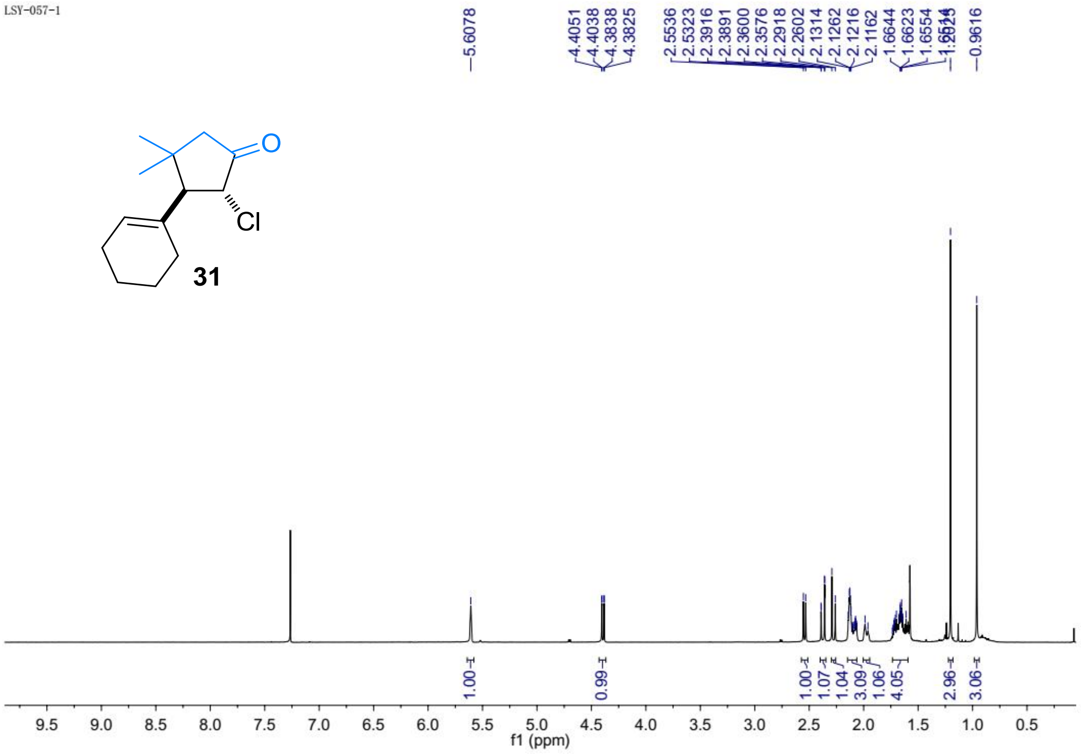

Supplementary Fig. 74. ^{1}H NMR spectrum (600 MHz, $CDCl_3$, 298K) of **31.**

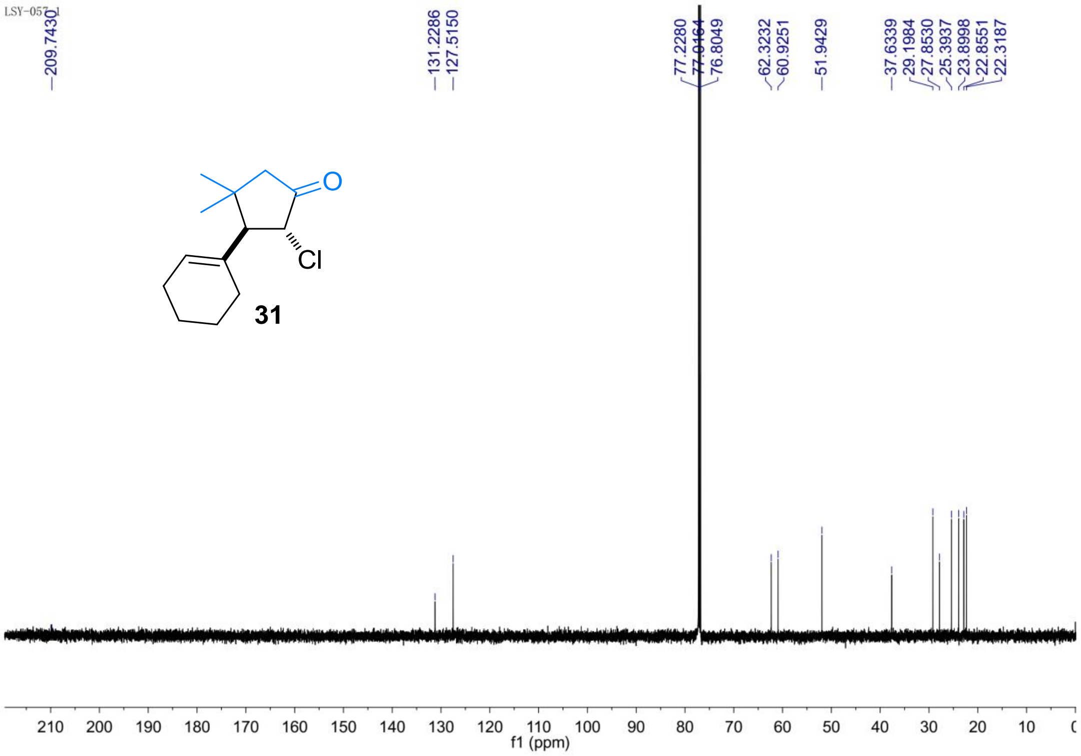

Supplementary Fig. 75. ^{13}C NMR spectrum (151 MHz, $CDCl_3$, 298K) of **31.**

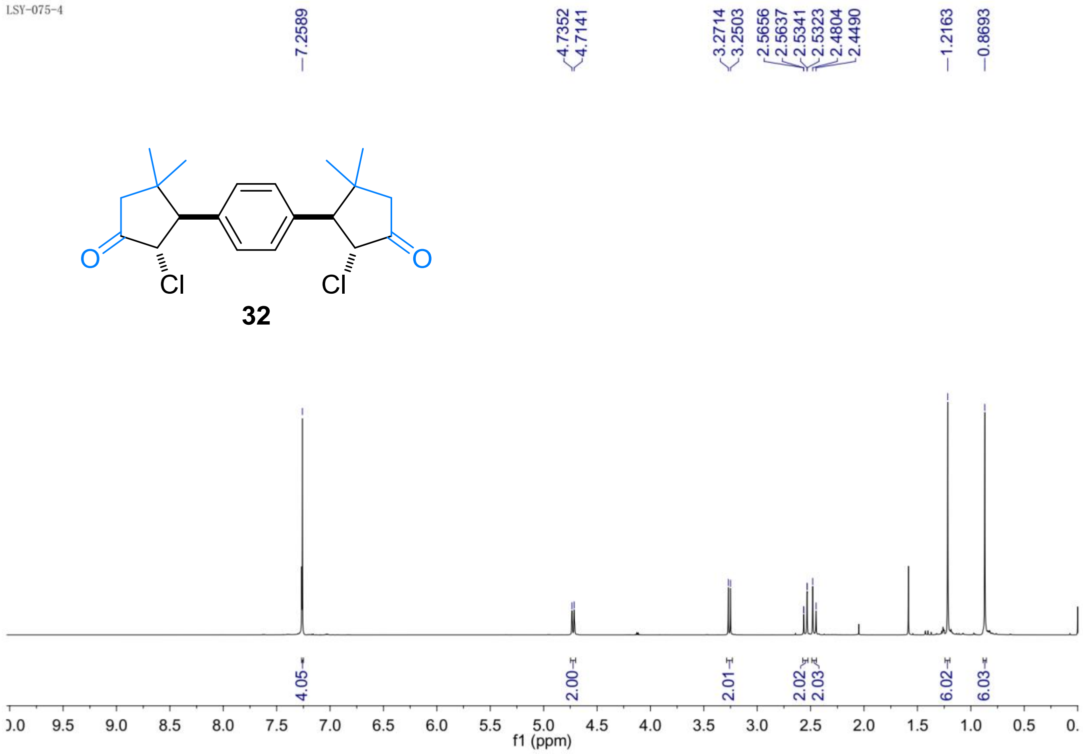

Supplementary Fig. 76. ^{1}H NMR spectrum (600 MHz, $CDCl_3$, 298K) of **32.**

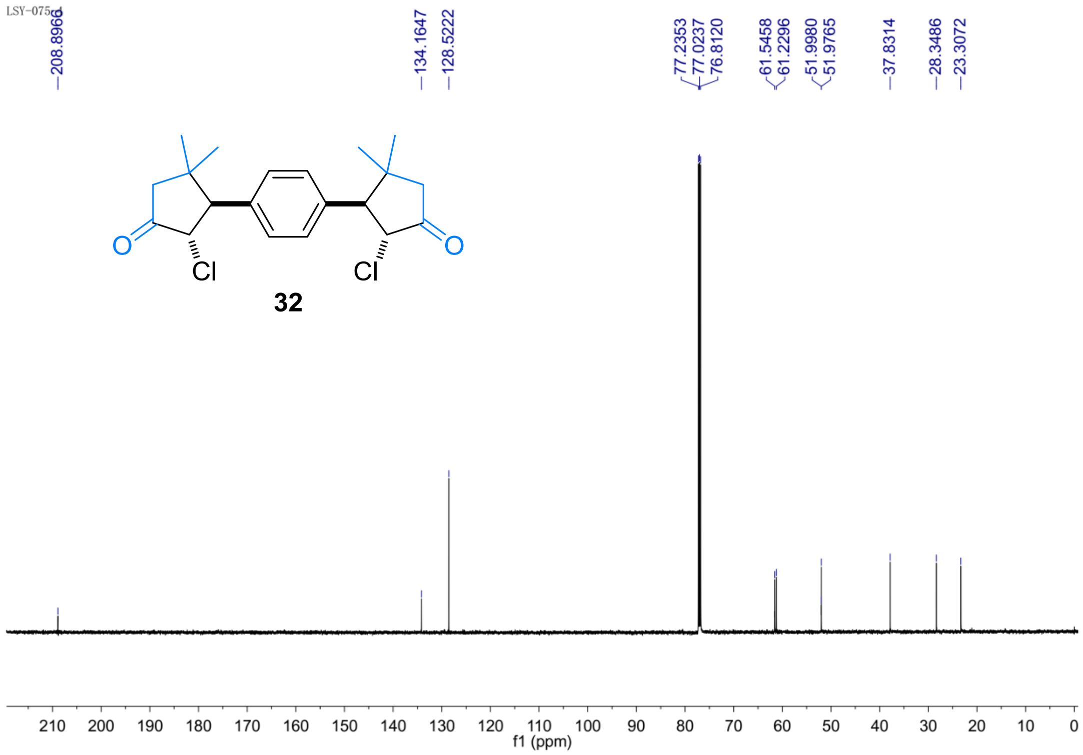

Supplementary Fig. 77. ^{13}C NMR spectrum (151 MHz, $CDCl_3$, 298K) of **32.**

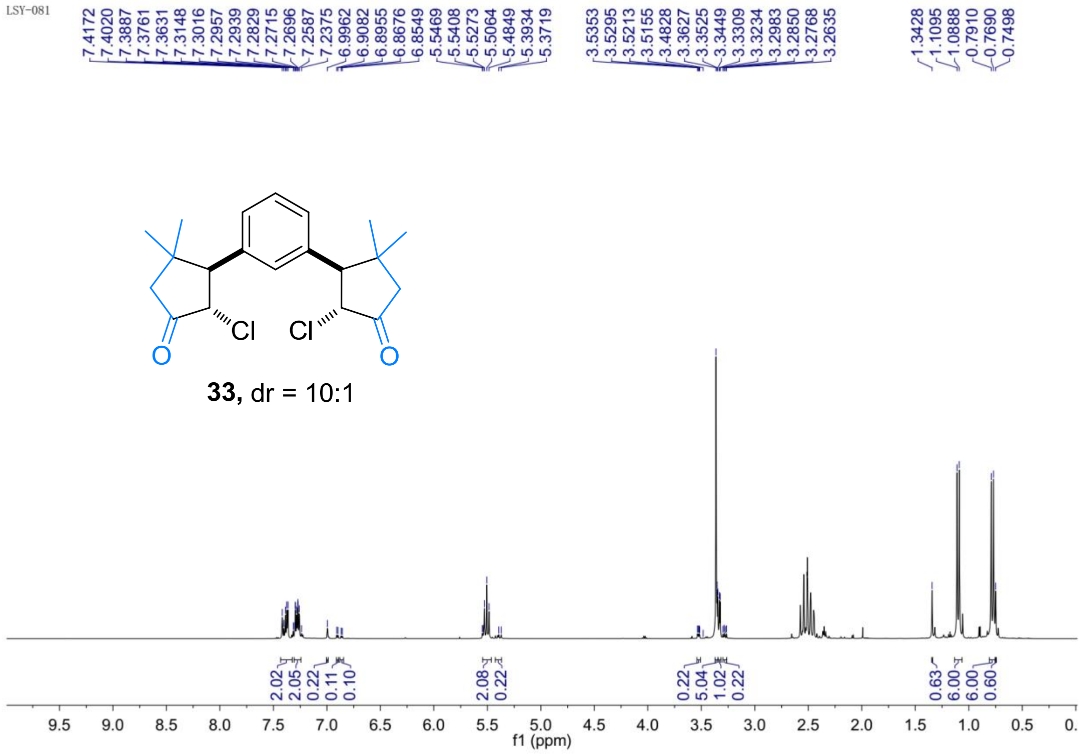

Supplementary Fig. 78. ^{1}H NMR spectrum (600 MHz, d^6-DMSO, 298K) of **33.**

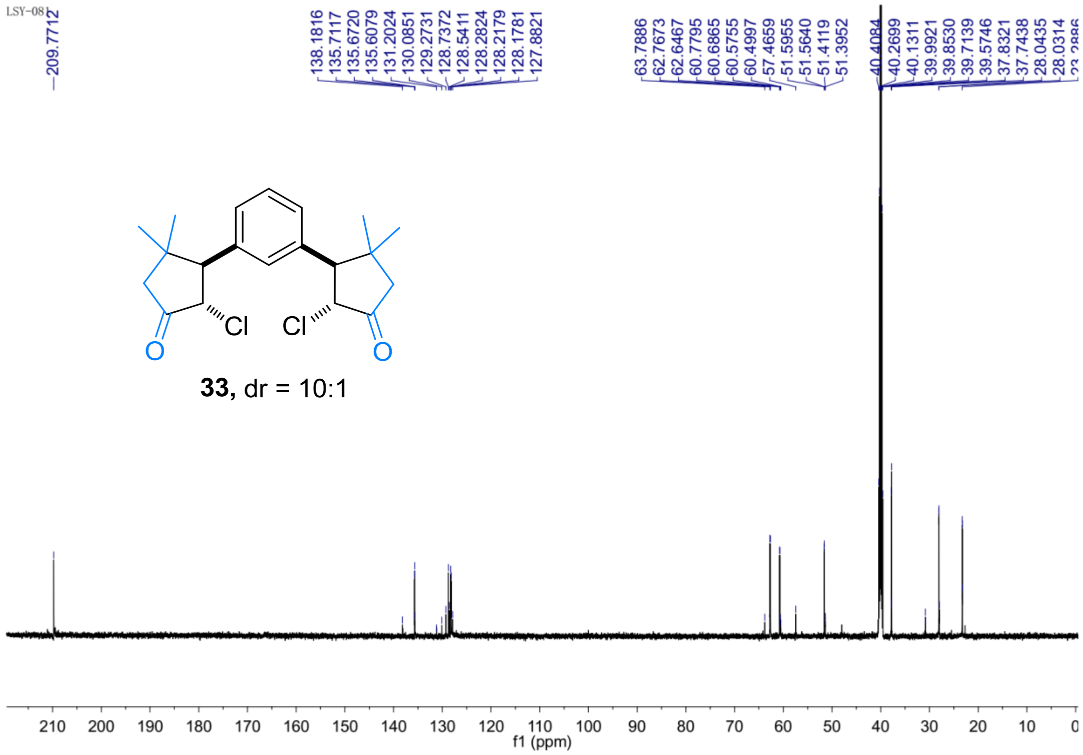

Supplementary Fig. 79. ^{13}C NMR spectrum (151 MHz, d^6-DMSO, 298K) of **33.**

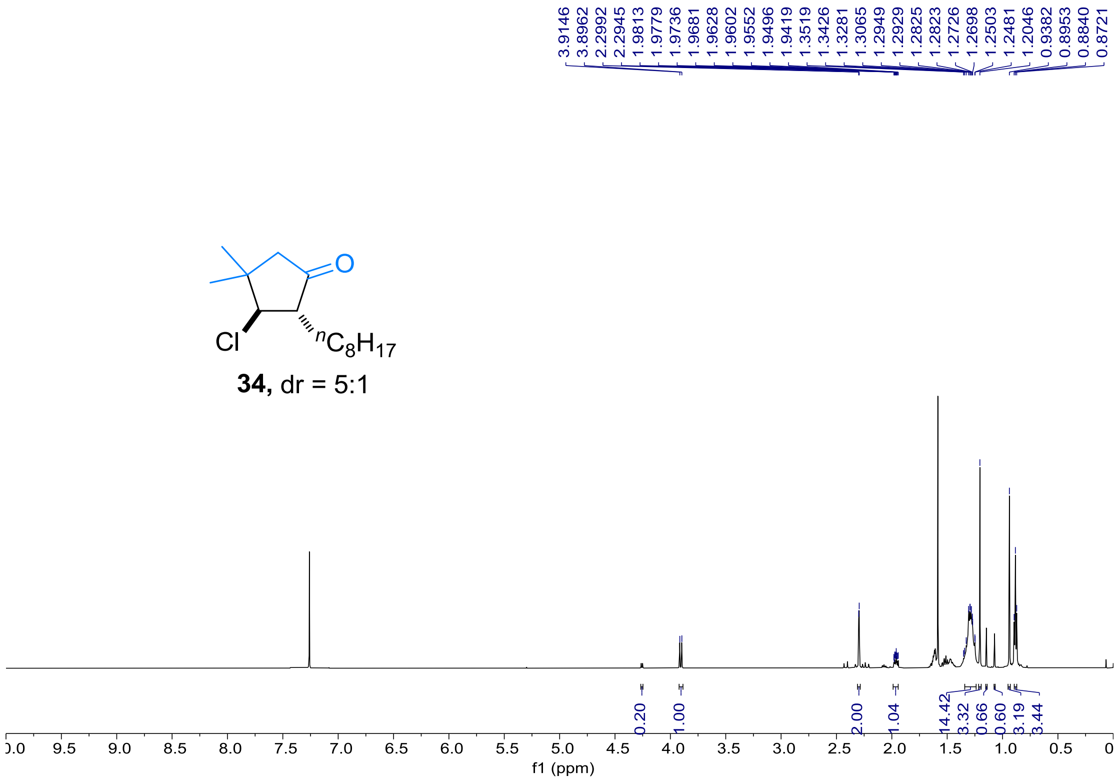

Supplementary Fig. 80. ^{1}H NMR spectrum (600 MHz, $CDCl_3$, 298K) of **34.**

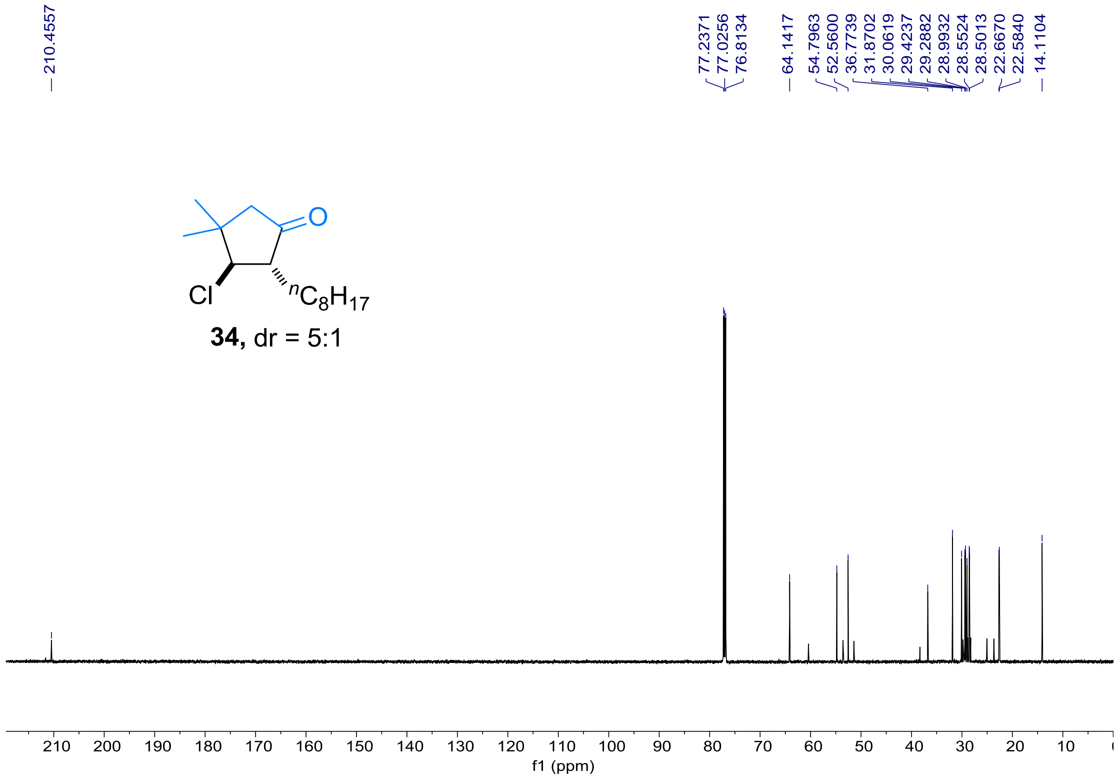

Supplementary Fig. 81. ^{13}C NMR spectrum (151 MHz, $CDCl_3$, 298K) of **34.**

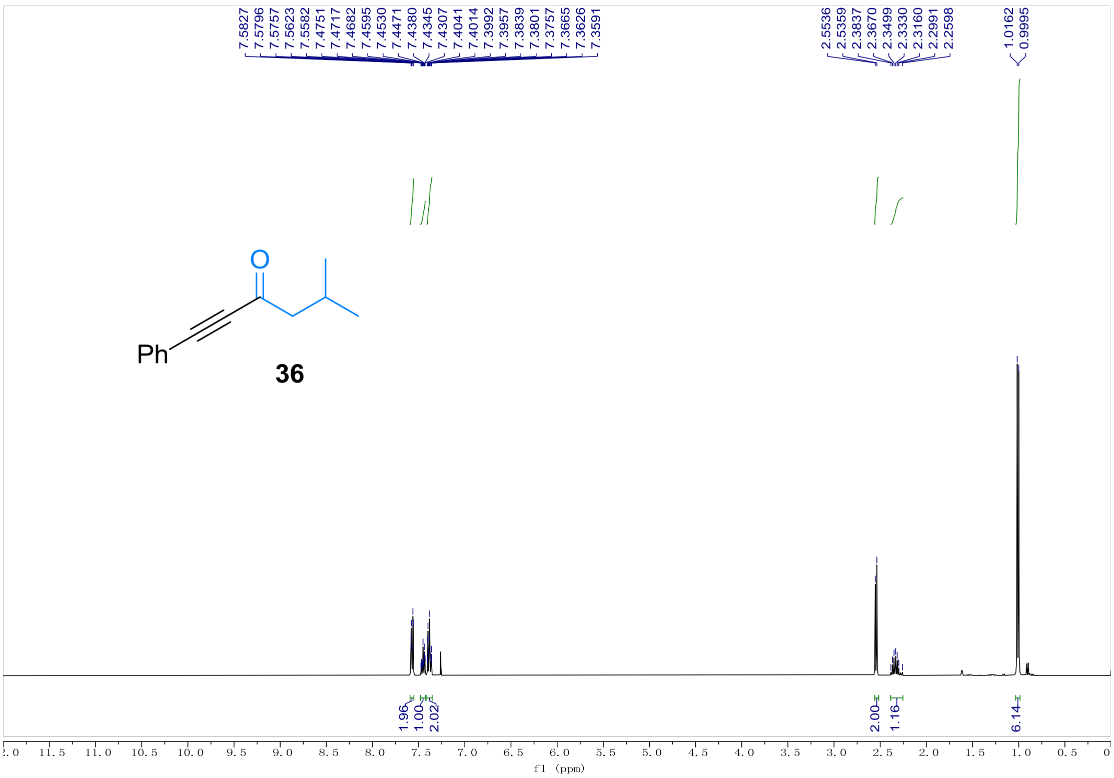

Supplementary Fig. 82. ^{1}H NMR spectrum (400 MHz, $CDCl_3$, 298K) of **36.**

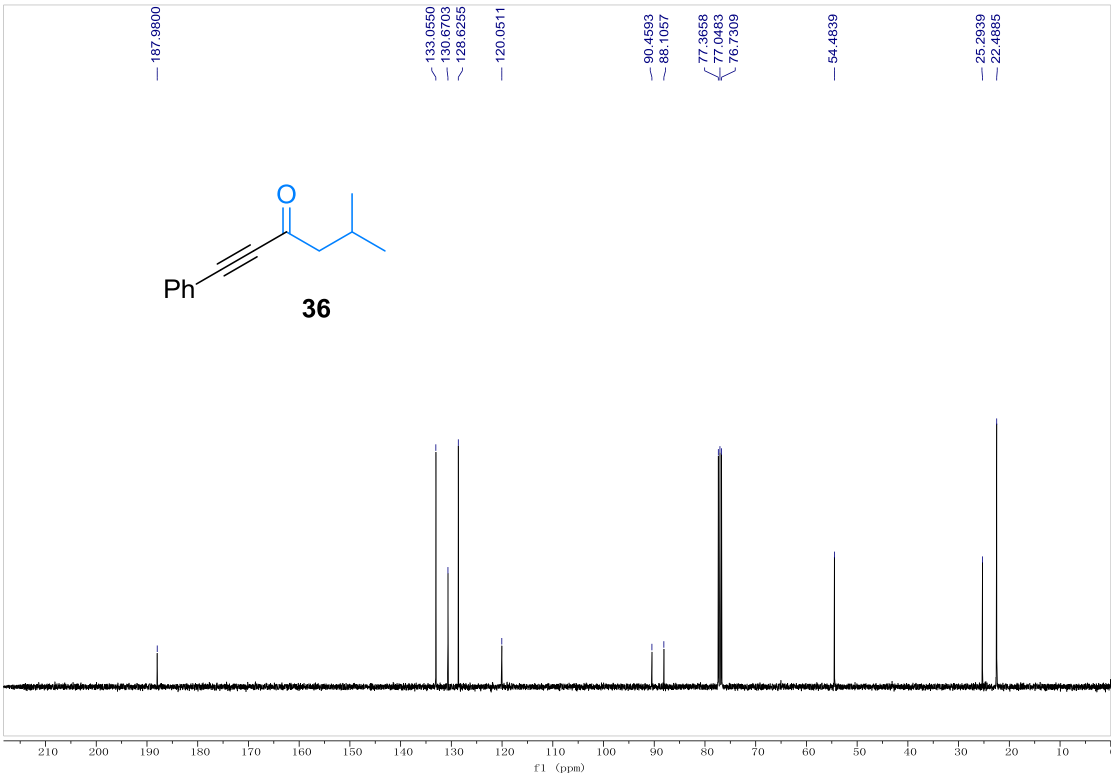

Supplementary Fig. 83. ^{13}C NMR spectrum (101 MHz, $CDCl_3$, 298K) of **36.**

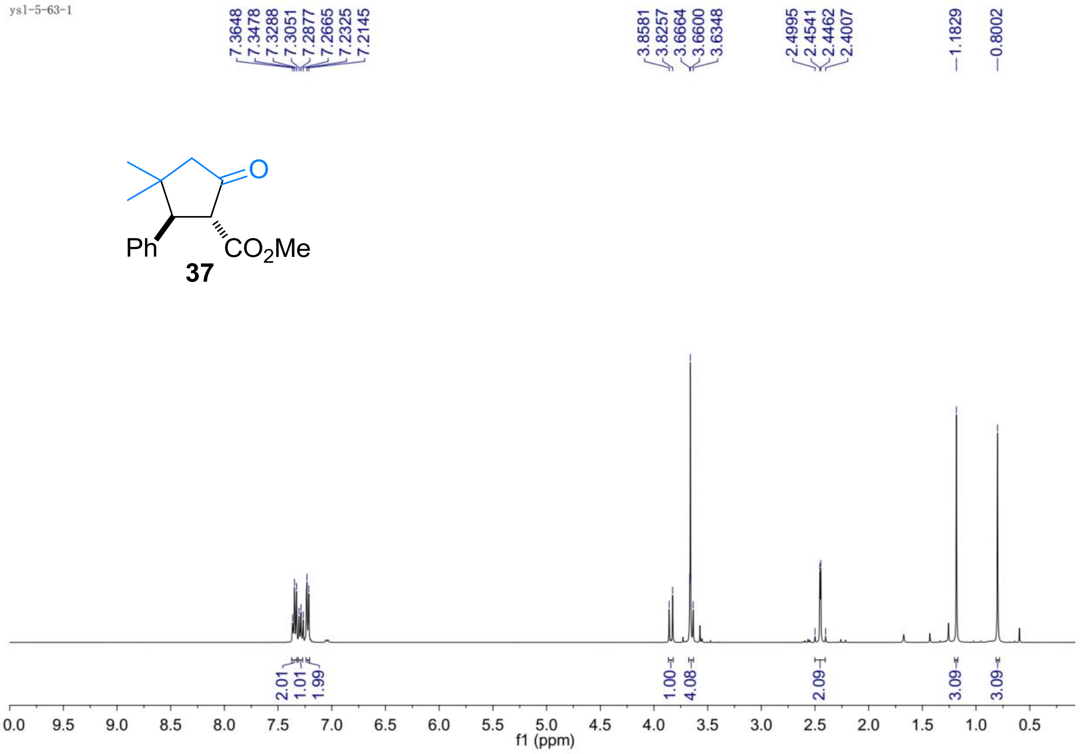

Supplementary Fig. 84. ^{1}H NMR spectrum (400 MHz, $CDCl_3$, 298K) of **37.**

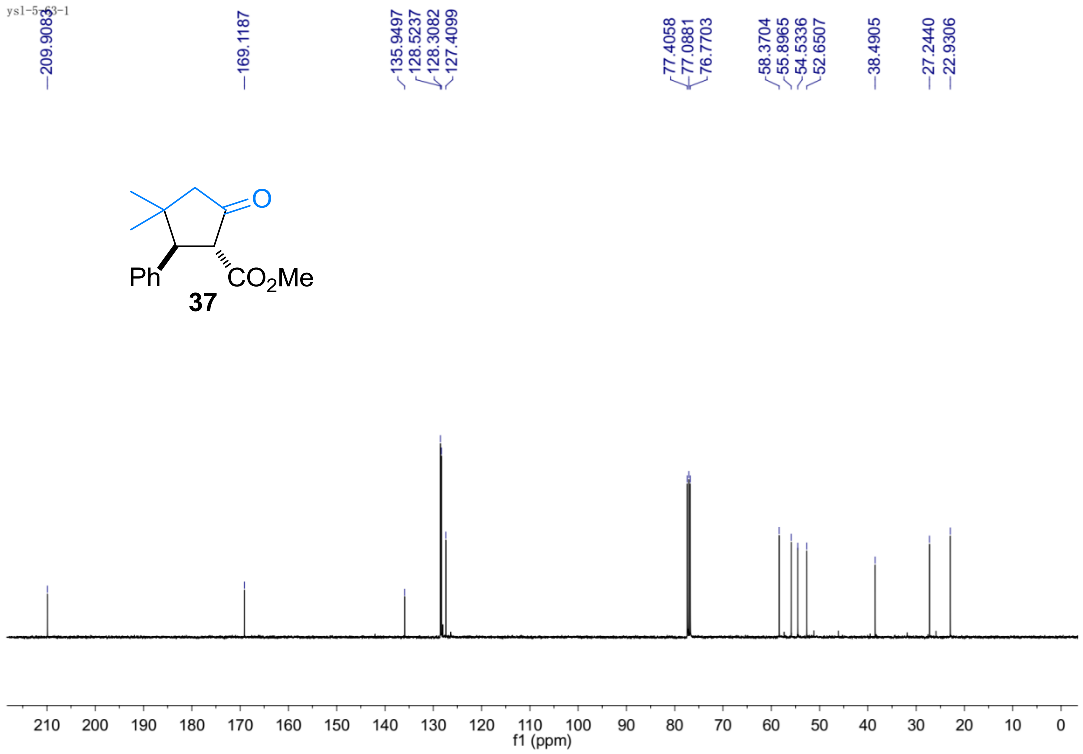

Supplementary Fig. 85. ^{13}C NMR spectrum (101 MHz, $CDCl_3$, 298K) of **37.**

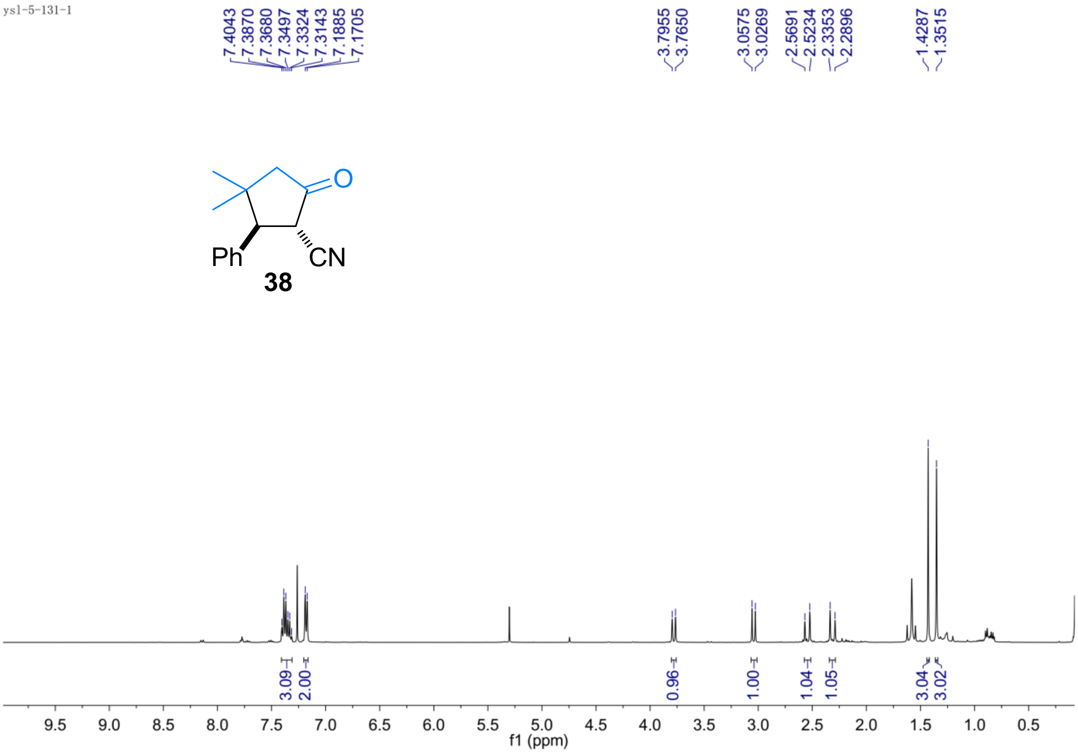

Supplementary Fig. 86. ^{1}H NMR spectrum (400 MHz, $CDCl_3$, 298K) of **38.**

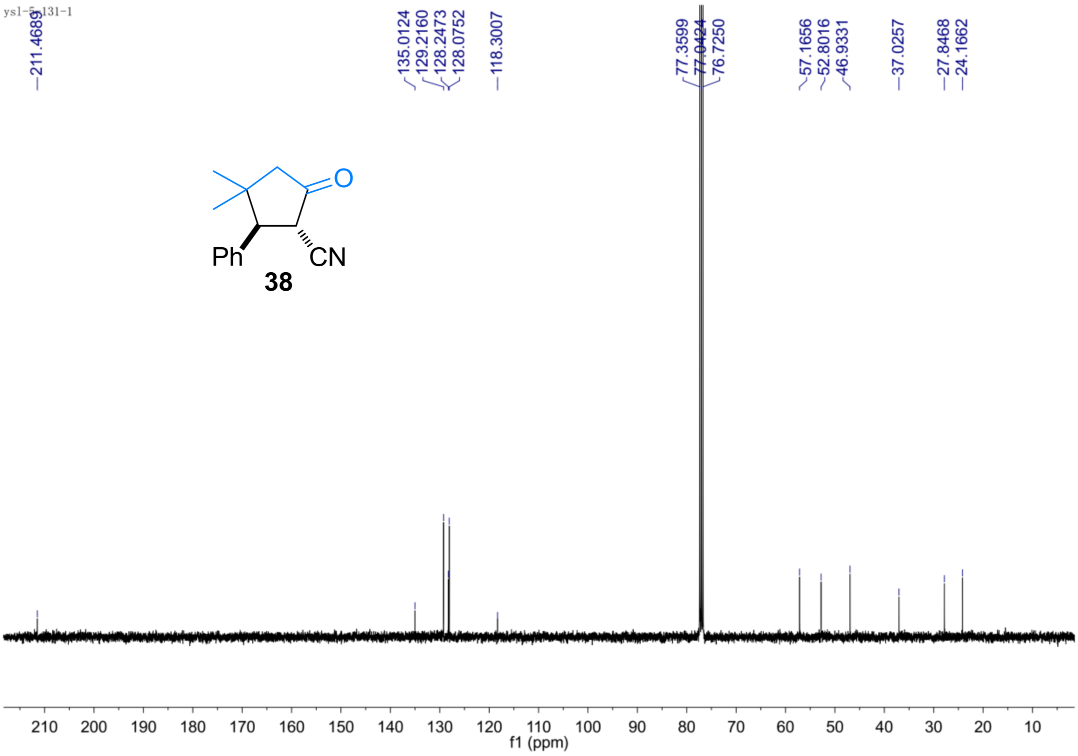

Supplementary Fig. 87. ^{13}C NMR spectrum (101 MHz, $CDCl_3$, 298K) of **38.**

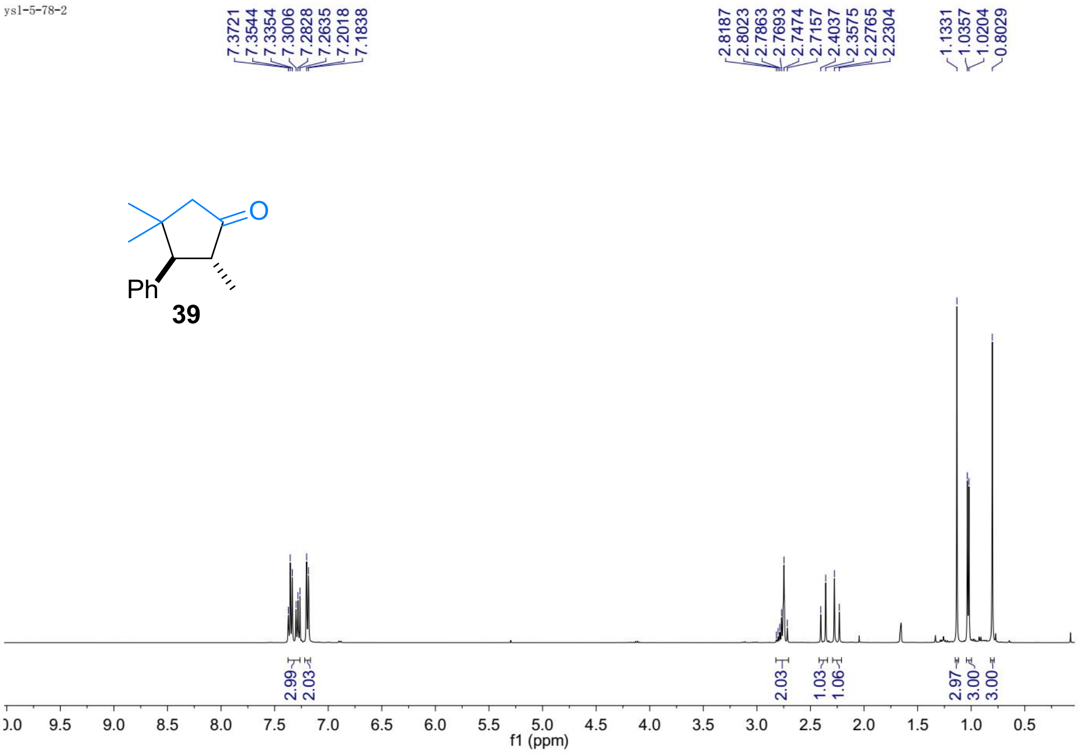

Supplementary Fig. 88. ^{1}H NMR spectrum (400 MHz, $CDCl_3$, 298K) of **39.**

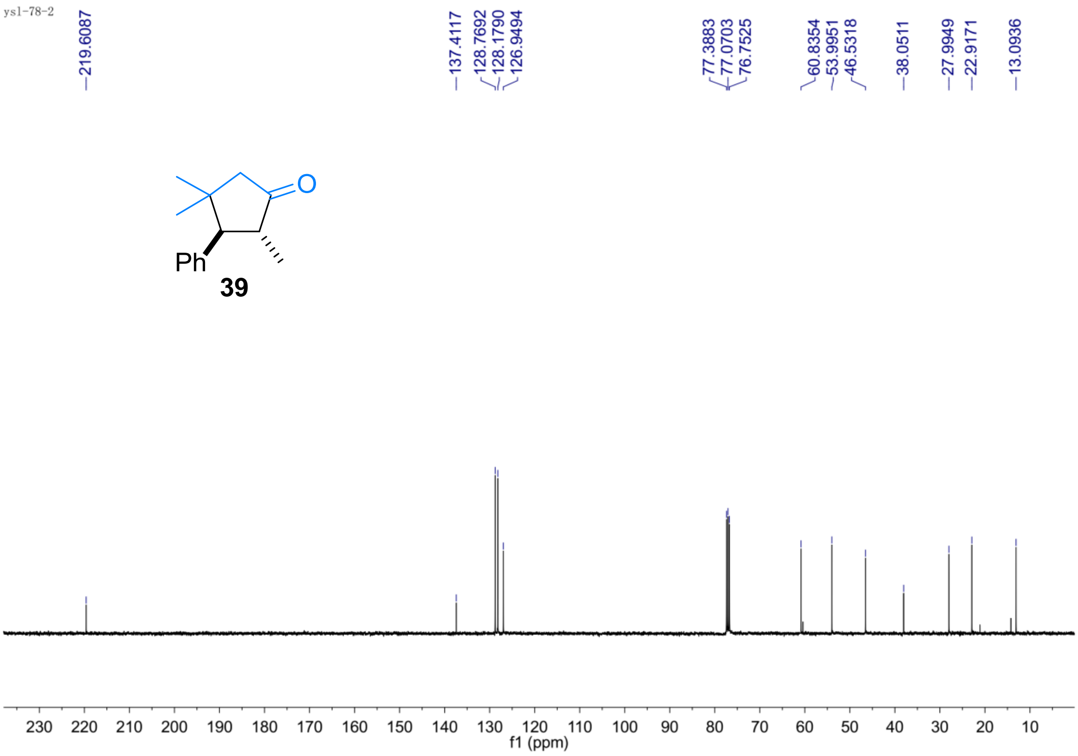

Supplementary Fig. 89. ^{13}C NMR spectrum (101 MHz, $CDCl_3$, 298K) of **39.**

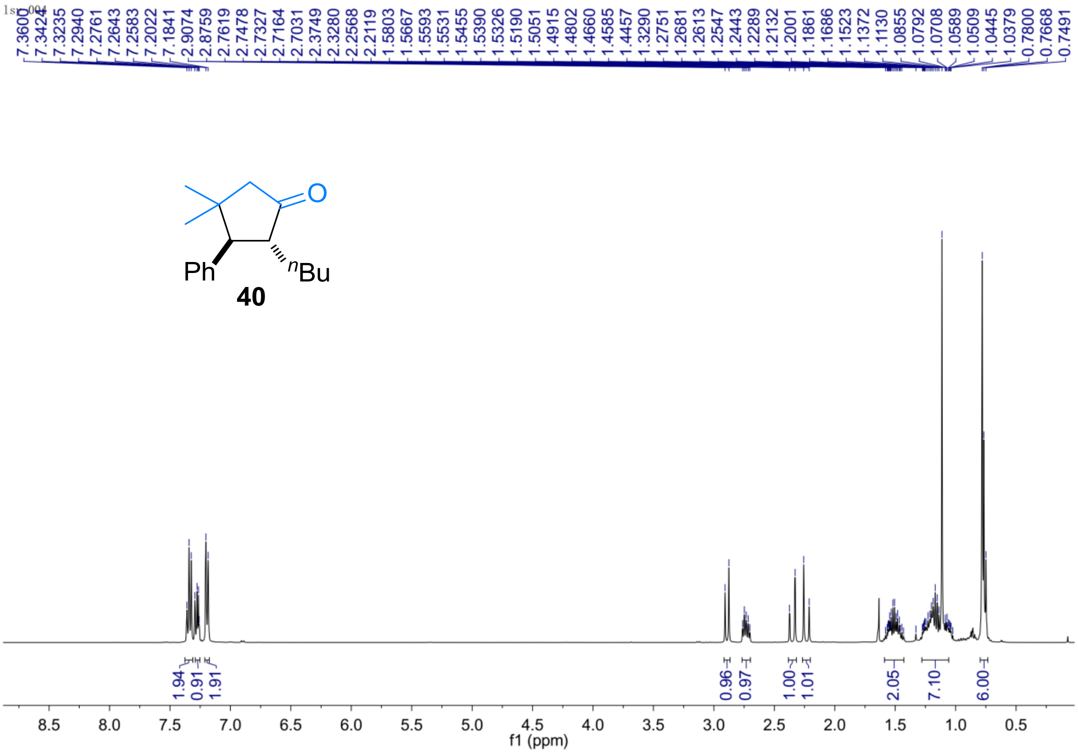

Supplementary Fig. 90. ^{1}H NMR spectrum (400 MHz, $CDCl_3$, 298K) of **40.**

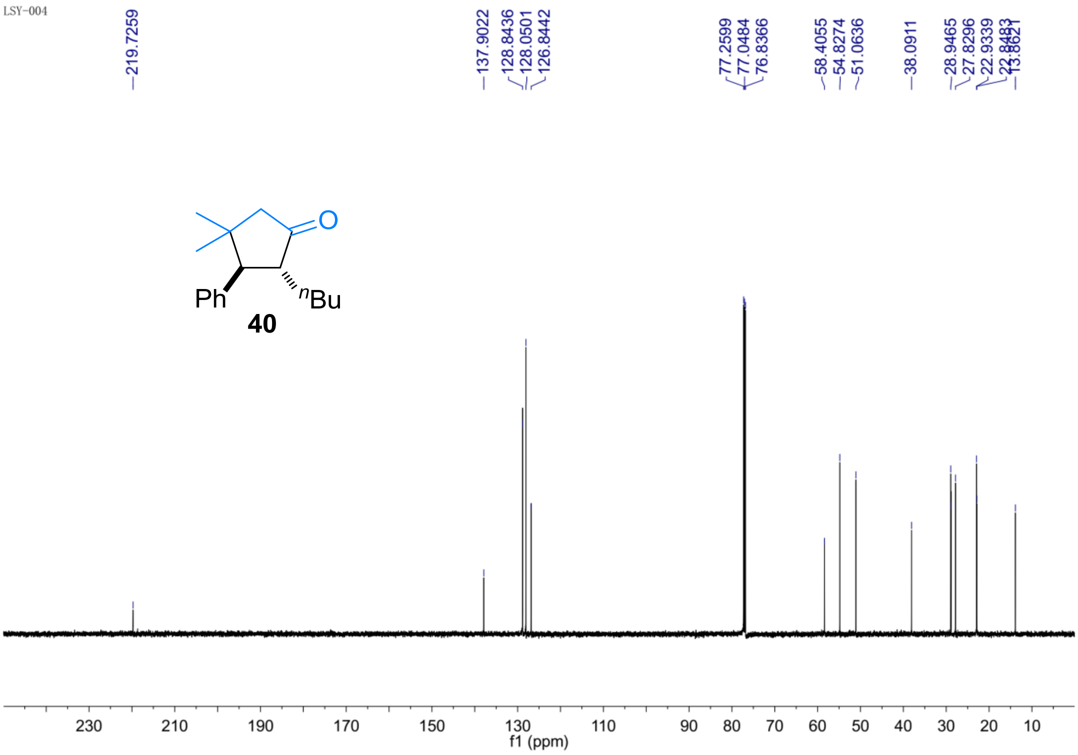

Supplementary Fig. 91. ^{13}C NMR spectrum (151 MHz, $CDCl_3$, 298K) of **40.**

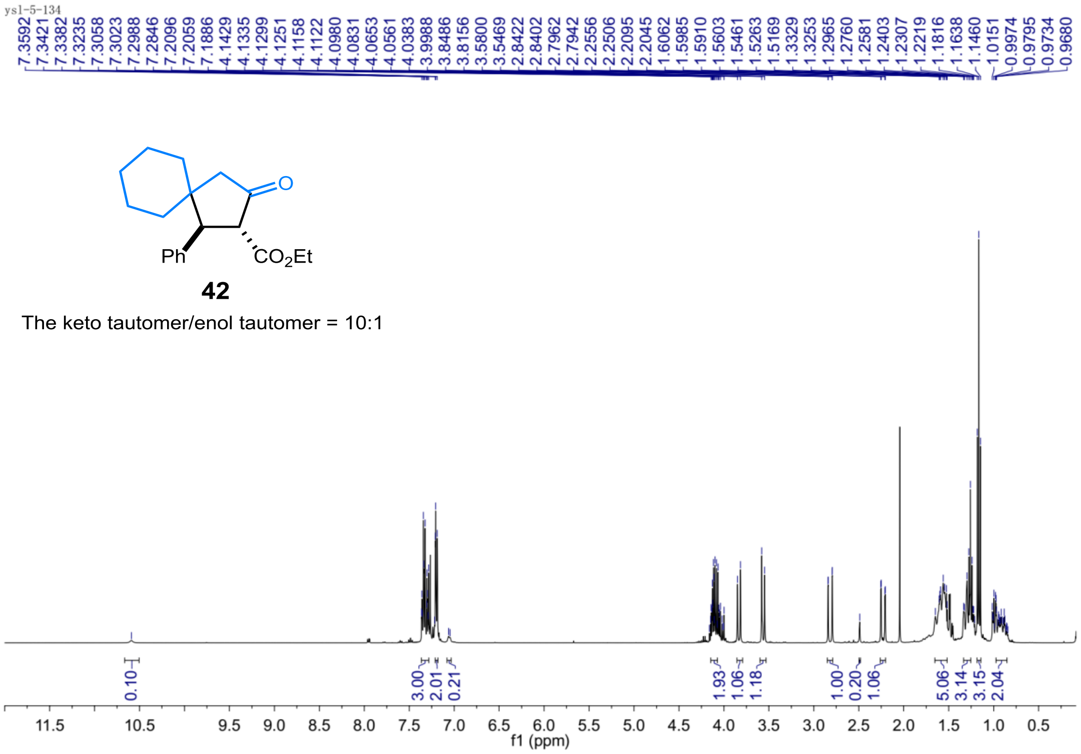

Supplementary Fig. 92. ^{1}H NMR spectrum (400 MHz, $CDCl_3$, 298K) of **42.**

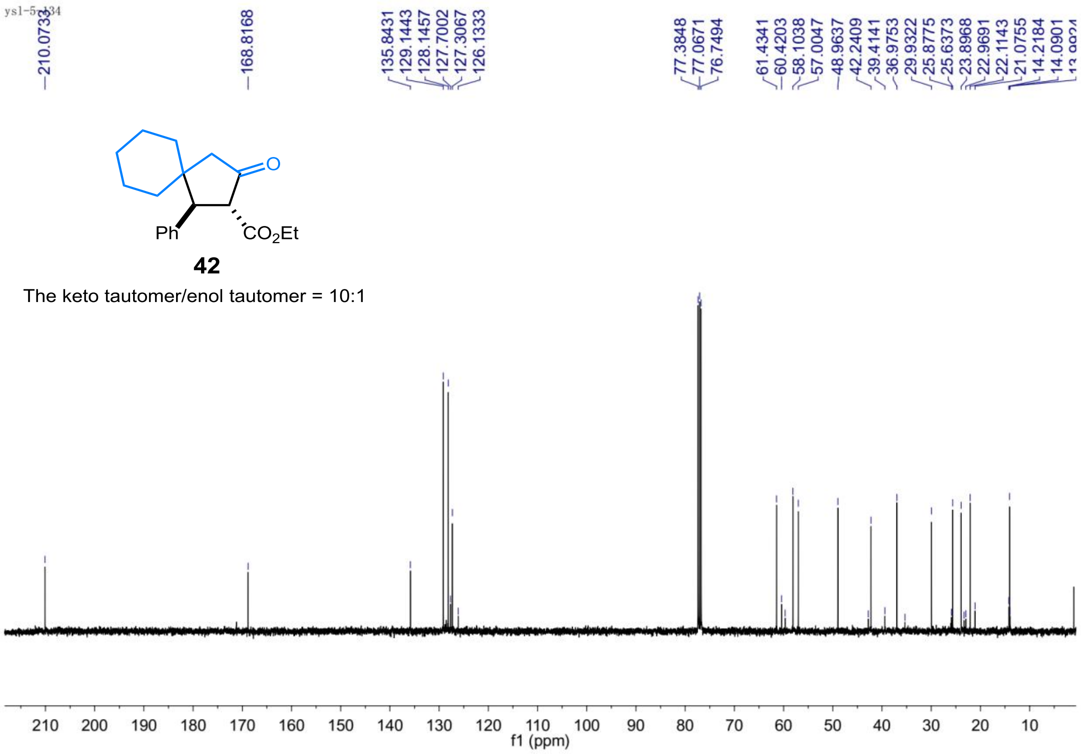

Supplementary Fig. 93. ^{13}C NMR spectrum (101 MHz, $CDCl_3$, 298K) of **42.**

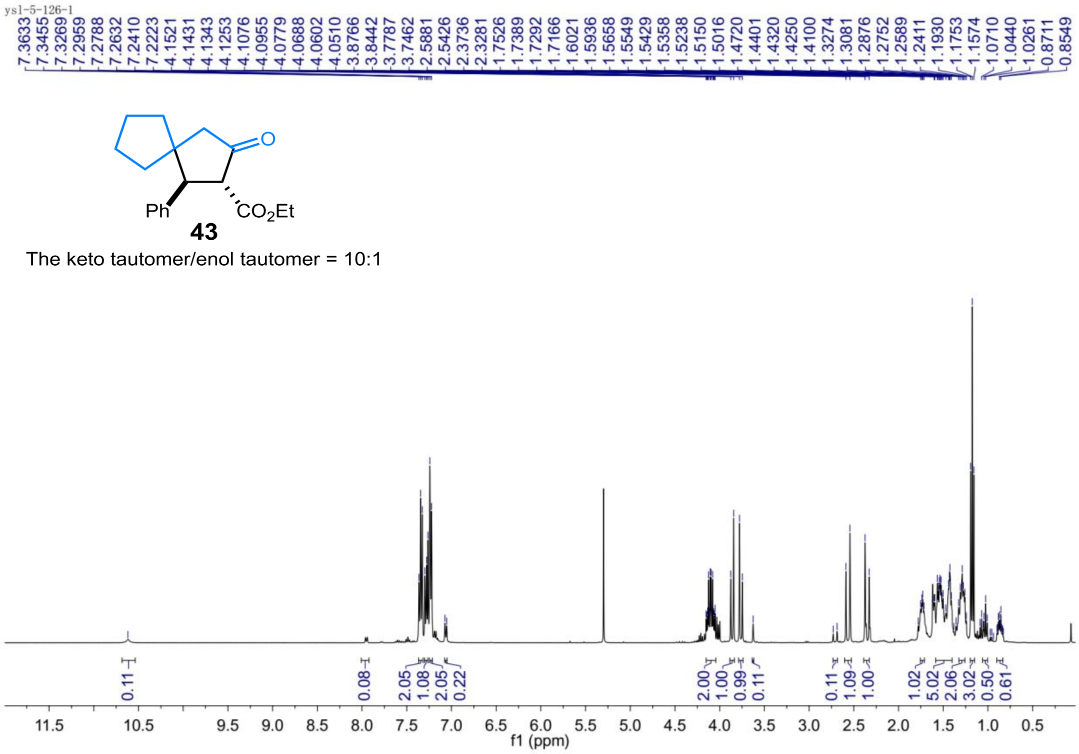

Supplementary Fig. 94. ^{1}H NMR spectrum (400 MHz, $CDCl_3$, 298K) of **43.**

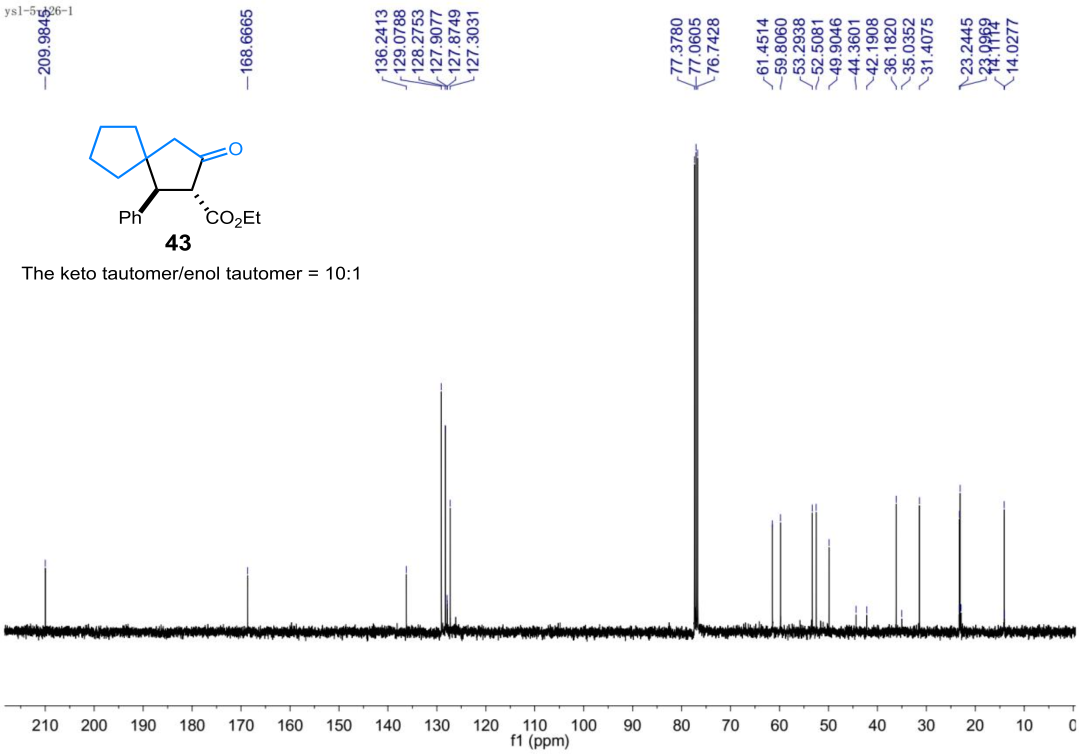

Supplementary Fig. 95. ^{13}C NMR spectrum (101 MHz, $CDCl_3$, 298K) of **43.**

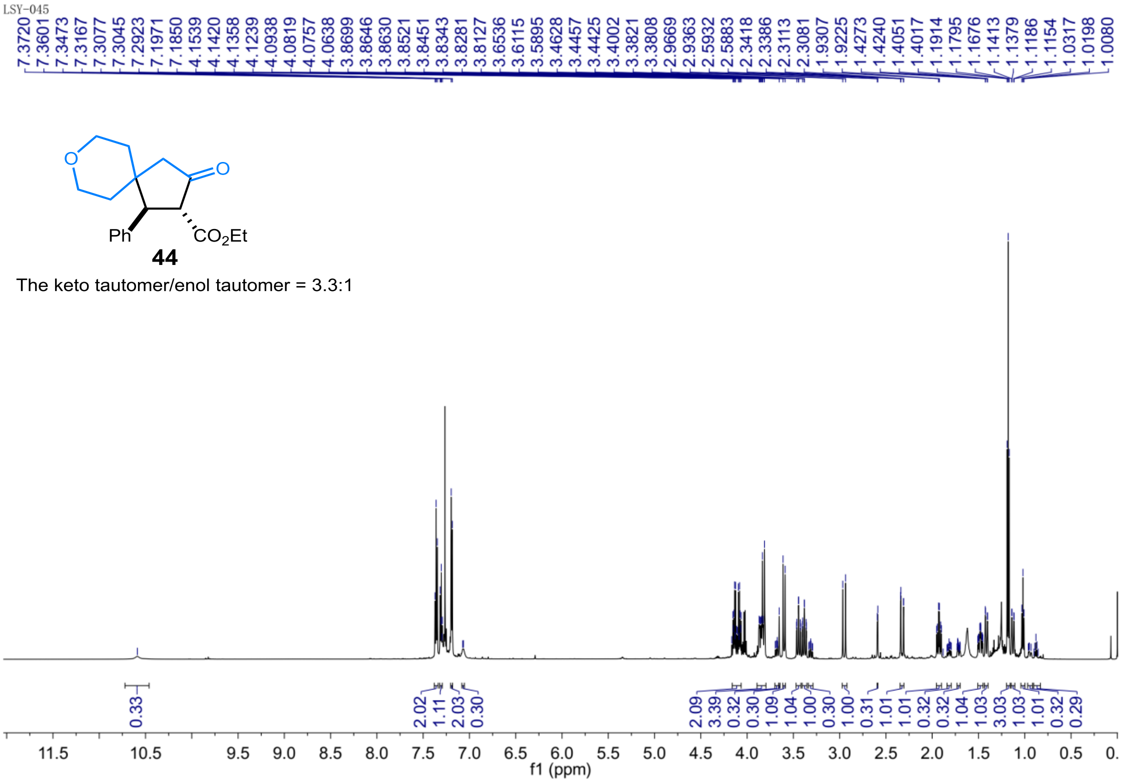

Supplementary Fig. 96. ^{1}H NMR spectrum (600 MHz, $CDCl_3$, 298K) of **44.**

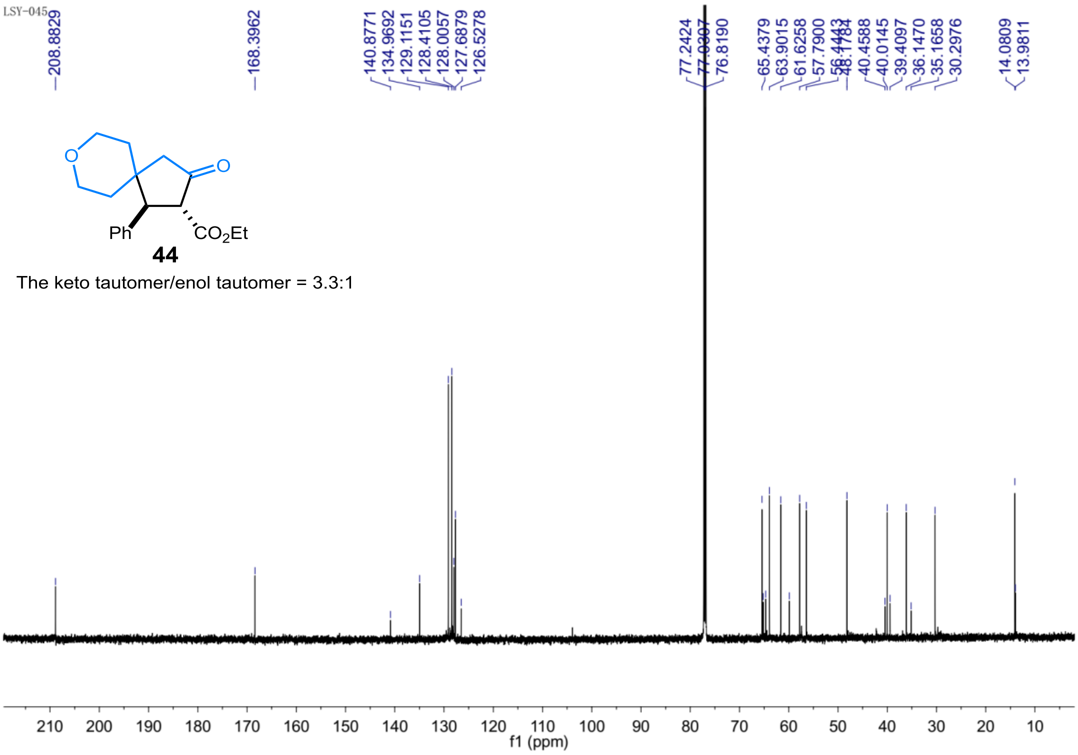

Supplementary Fig. 97. ^{13}C NMR spectrum (151 MHz, $CDCl_3$, 298K) of **44.**

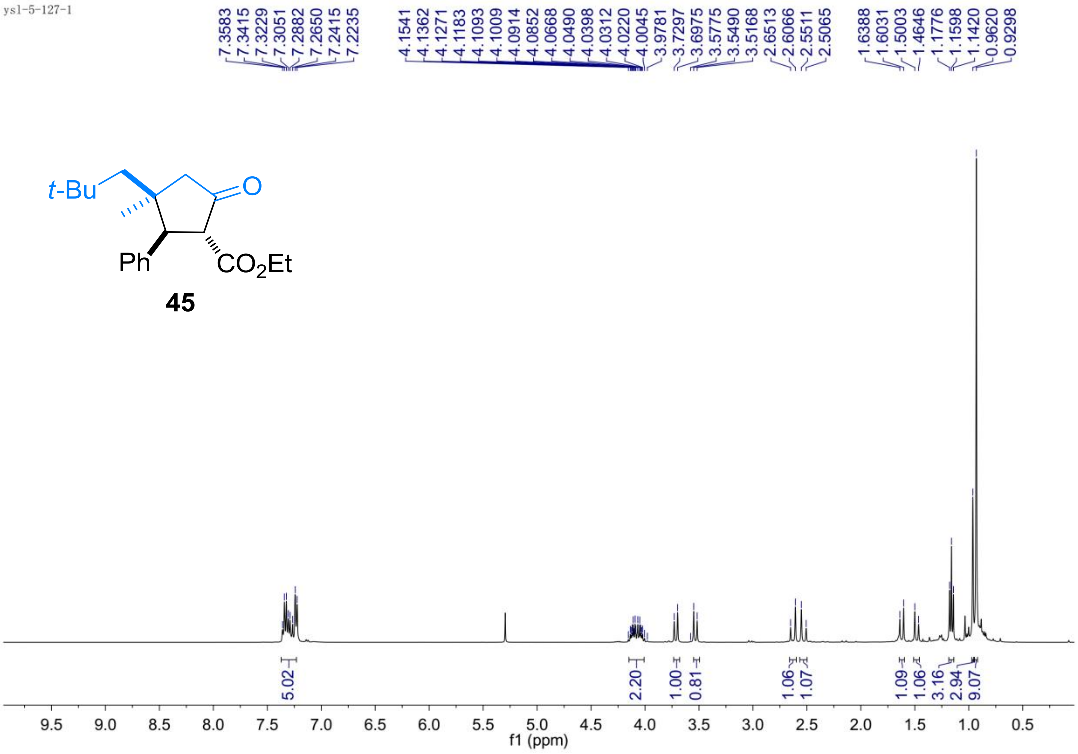

Supplementary Fig. 98. ^{1}H NMR spectrum (400 MHz, $CDCl_3$, 298K) of **45.**

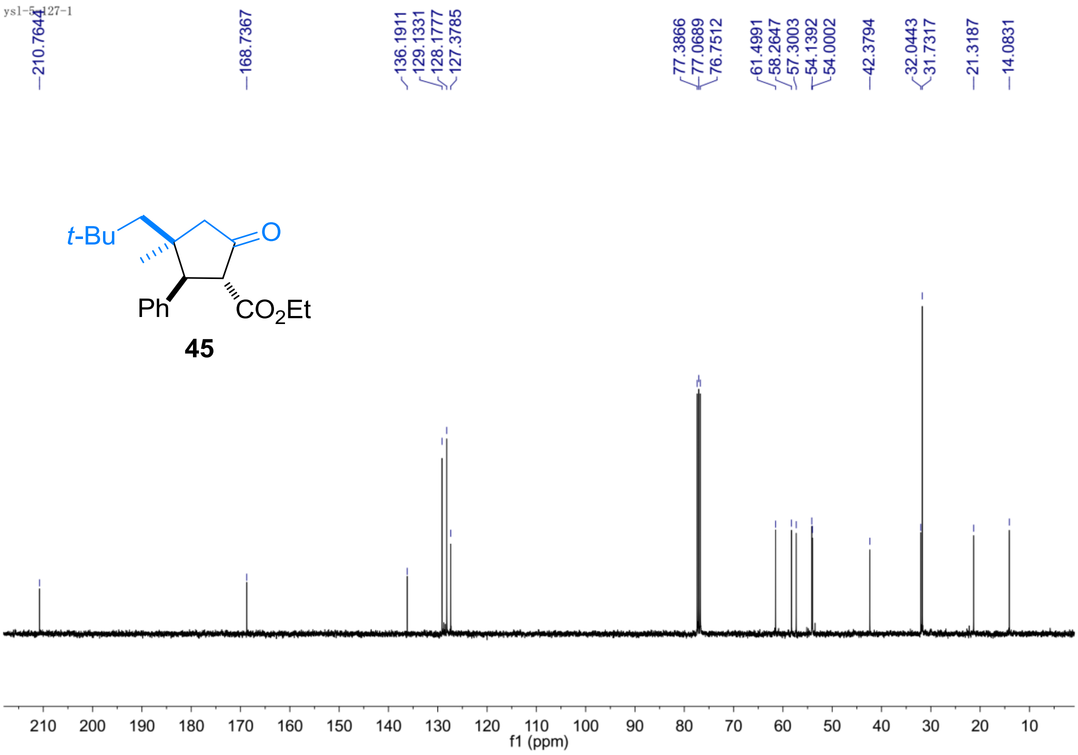

Supplementary Fig. 99. ^{13}C NMR spectrum (101 MHz, $CDCl_3$, 298K) of **45.**

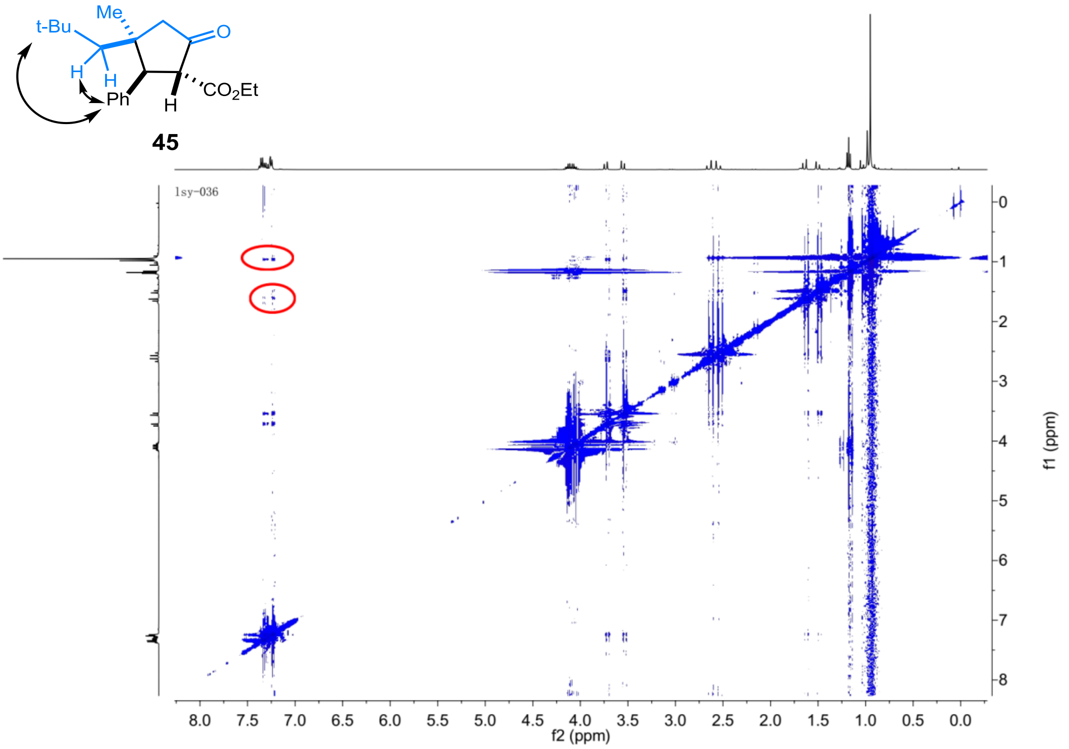

Supplementary Fig. 100. 2D NOESY spectrum ($CDCl_3$, 298K) of **45.**

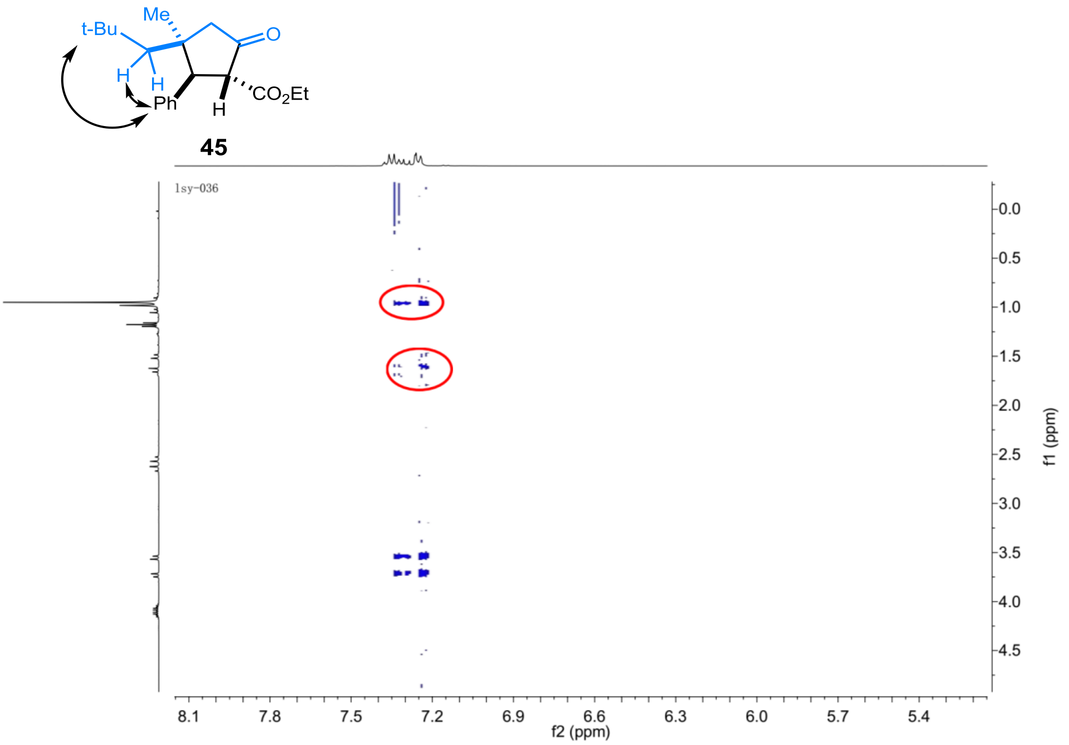

Supplementary Fig. 101. Expansion of 2D NOESY spectrum ($CDCl_3$, 298K) of **45.**

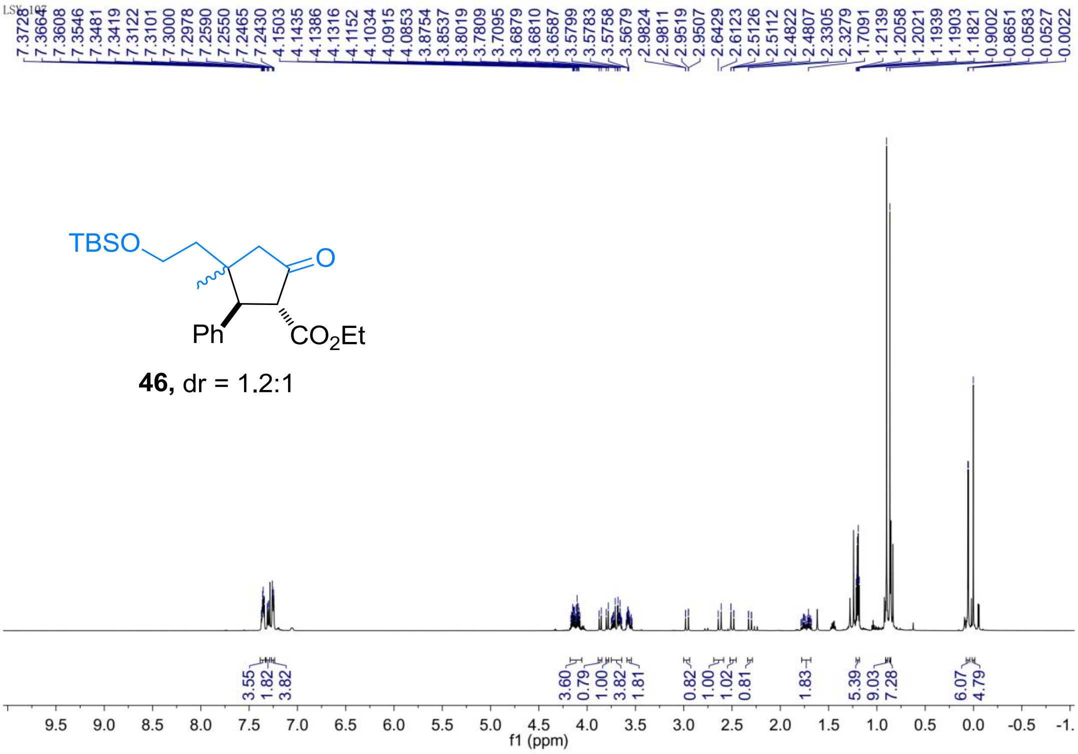

Supplementary Fig. 102. ^{1}H NMR spectrum (600 MHz, $CDCl_3$, 298K) of **46.**

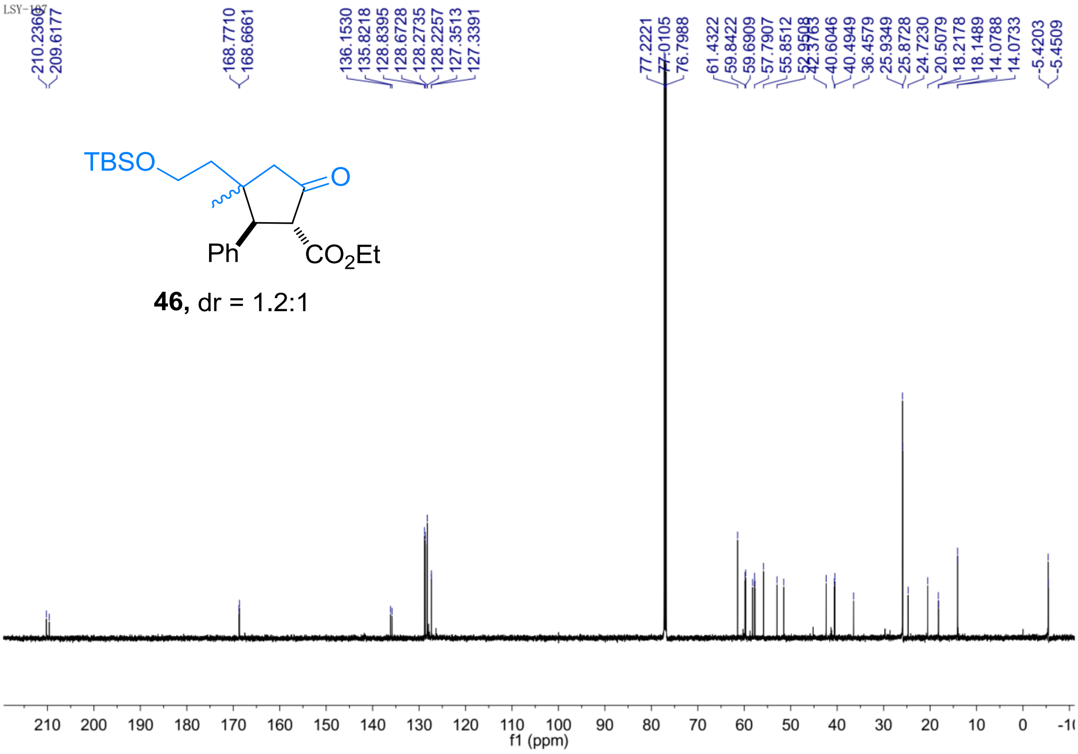

Supplementary Fig. 103. ^{13}C NMR spectrum (151 MHz, $CDCl_3$, 298K) of **46.**

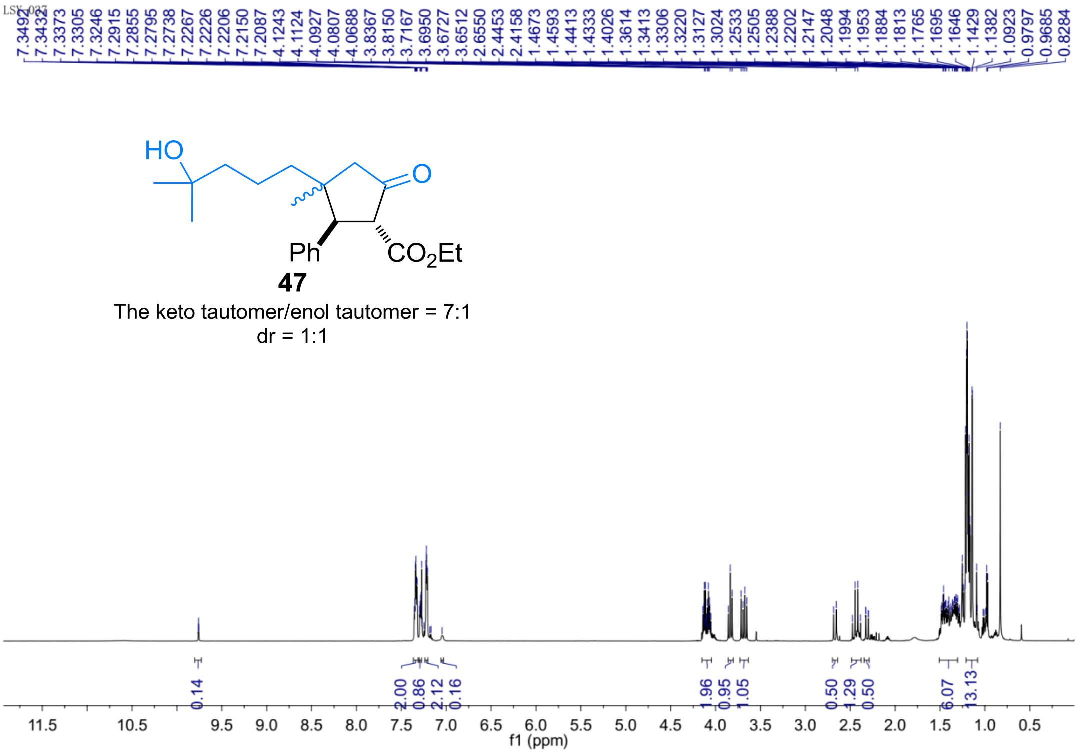

Supplementary Fig. 104. ^{1}H NMR spectrum (600 MHz, $CDCl_3$, 298K) of **47.**

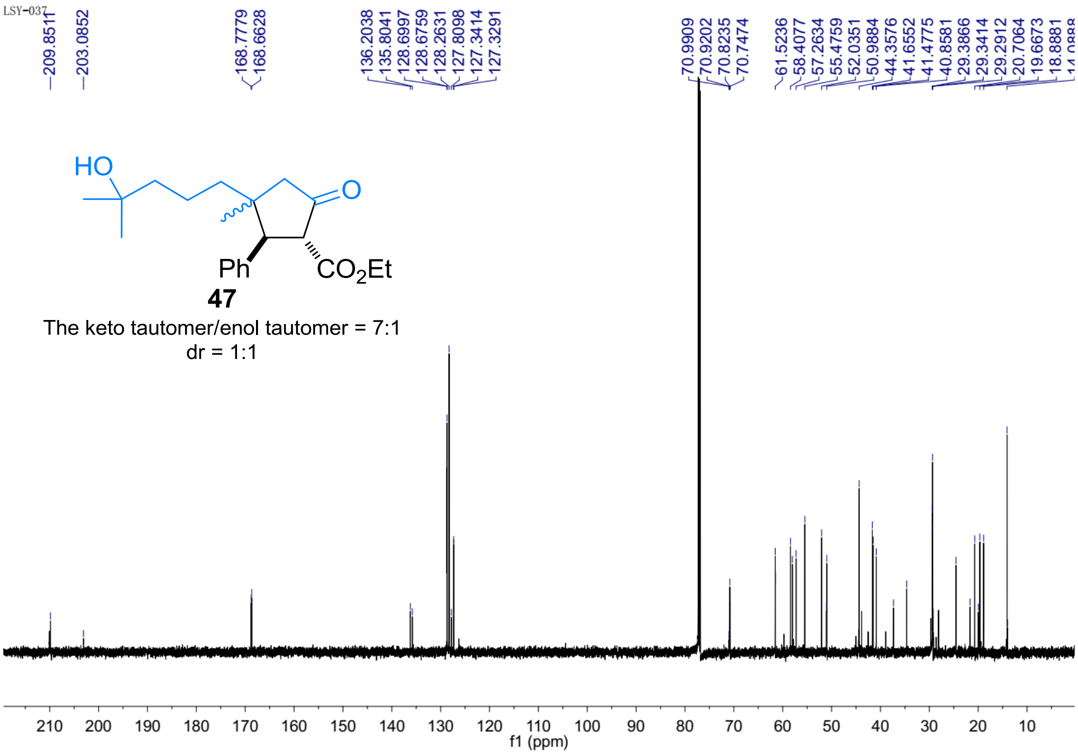

Supplementary Fig. 105. ^{13}C NMR spectrum (151 MHz, $CDCl_3$, 298K) of **47.**

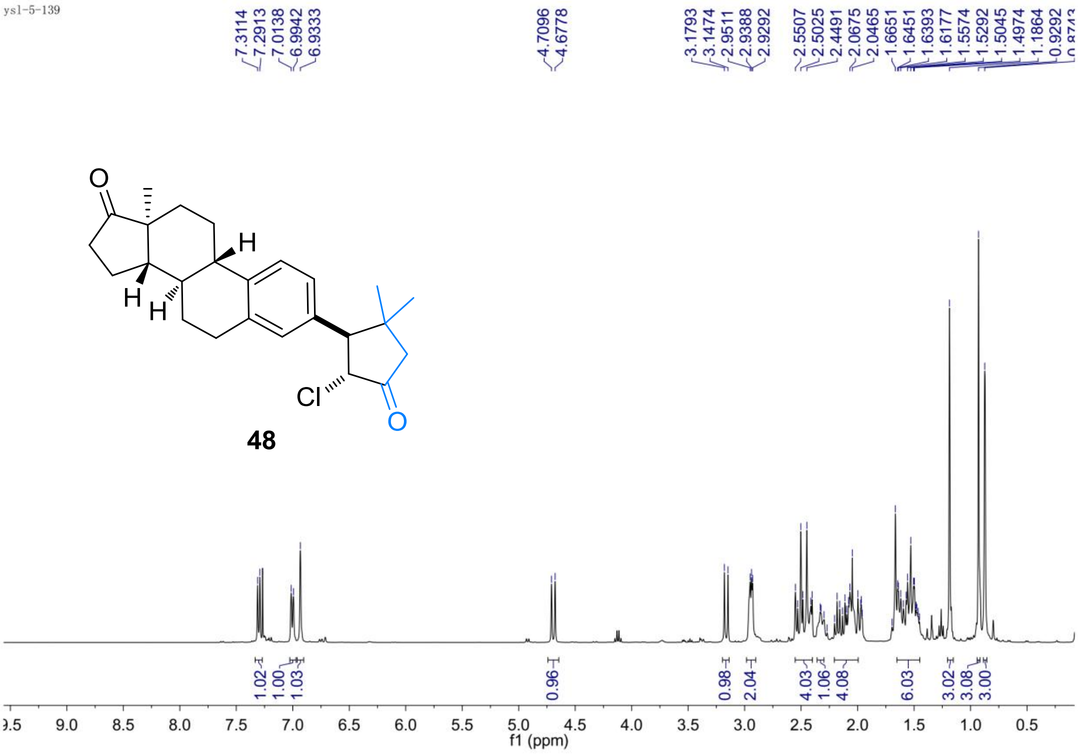

Supplementary Fig. 106. ^{1}H NMR spectrum (400 MHz, $CDCl_3$, 298K) of **48.**

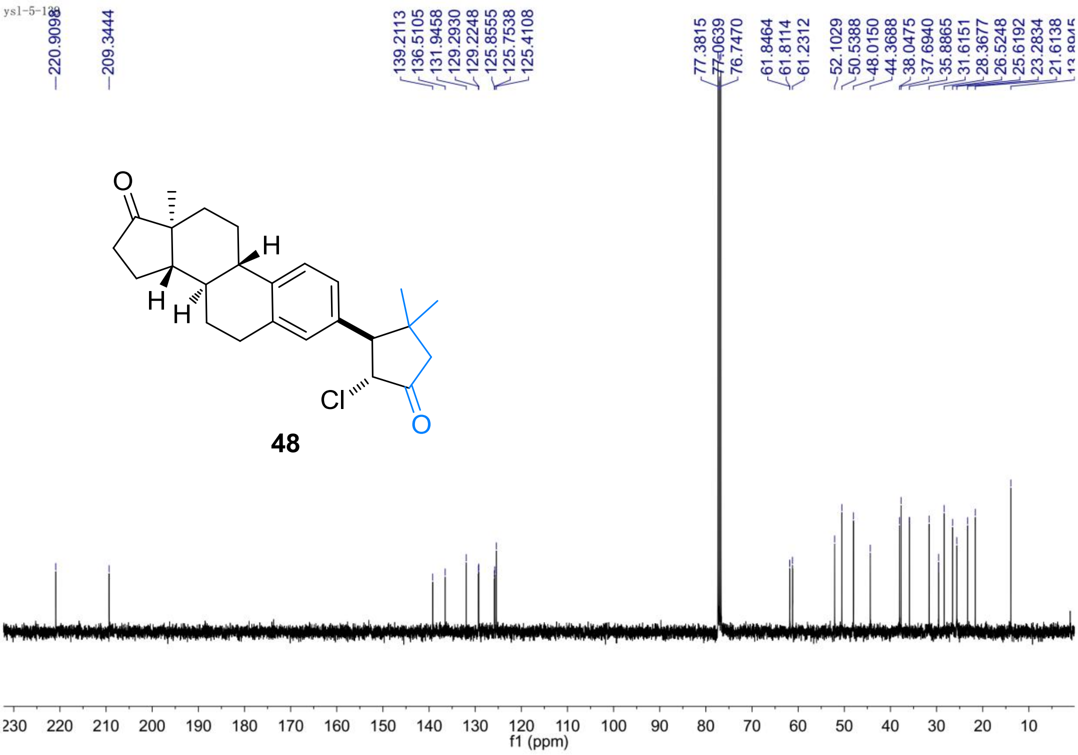

Supplementary Fig. 107. ^{13}C NMR spectrum (101 MHz, $CDCl_3$, 298K) of **48.**

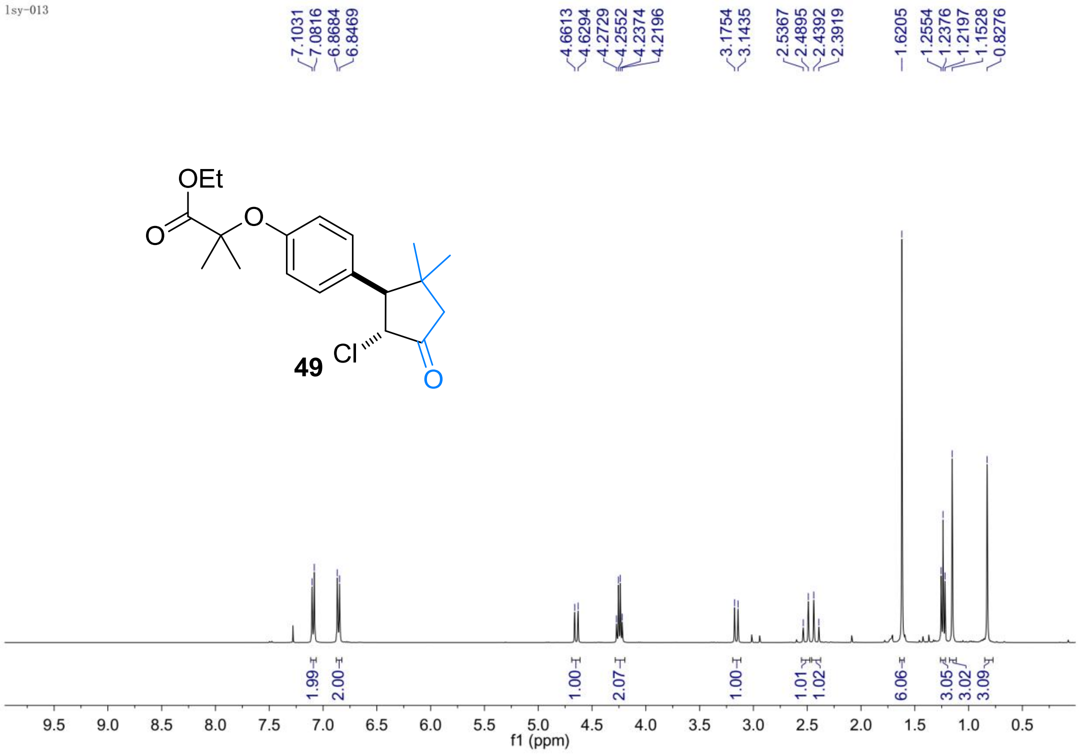

Supplementary Fig. 108. ^{1}H NMR spectrum (400 MHz, $CDCl_3$, 298K) of **49.**

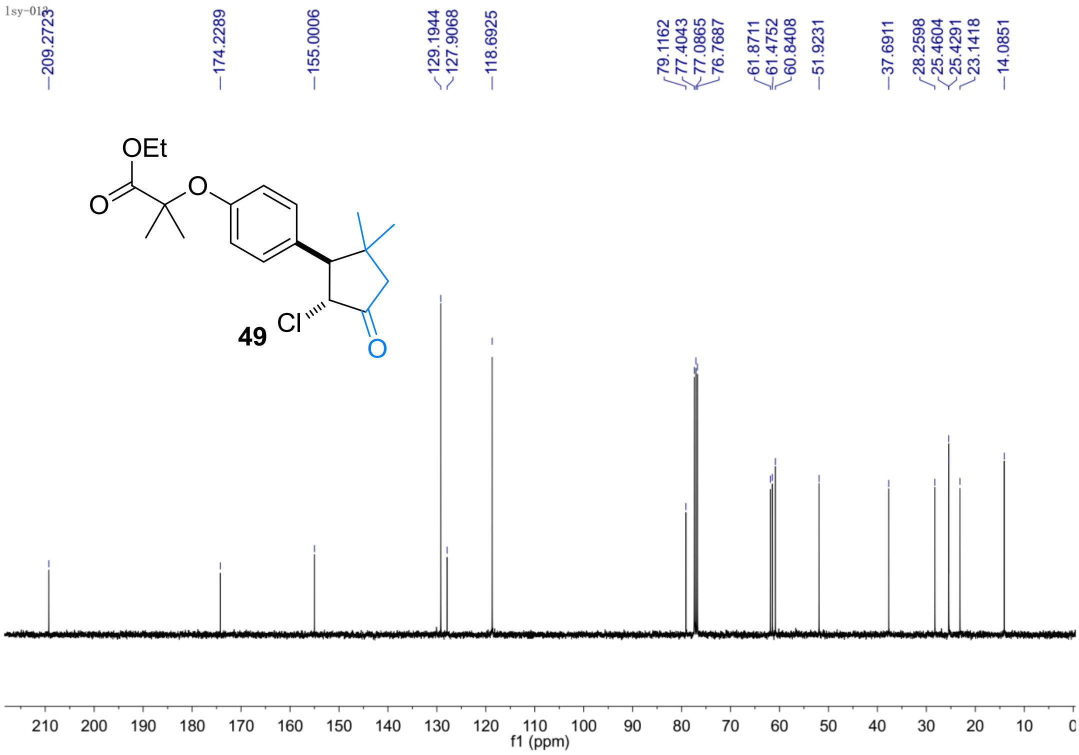

Supplementary Fig.109. ^{13}C NMR spectrum (101 MHz, $CDCl_3$, 298K) of **49.**

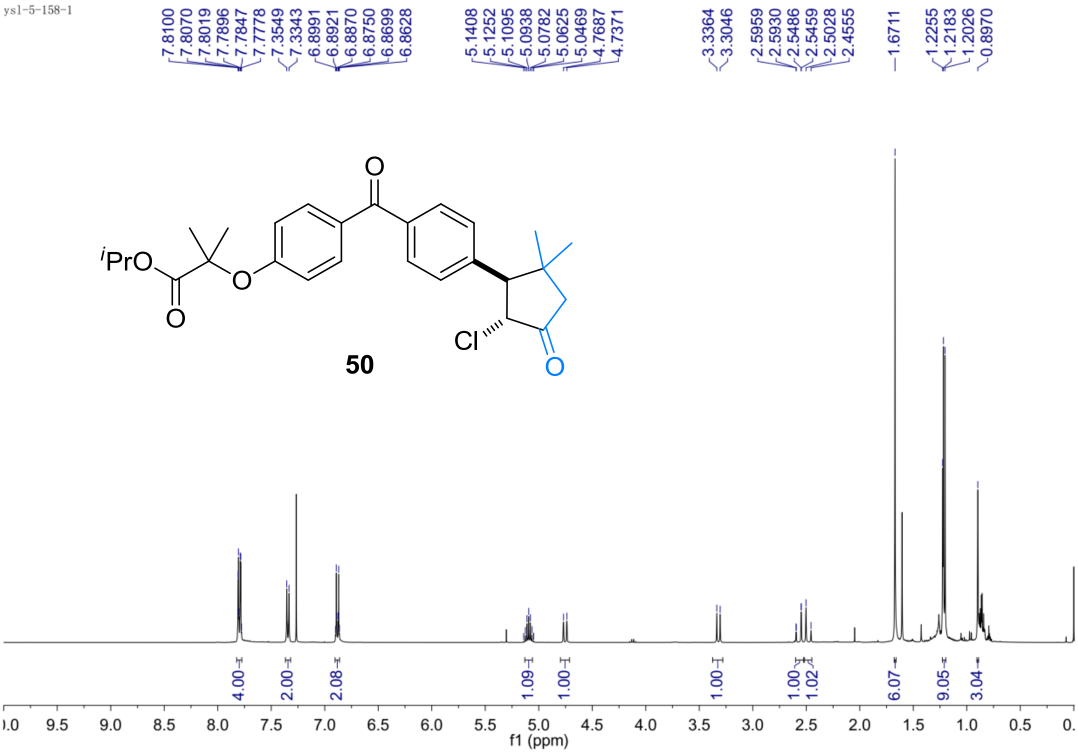

Supplementary Fig. 110. ^{1}H NMR spectrum (400 MHz, $CDCl_3$, 298K) of **50.**

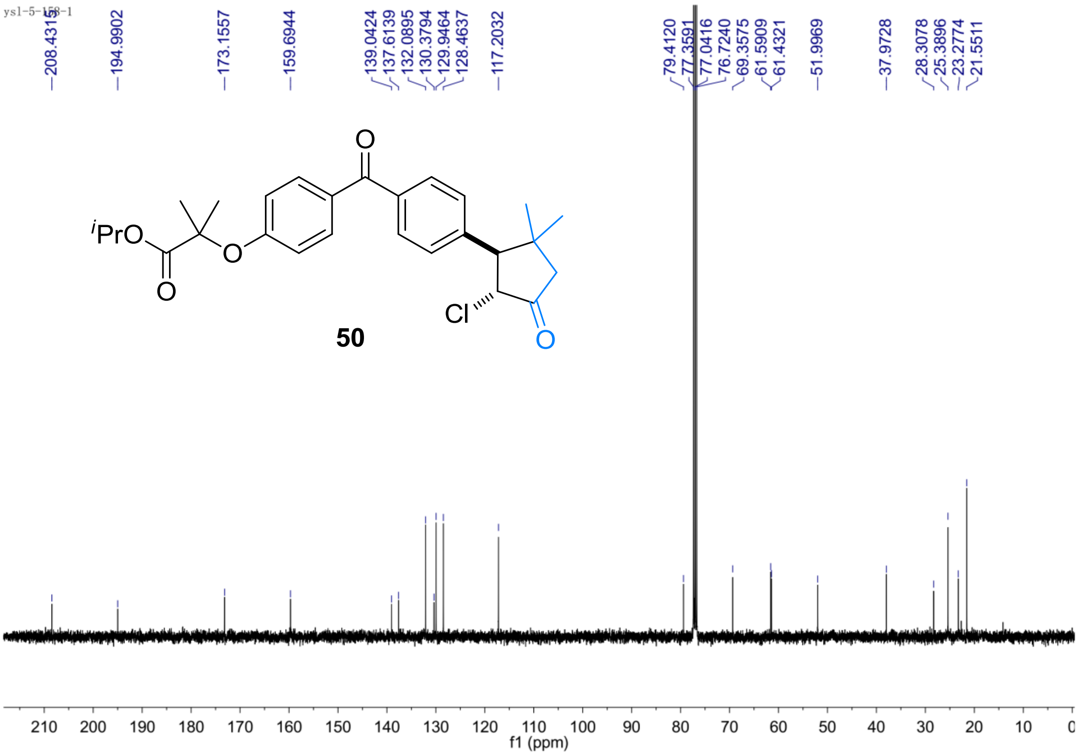

Supplementary Fig. 111. ^{13}C NMR spectrum (101 MHz, $CDCl_3$, 298K) of **50.**

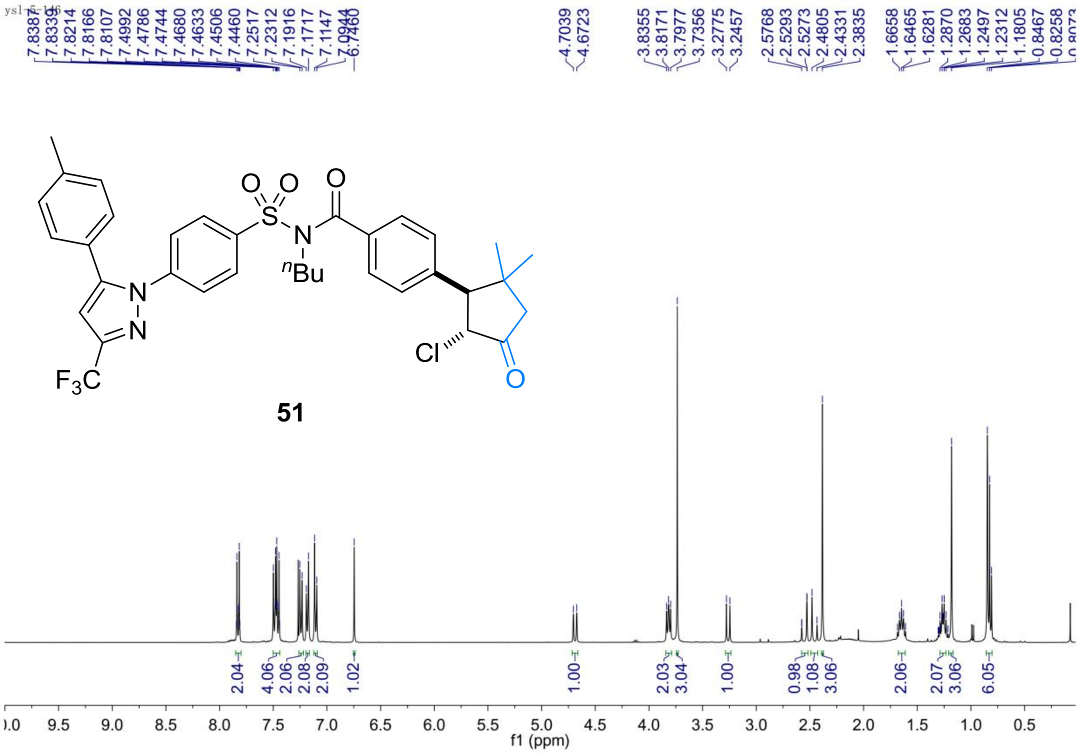

Supplementary Fig. 112. ^{1}H NMR spectrum (400 MHz, $CDCl_3$, 298K) of **51.**

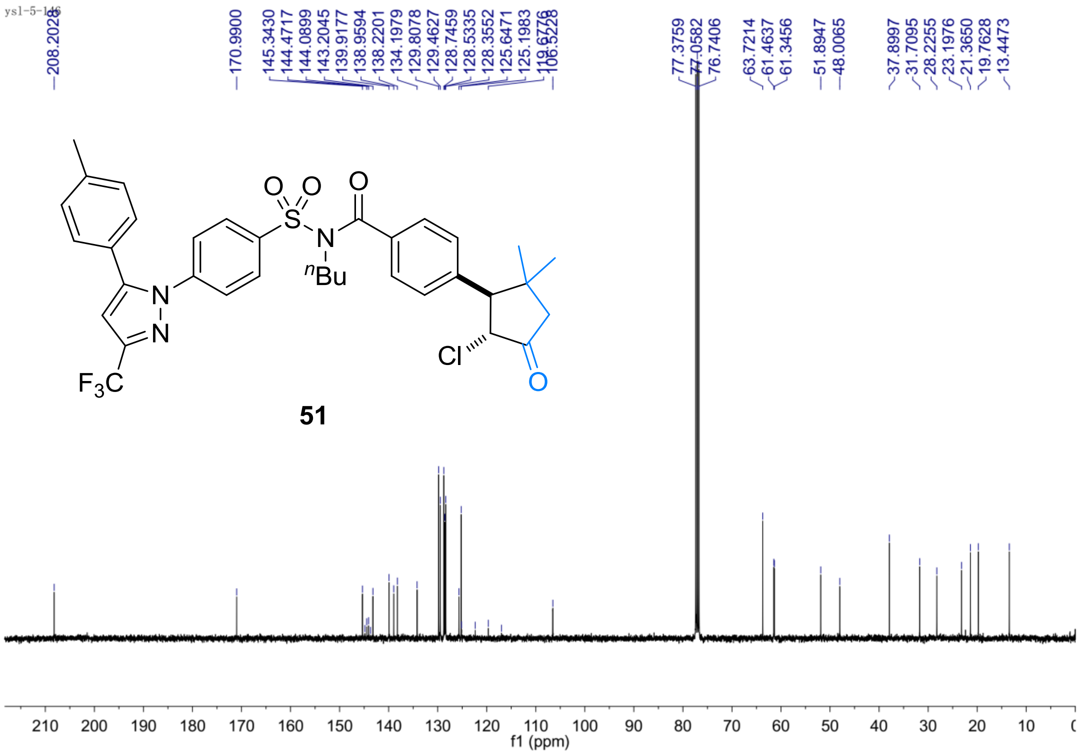

Supplementary Fig. 113. ^{13}C NMR spectrum (101 MHz, $CDCl_3$, 298K) of **51.**

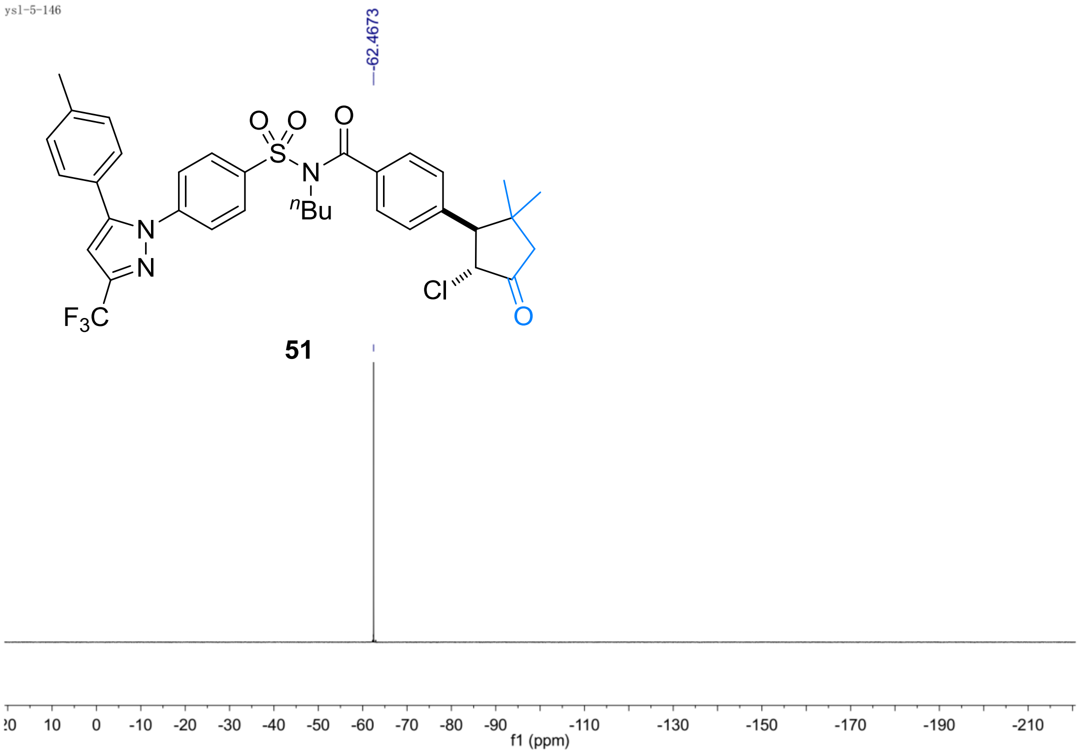

Supplementary Fig. 114. ^{19}F NMR spectrum (377 MHz, $CDCl_3$, 298K) of **51.**

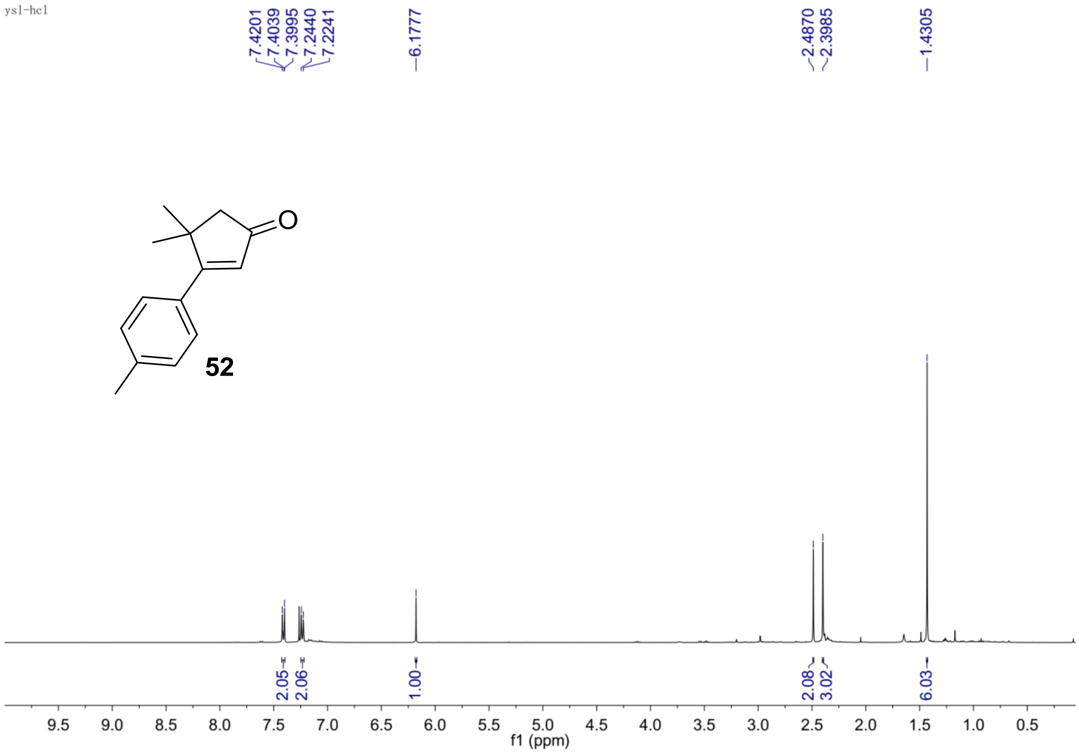

Supplementary Fig. 115. ^{1}H NMR spectrum (600 MHz, $CDCl_3$, 298K) of **52.**

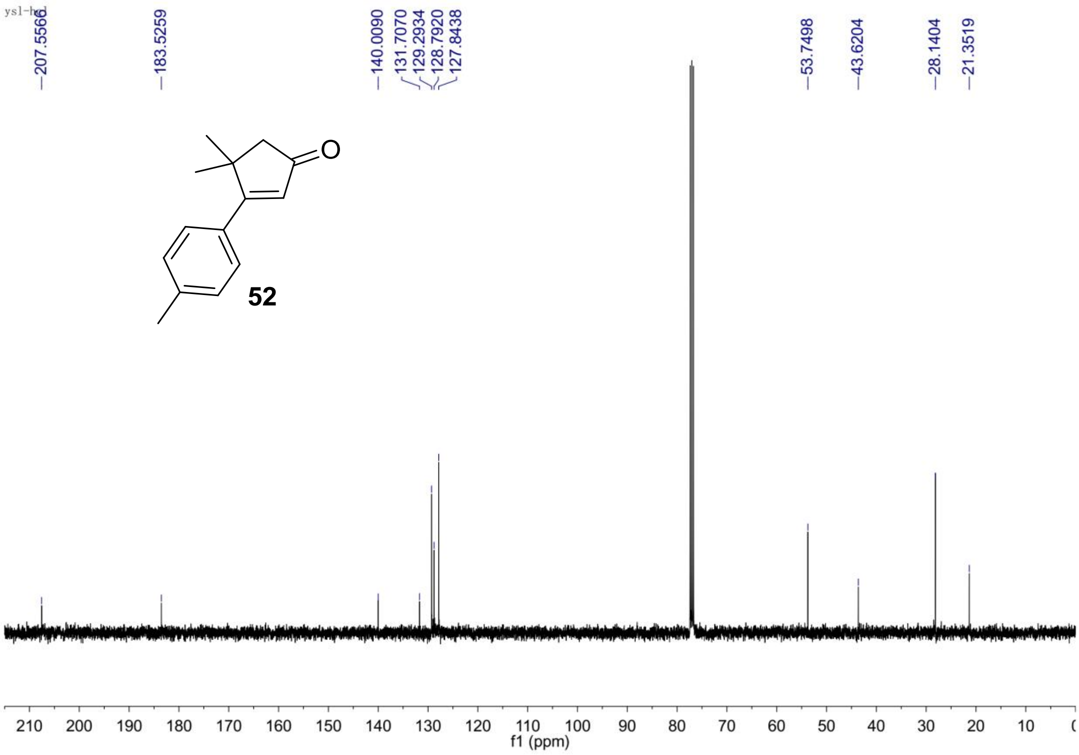

Supplementary Fig. 116. ^{13}C NMR spectrum (151 MHz, $CDCl_3$, 298K) of **52.**

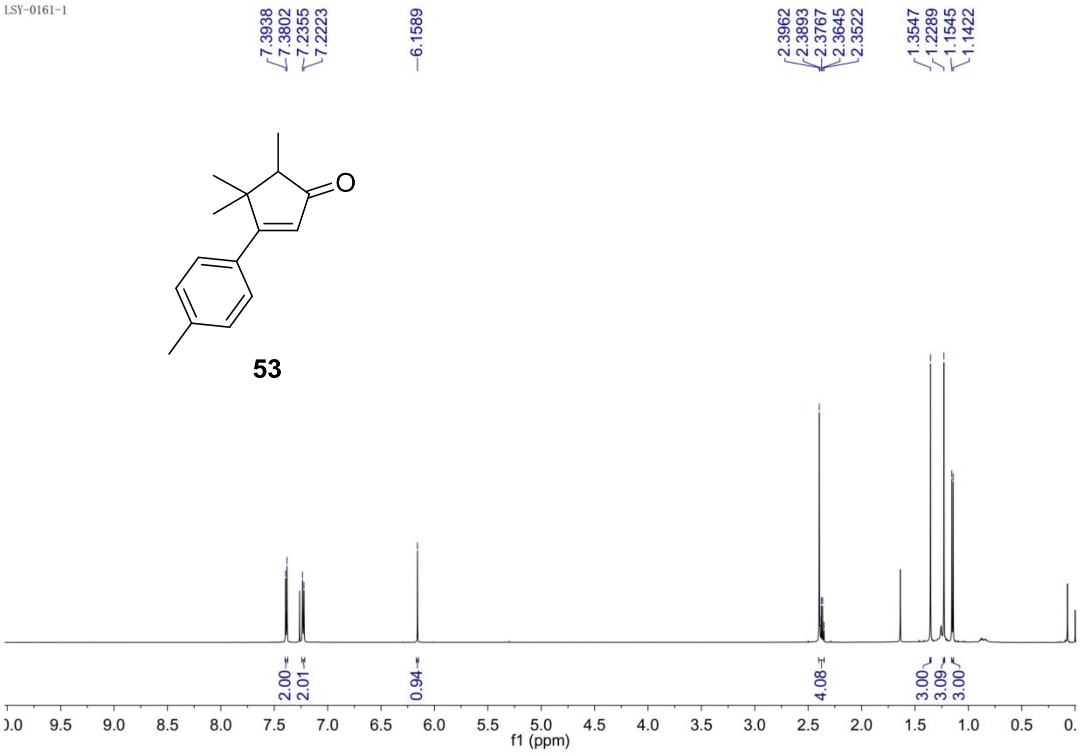

Supplementary Fig. 117. ^{1}H NMR spectrum (600 MHz, $CDCl_3$, 298K) of **53.**

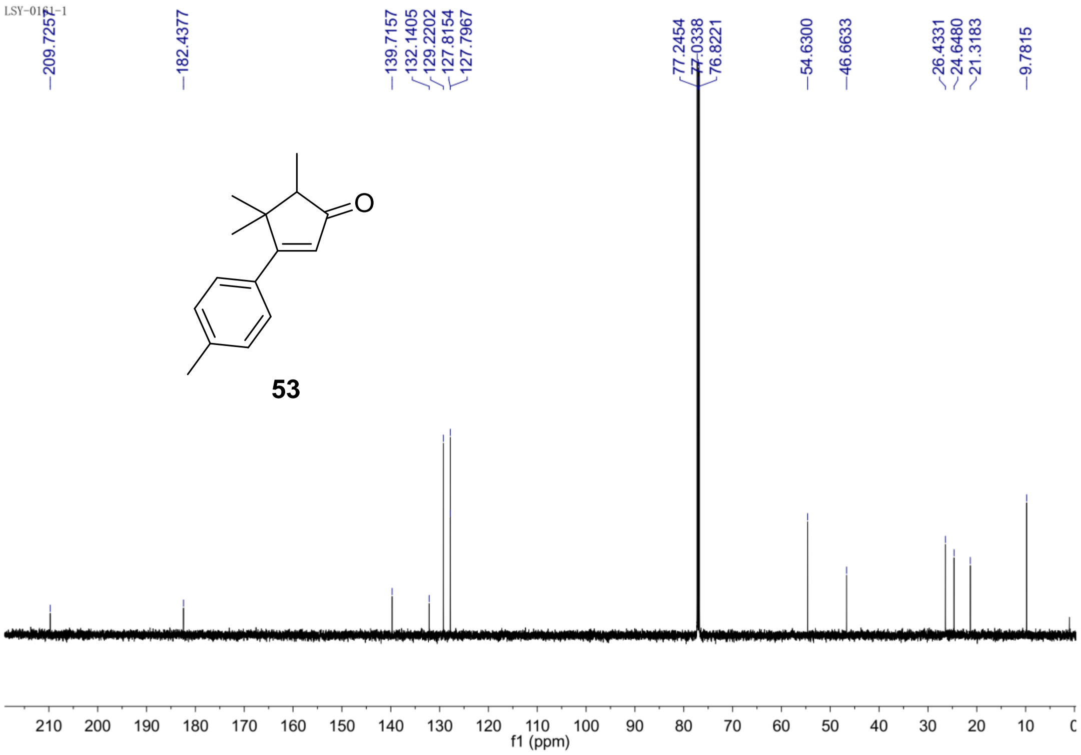

Supplementary Fig. 118. ^{13}C NMR spectrum (151 MHz, $CDCl_3$, 298K) of **53.**

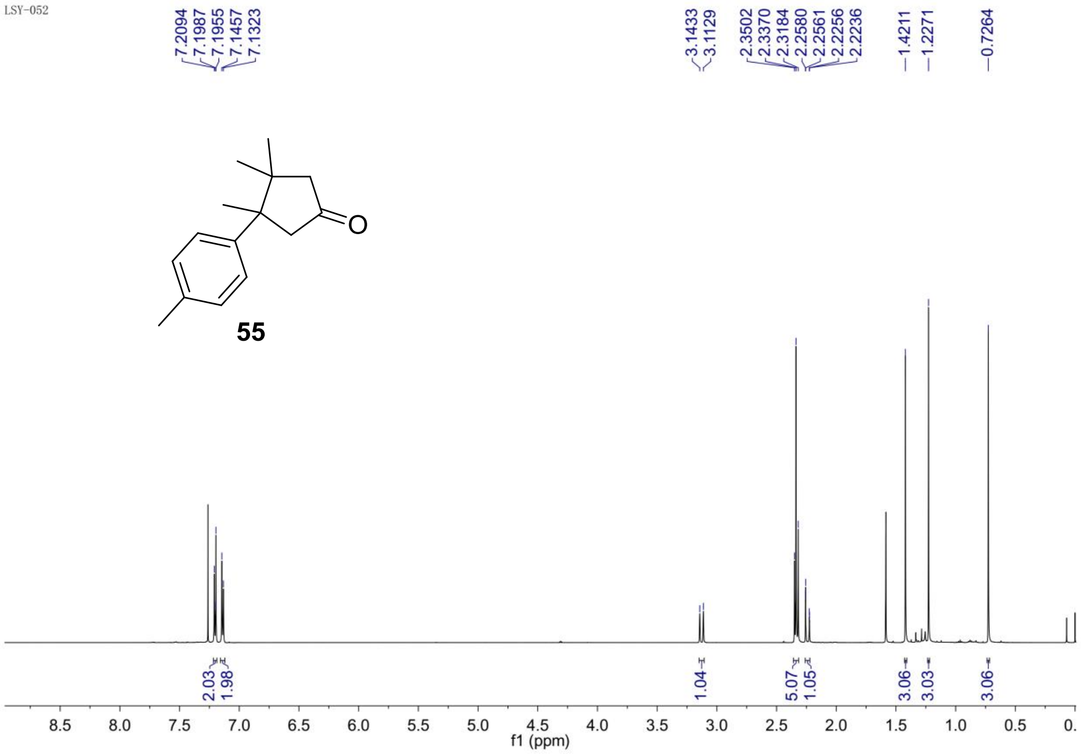

Supplementary Fig. 119. ^{1}H NMR spectrum (600 MHz, $CDCl_3$, 298K) of **55.**

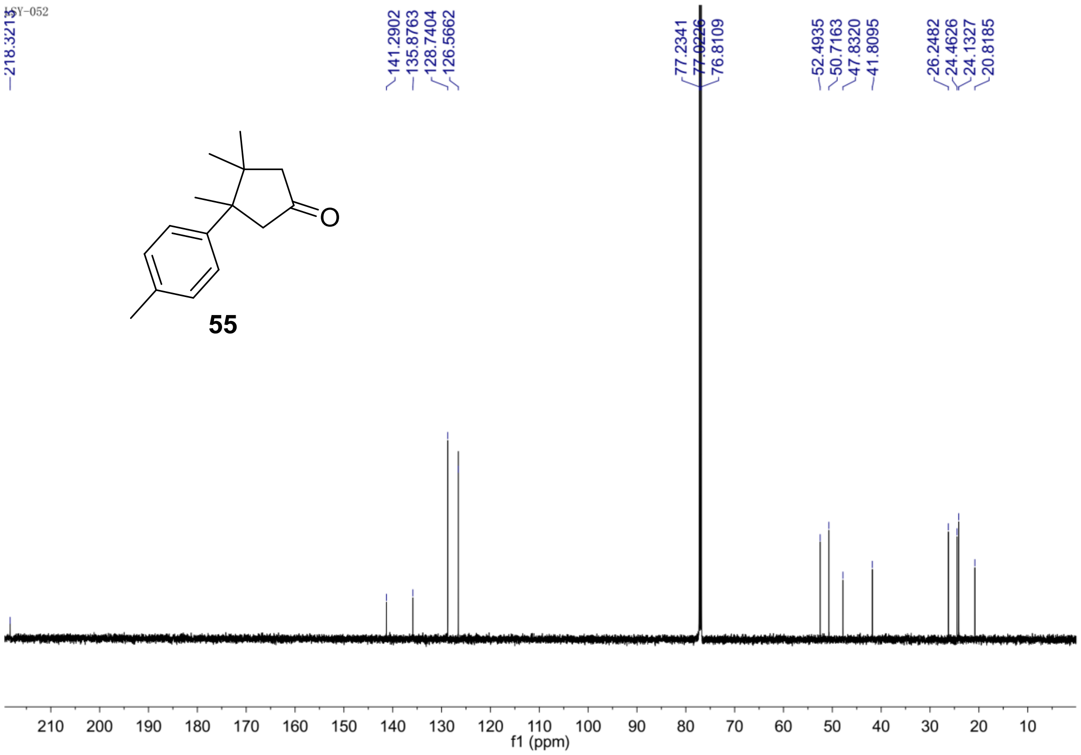

Supplementary Fig. 120. ^{13}C NMR spectrum (151 MHz, $CDCl_3$, 298K) of **55.**

3. Supplementary references

1. Chen, X., Kong, W., Cai, H., Kong, L. & Zhu, G. Palladium-catalyzed highly regio- and stereoselective synthesis of (1*E*)- or (1*Z*)-1,2-dihalo-1,4-dienes via haloallylation of alkynyl halides. *Chem. Commun*. **47**, 2164-2166 (2011).

2. Wang, B., Bonin, M. & Micouin, L. A straightforward synthesis of ynones by reaction of dimethylalkynylaluminum reagents with acid chlorides. *J. Org. Chem*. **70**, 6126-6128 (2005).

3. Naruse, A., Kitahara, K., lwasa, S. & Shibatomi, K. Synthesis of α-fluoroenones by elimination of α-chloro-α-fluoroketones. *Asian J. Org. Chem*. **8**, 691-693 (2019).

4. Das, M. K., Dinda, B. K. & Bisai, V. A unified approach to sesquiterpenes sharing trimethyl(p-tolyl) cyclopentanes: Formal total synthesis of (±)-laurokamurene B. *Tetrahedron Lett*. **60**, 2039-2042 (2019).

5. Goldman, P., Ramos, S. M. & Wuest, J. D. Reactions of nitroimidazoles with hydrazine. *J. Org. Chem*. **49**, 932-935 (1984).

6. Liu, M. T., Ho, J., Liu, J. K., Purakait, R., Morzan, U. N., Ahmed, L., Batista, V. S., Matsunami, H. & Ryan, K. Carbon chain shape selectivity by the mouse olfactory receptor OR-I7. *Org. Biomol. Chem*. **16**, 2541-2548 (2018).

7. Shu, W., Zhang, H. & Huang, Y. γ-Alkylation of alcohols enabled by visible-light induced 1,6-hydrogen atom transfer. *Org. Lett.* **21**, 6107-6111 (2019).
